# Supplementary material for: Experience of depression in older adults with and without a physical long-term condition: findings from a qualitative interview study
Source: BMJ Open. 2022 Feb 24;12(2):e056566. doi: 10.1136/bmjopen-2021-056566 (PMC8883274; doi:10.1136/bmjopen-2021-056566)
Supplement: Supplementary data [file bmjopen-2021-056566supp001.pdf]

Supplementary file: Appendix 1 - Vignettes

Scenario 1

Sarah has good days and bad, but on a bad day she feels so sad and miserable that she just can't stand it. It seems to completely take over her head like she's in a fog. She feels really disappointed in herself for feeling this way, despite all the good things she has in her life. Sometimes when she's by herself she cries and even has thoughts of killing herself just to try and be anywhere but here. She feels completely worthless.

Scenario 2

Most days Sam wakes up and feels so tired he wishes he could just stay in bed. In fact he feels so tired most of the time that he doesn't want to do anything. He sleeps longer than he used to and doesn't have the energy to do anything. Sometimes he doesn't even feel like eating. He's even lost weight without trying to. His partner tries to show him affection and wants a kiss and a cuddle, but he just feels numb and not interested at all.

Scenario 3

Jason finds it really hard to feel positive about the future at the moment, it's like he has nothing to look forward to anymore. He finds it difficult to concentrate and to make decisions these days too. He feels so frustrated and trapped by the way he feels. His friends tell him he should snap out of it, but he just can't. He wishes he didn't have to feel like this, it makes him feel like such a failure.

Scenario 4

Most days Clare feels like she is being punished for something she must have done in a past life. And then she feels guilty for thinking like this. She seems to worry about her health all the time and her aches and pains get on top of her. She doesn't enjoy things the way she used to, in fact she doesn't really get any pleasure out of anything anymore. She finds it hard to leave the house some days. Everything seems a bit pointless these days, if she's being completely honest.

Scenario 5

Susan has always enjoyed her food, but for the past couple of months she's noticed she's been putting on some extra weight. She finds she craves her favourite foods and is comfort eating to make herself feel a bit better. She's not sure she is feeling any better though, in fact she's sleeping really badly and often wakes up several times in the night. Her sleep is restless and she wakes up tired making each day feel like it drags.

Poole L, *et al. BMJ Open* 2022; 12:e056566. doi: 10.1136/bmjopen-2021-056566
