# Supplementary material for: Experience of depression in older adults with and without a physical long-term condition: findings from a qualitative interview study
Source: BMJ Open. 2022 Feb 24;12(2):e056566. doi: 10.1136/bmjopen-2021-056566 (PMC8883274; doi:10.1136/bmjopen-2021-056566)
Supplement: Supplementary data [file bmjopen-2021-056566supp002.pdf]

LONDON'S GLOBAL UNIVERSITY

DEPARTMENT OF BEHAVIOURAL SCIENCE AND HEALTH

UCL

The Mood and Health Study

Interview Schedule – Depression

Introduction

Review of Participant Information Sheet

Consent form

Timing and confidentiality

My name is [insert name] and I am a researcher. I'm not a doctor or a psychologist and I don't have any clinical contact with your team. I'm interested in finding out from you what your experiences have been with your mood, and there are no right answers.

Vignettes

To start I'd like you to look at what some other people have experienced, and then we'll talk about them a bit. Firstly, I'd like you to start by sorting the cards into two piles, those which you think are relevant to you and those which aren't.

**Card sort**	Can you talk me through how you have sorted the cards?<br>Has anything like this ever happened to you?<br>Have you ever felt these sorts of things before?<br>How do you think this person's experience is different from yours?

Depression

**Precipitating events**	Take me back, can you tell me about when you first noticed changes to your mood?

Poole L, *et al. BMJ Open* 2022; 12:e056566. doi: 10.1136/bmjopen-2021-056566

Prompts	• Can you remember feeling differently?<br>• How did it feel?<br>• Was there anything else you think may have contributed to your low mood?<br>• How do you think that experience relates to how you feel today?
**Diagnosis**	Can you talk me through the support you received during this time?
Prompts	• E.g. family, friends, GP?<br>• How did you find out you had depression? E.g. Who told you? When?<br>• How did you feel when you found out it was depression?
**Timeline**	How has your mood been since then?
Prompts	• How has you mood changed over time?
**Symptoms**	Can you tell me about a time when you felt your depression was at its worst?
Prompts	• How did it feel?<br>• How long did it last?<br>• Can you tell me how most days are for you at the moment?<br>• What's a good day for you?<br>• How long have you been feeling this way?<br>• Are there times when you don't feel this way? How long does this last?
**Management of depression**	Are you receiving any treatment for your depression at the moment?
Prompts	• Can you tell me about the care you have received for your depression from doctors?<br><br>e.g. who (GP, clinic nurse/doctor)<br>e.g. where (GP clinic, hospital clinic, community/third sector)<br>e.g. when (routine/booked appointments, length of appointments)

	e.g. what (antidepressants, counselling)<br>• Do you find it easy to talk to your doctor about your concerns?<br>• How did you find the treatment?<br>• How helpful has this treatment been? E.g. what was most helpful and least helpful?
**Self-care**	• Can you talk me through anything you do yourself that helps you cope better?
Prompts	• For example, exercise, eating habits, alcohol consumption, smoking, acceptance, distractions/hobbies, wallowing. Have any of these been helpful to you?<br>• How do these things make you feel?

Wrapping up

Is there anything else you would like to tell me?

How have you found this interview?

Do you have any questions?

Thank you for your time.

Give sources of further support handout.

Give baseline questionnaire.

Provide participant with £10 retail voucher.

LONDON'S GLOBAL UNIVERSITY
DEPARTMENT OF BEHAVIOURAL SCIENCE AND HEALTH
UCL

The Mood and Health Study

Interview Schedule – Depression and Physical Illness

Introduction

Review of Participant Information Sheet

Consent form

Timing and confidentiality

My name is [insert name] and I am a researcher. I'm not a doctor or a psychologist and I don't have any clinical contact with your team. I'm interested in finding out from you what your experiences have been with your mood and health, and there are no right answers.

Vignettes

To start I'd like you to look at what some other people have experienced, and then we'll talk about them a bit. Firstly, I'd like you to start by sorting the cards into two piles, those which you think are relevant to you and those which aren't.

**Card sort**	Can you talk me through how you have sorted the cards?<br>Has anything like this ever happened to you?<br>Have you ever felt these sorts of things before?<br>How do you think this person's experience is different from yours?

Depression

**Precipitating events**	Take me back, can you tell me about when you first noticed changes to your mood?

Prompts	• Can you remember feeling differently?<br>• How did you feel?<br>• Was there anything else you think may have contributed to your low mood?<br>• How do you think that experience relates to how you feel today?
**Diagnosis**	Can you talk me through the support you received during this time?
Prompts	• E.g. family, friends, GP?<br>• How did you find out you had depression? E.g. Who told you? When?<br>• How did you feel when you found out it was depression?
**Timeline**	How has your mood been since then?
Prompts	• How has you mood changed over time?
**Diagnosis of physical illness (if not covered above)**	Can you tell me how you found out about your [arthritis/heart disease/diabetes]?
Prompts	• How long ago was this?<br>• Who told you? Where were you?<br>• How did you feel when you found out?<br>• Did you find out about your [arthritis/heart disease/diabetes] before or after you found out you had depression? How did you find this time?
**Symptoms**	Can you tell me about a time when you felt your depression was at its worst?
Prompts	• How did it feel?<br>• How long did it last?<br>• Can you tell me how most days are for you at the moment?<br>• What's a good day for you?

	• How long have you been feeling this way?<br>• Are there times when you don't feel this way? How long does this last?
**Interaction**	Can you tell me about how your [arthritis/heart disease/diabetes] affects your life now?
	• How does your [arthritis/heart disease/diabetes] make you feel?<br>• How do your depression and [arthritis/heart disease/diabetes] affect each other?<br>• Have you ever found it difficult to cope with [arthritis/heart disease/diabetes]?<br>e.g. coping with the symptoms, treatments?
**Management**	Are you receiving any treatment for your depression at the moment?
Prompts	• Can you tell me about the care you have received for your depression from doctors?<br>e.g. who (GP, clinic nurse/doctor)<br>e.g. where (GP clinic, hospital clinic, community/third sector)<br>e.g. when (routine/booked appointments, length of appointments)<br>e.g. what (antidepressants, counselling)<br>• How did you find the treatment?<br>• How helpful has this treatment been? E.g. what was most helpful and least helpful?<br>• How easy have you found it to get help for your depression?
	What treatments have you had for your [arthritis/heart disease/diabetes]?
Prompts	e.g. who (GP, clinic nurse/doctor)<br>e.g. where (GP clinic, hospital clinic)<br>e.g. when (more than one treatment?)<br>e.g. what (self-management courses, medication, surgery?)

Poole L, *et al. BMJ Open* 2022; 12:e056566. doi: 10.1136/bmjopen-2021-056566

	• Do you find it easy to talk to your doctor about your concerns?<br>• How did you find the treatment?<br>• How helpful has this treatment been? E.g. what was most helpful and least helpful?<br>• How do you feel treatment of your [arthritis/heart disease/diabetes] has affected your depression?<br>• **[Arthritis and angina only]** How have the treatments helped with your pain?
**Self-care**	• Can you talk me through anything you do yourself that helps you cope better?
Prompts	• For example, exercise, eating habits, alcohol consumption, smoking, acceptance, distractions/hobbies, wallowing. Have any of these been helpful to you?<br>• How do these things make you feel?

Wrapping up

Is there anything else you would like to tell me?

How have you found this interview?

Do you have any questions?

Thank you for your time.

Give sources of further support handout.

Give baseline questionnaire.

Provide participant with £10 retail voucher.
