# Supplementary material for: Experience of depression in older adults with and without a physical long-term condition: findings from a qualitative interview study
Source: BMJ Open. 2022 Feb 24;12(2):e056566. doi: 10.1136/bmjopen-2021-056566 (PMC8883274; doi:10.1136/bmjopen-2021-056566)
Supplement: Author's manuscript [file bmjopen-2021-056566.draft_revisions.pdf]

BMJ Open is committed to open peer review. As part of this commitment we make the peer review history of every article we publish publicly available.

When an article is published we post the peer reviewers' comments and the authors' responses online. We also post the versions of the paper that were used during peer review. These are the versions that the peer review comments apply to.

The versions of the paper that follow are the versions that were submitted during the peer review process. They are not the versions of record or the final published versions. They should not be cited or distributed as the published version of this manuscript.

BMJ Open is an open access journal and the full, final, typeset and author-corrected version of record of the manuscript is available on our site with no access controls, subscription charges or pay-per-view fees (http://bmjopen.bmj.com).

If you have any questions on BMJ Open's open peer review process please email info.bmjopen@bmj.com

BMJ Open

The experience of depression in older adults with and without a physical long-term condition: findings from a qualitative interview study.

Journal:	*BMJ Open*
Manuscript ID	bmjopen-2021-056566
Article Type:	Original research
Date Submitted by the Author:	18-Aug-2021

Complete List of Authors:	Poole, Lydia; UCL, Institute of Health Informatics<br>Frost, Rachael; University College London Research Department of Primary Care and Population Health, Research Department of Primary Care and Population Health<br>Rowlands, Hannah<br>Black, Georgia; University College London, Applied Health Research; Dr

Keywords:	Depression & mood disorders < PSYCHIATRY, QUALITATIVE RESEARCH, Coronary heart disease < CARDIOLOGY, DIABETES & ENDOCRINOLOGY, RHEUMATOLOGY

SCHOLARONE™
Manuscripts

I, the Submitting Author has the right to grant and does grant on behalf of all authors of the Work (as defined in the below author licence), an exclusive licence and/or a non-exclusive licence for contributions from authors who are: i) UK Crown employees; ii) where BMJ has agreed a CC-BY licence shall apply, and/or iii) in accordance with the terms applicable for US Federal Government officers or employees acting as part of their official duties; on a worldwide, perpetual, irrevocable, royalty-free basis to BMJ Publishing Group Ltd ("BMJ") its licensees and where the relevant Journal is co-owned by BMJ to the co-owners of the Journal, to publish the Work in this journal and any other BMJ products and to exploit all rights, as set out in our licence.

The Submitting Author accepts and understands that any supply made under these terms is made by BMJ to the Submitting Author unless you are acting as an employee on behalf of your employer or a postgraduate student of an affiliated institution which is paying any applicable article publishing charge ("APC") for Open Access articles. Where the Submitting Author wishes to make the Work available on an Open Access basis (and intends to pay the relevant APC), the terms of reuse of such Open Access shall be governed by a Creative Commons licence – details of these licences and which Creative Commons licence will apply to this Work are set out in our licence referred to above.

Other than as permitted in any relevant BMJ Author's Self Archiving Policies, I confirm this Work has not been accepted for publication elsewhere, is not being considered for publication elsewhere and does not duplicate material already published. I confirm all authors consent to publication of this Work and authorise the granting of this licence.

For peer review only

The experience of depression in older adults with and without a physical long-term condition: findings from a qualitative interview study.

Lydia Poole[1], Rachael Frost[2], Hannah Rowlands[3], Georgia Black[4].

[1] Institute of Health Informatics, University College London, 222 Euston Road, London, NW1 2DA.

[2] Research Department of Primary Care and Population Health, Upper Third Floor, UCL Medical School (Royal Free Campus), Rowland Hill Street, London, NW3 2PF.

[3] Department of Psychological Medicine, Institute of Psychiatry, Psychology & Neuroscience, Kings College London, E3.18, De Crespigny Park, London, SE5 8AF.

[4] Department of Applied Health Research, University College London, 1-19 Torrington Place, London, WC1E 6BT.

Funding: LP and HR were funded by the UK Economic and Social Research Council (ES/N001478/1) for this project.

Declarations of interest: none.

Word count: 7694

Correspondence to:

Dr Lydia Poole

Institute of Health Informatics

222 Euston Road

University College London

London

NW1 2DA

T: +44 (0)20 3108 8817

E: lydia.poole@ucl.ac.uk

For peer review only - http://bmjopen.bmj.com/site/about/guidelines.xhtml

Abstract

Objective: To understand how the lived experience of depression differs among patients with a long-term condition (LTC) compared to those without an LTC, and how the experience differs across different types of LTC.

Design: Face-to-face, semi-structured, interviews.

Setting: Primary care; GP surgeries in and around North London.

Participants: 42 primary care patients with depression were recruited. Our sample comprised participants aged 55-75 years old with depression only (n= 12), depression and coronary heart disease (n=5), depression and type 2 diabetes (n=10) and depression and arthritis (n=14).

Results: Interviews were conducted, audio-recorded, transcribed, and analysed using thematic analysis. The results revealed that the cardinal diagnostic symptoms of depression (anhedonia, sadness) were experienced by all our participants regardless of LTC. However, the LTC did interact with depression by compounding somatic, cognitive and emotional symptoms, increasing disability and reducing independence, and hindering attempts at coping with mental illness. Our findings demonstrate common experiences across patients, as well as key differences based on LTC.

Conclusions: We suggest four key implications for future care practices of these patients: (1) Not all participants with depression and LTC view their mental and physical health as interconnected; there should be allowances in care plans for separate treatment pathways; (2) Key features of depression that affect LTC management are social withdrawal and lack of motivation to self-manage or access healthcare; (3) Key features of LTCs that worsen depression are pain, the unpredictability of future health and progressive disability; (4) Positive self-management of LTC could improve self-efficacy and therefore mood, and should be encouraged.

For peer review only - http://bmjopen.bmj.com/site/about/guidelines.xhtml

Key words: Depression; long-term conditions; co-morbidity; qualitative research; interview study; thematic analysis.

Strengths and limitations of the study

- Participant diagnoses derived from clinical records and confirmed by self-report.
- Participants were drawn from across multiple sites across North London, with diversity in demography achieved.
- Comparative thematic analysis allowing for comparisons within and between groups.
- Small sample of depression and coronary heart disease cohort.
- Majority female in the depression and arthritis cohort.

For peer review only

Introduction

Estimates from the UK Biobank study suggest the prevalence for a single episode of major depressive disorder is 6.4%, varying to 12.2% for moderate recurrent major depression and 7.2% for severe recurrent major depression (Smith et al., 2013), among British adults. Depression has been associated with increased all-cause mortality (Machado et al., 2018); however causality has not yet been determined (Roest & de Jonge, 2018). One possible explanation is that individuals with depression are more than twice as likely to have a concomitant physical long-term condition (LTC), such as diabetes and coronary heart disease.

It has been hypothesised from quantitative investigations of epidemiological and clinical cohort studies that depressive symptoms which occur in cardiac patients are distinct from those experienced in psychiatric populations based on three key differences: composition of symptoms, timeline, and treatment responsiveness (Poole et al., 2011). With regard to symptoms, the hallmarks of psychiatric depression are persistent sadness and loss of interest and pleasure in activities (anhedonia). These are often associated with cognitive changes such as a sense of worthlessness and guilt, among others. By contrast, there is growing recognition that depressive symptoms in people with physical illness are dominated by somatic symptoms, such as fatigue and loss of energy (de Miranda Azevedo et al., 2014; Poole & Jackowska, 2018). In addition, it has been suggested that the chronicity, and the timing of onset of depressive symptoms in relation to the LTC, have implications for morbidity and mortality (Dickens et al., 2008; White et al., 2016). Finally, it appears that different treatment strategies may be appropriate for those living with psychiatric depression compared to those who have comorbid depression (Gold et al., 2020), with particular benefit coming from integrative collaborative care models of healthcare (Knowles et al., 2015). Qualitative data has revealed that depression in physical illness has been shown to have been normalised by health care practitioners leading to barriers in its management (Coventry et al., 2011) and there may be resistance to antidepressant medication among some LTC patients (Simmonds et al., 2013). Given these empirical observations surrounding the differences in depression symptoms, timeline and treatment, some authors have suggested that a separate subtype of depression may exist, driven by biological (inflammatory) changes (Raison & Miller, 2011) which in turn are

For peer review only - http://bmjopen.bmj.com/site/about/guidelines.xhtml

implicated in LTCs (Poole & Steptoe, 2020). However, despite a plausible hypothesis for the existence of a separate subtype of depression in those with an LTC, whether or not this translates to differences in patient experience is unclear. Therefore, further qualitative research is warranted to examine whether the lived experience of depression is similar or different in patients with depression and/or a comorbid LTC.

Furthermore, experiences of depression across LTCs are likely to differ. A meta-synthesis of qualitative data has suggested that patients with depression and a comorbid LTC either see their mental and physical health as either separate and independent from each other, or interrelated with one having caused or worsened the other (DeJean et al., 2013). However, the similarities and/or differences in how depression is experienced in different LTCs is currently not well-understood. For example, depression in diabetes has been revealed to be heterogenous with aspects of diabetes-related distress and demoralisation being apparent (Gask et al., 2011), while depression in CHD has been associated with loss, both in terms of interpersonal loss as well as loss of health/control (Simmonds et al., 2013).

Therefore, the aim of this study was twofold. First, we aimed to examine how the lived experience of depression is similar or different between those with an LTC and those without, and second, we aimed to understand how experiences of depression are similar or different between types of LTC. In order to take into account the correlation between age and LTC onset, we focussed on the experiences of depression in adults in mid-late life.

Methods

Participant selection and recruitment

We recruited a total of 41 participants to this study using purposive sampling to capture a range of clinical and demographic characteristics. Thirty-seven participants were recruited using mailouts from 14 UK general practices in the Barnet and Enfield regions of North London; a further one participant was recruited using opportunistic sampling from GP surgeries in North West London; and a final three participants were recruited from the Camden and Islington IAPT (Improving Access to Psychological Therapy) service. Participants were male and female, aged 55-75 years old and English speaking. All participants were screened for current

For peer review only

For peer review only - http://bmjopen.bmj.com/site/about/guidelines.xhtml

depression by participating GP practices via electronic and hand searches of medical records. Current depression was defined as a patient who had either received a clinical code of depression by their GP and/or had received a referral or prescription for depression treatment in the preceding two months. All participants were medically stable outpatients living within the community. We excluded patients with a severe cognitive, mental or psychological impairment that would impede their ability to provide informed consent.

To compare patients living with depression, with and without an LTC, we recruited patients to one of four participant groups: depression only, depression and CHD, depression and T2D, and depression and arthritis. To reduce cross-over between the groups, participants with more than one LTC of interest (i.e. diabetes and arthritis) were excluded. We also excluded those with another LTC such as cancer, stroke and chronic lung disease to allow us to focus our discussions more easily on our chosen LTCs. The LTCs were selected based on *a priori* knowledge to elicit a variety of illness experiences: coronary heart disease can have an acute, life-threatening presentation (e.g. acute coronary syndromes); T2D involves monitoring and self-management of blood sugar levels and can lead to acute episodes of illness (hypoglycaemia); arthritis is associated with pain and functional impairment.

Practice GPs reviewed the list of those to approach prior to mailout and removed people that met the exclusion criteria or they considered inappropriate to contact. The mail-out included full details of the study and interested patients were invited to contact the research team to discuss participation. Patients were screened by researchers over the telephone using the same inclusion and exclusion criteria before arranging a convenient time and place for the interview to take place.

Interview data collection

We conducted semi-structured face-to-face interviews, mainly at participants' homes or in a meeting room at UCL (or another convenient location if requested), lasting approximately one hour (range 42–97 minutes). Interviews were carried out between May to October 2019. Interviews were conducted by HR (n = 40) or LP (n = 1), both health psychology researchers. The interviews began by presenting participants with four short vignettes for participants to read and reflect on. The vignettes contained the experiences of fictional

For peer review only - http://bmjopen.bmj.com/site/about/guidelines.xhtml

characters written in the third person (see Appendix 1) and were designed to encourage participants to reflect on their own symptoms of depression. This method is well-established for eliciting detailed information on sensitive topics in qualitative research (Hughes & Huby, 2012). The interview then covered a number of topics, to explore the experience of living with depression: diagnosis, precipitating events, symptoms, change in experience over time, management of depression and treatment preferences. In participants with a comorbid LTC we also explored the impact of depression on their physical illness and vice versa. The topic guides (see Appendix 2) were modified as interviews progressed, through team discussions. To circumvent issues surrounding stigma, initial questions steered clear of clinical labels, and instead invited participants to reflect on 'changes to their mood'. As each interview progressed the interviewer used the participants' own terminology. After each interview, demographic data and self-reported medical information were collected. Participants were provided with relevant local and national mental health service information and a £10 retail voucher honorarium. Any concerns over participant safety (including suicidal ideation and self-harm) were discussed with the senior researcher (LP) before contacting participants' GP with their consent. A Standard Operating Procedure was in place for this. Interviews were audio-recorded, with brief field notes, transcribed verbatim by an external company and anonymised and verified for accuracy by the research team (HR and LP).

Data analysis

We used reflexive thematic analysis (Braun & Clarke, 2006, 2021) to analyse our data from a realist/essentialist perspective in which a (mostly) one-way relationship is taken to operate between meaning, experience and language. All transcripts were read by HR and LP; GB and RF read a subsample. Data analysis was led by LP. Transcripts from each group were first read and analysed inductively, separately, to enable us to build an understanding of the idiographic qualities of the dataset (Braun & Clarke, 2006); inductive analysis is a bottom-up approach where the codes and themes are driven by the data and not by a theoretical framework. Each set of transcripts was read repeatedly, and codes were developed by LP in conjunction with HR. Codes were then grouped to construct themes; refined through team discussions and piloting. Coding of transcripts was performed using NVivo 12 (QSR International Pty Ltd., 2018) according to the thematic framework. After

For peer review only - http://bmjopen.bmj.com/site/about/guidelines.xhtml

For peer review only

conducting the initial analysis across the entire dataset, we tabulated the themes and subthemes using Excel (Microsoft for Windows 365) to allow us to systematically explore the similarities and differences in experiences across groups. This approach was informed by Framework Analysis (Ritchie & Spencer, 2002) and has been combined with thematic analysis by previous researchers conducting comparative qualitative analysis (Marcu et al., 2017). The indexing and charting of the data enabled us to maintain rigour in the analytic process. Themes were then written out and refined with feedback from team. The study was approved by NHS London – Fulham Research Ethics Committee (reference: 18/LO/2150).

[illegible]

Results

Participants

Out of 137 patients who expressed an interest in the study, 45 were eligible and 41 completed interviews. Participants were grouped into 4 cohorts based on their medical records and verified during screening telephone calls: depression only (n = 12), depression and CHD (n = 5) and depression and type 2 diabetes (T2D) (n = 10) and depression and arthritis (n = 14). In terms of CHD, patients in this group had received a diagnosis for myocardial infarction (n = 1), angina (n=2) and heart failure (n = 2). In terms of arthritis, patients in this group included those with rheumatoid arthritis (n = 2) and osteoarthritis (n = 12). Overall, participants were aged 63.5 years (standard deviation (SD) 5.5) and we sampled roughly equal numbers of men and women. However, the majority of depression and arthritis participants were female (93%). Approximately one-third of the sample were from a non-White ethnic group and 85.4% of all participants were taking antidepressant medication(s) at the time of the interview. The characteristics of the sample are presented in Table 1.

Thematic overview

We generated five overarching themes that encompass the experience of living with depression in those with and without an LTC. In each theme we have drawn out the relationship between the experience of depression in those with and without an LTC, and where relevant, compared this across the different LTC groups (CHD,

For peer review only

T2D and arthritis). Across all the themes, we found that the experience of depression and LTCs acted to compound the effects of each other; this was particularly apparent for those whose illness had resulted in changes to daily living. Figure 1 presents a schematic diagram of our themes.

Theme 1: Shaped by the course of time

Across our sample, depression was not static; it was shaped by individuals' life histories and past events. The experience of depression also shifted over time so that as people's lives progressed, different triggers for new episodes became more apparent. In those with an LTC, hardship brought about by their LTC and a loss of health was described by some as a trigger for depression.

Depression onset (first-ever episode)

In participants with and without LTCs, the onset of the first-ever episode of depression occurred at different ages; many could identify roots for their depression in childhood or adolescence, while others had received a diagnosis more recently. Across the groups, most participants were able to identify a precipitating adverse life event(s) to their first-ever depressive episode such as financial insecurity, (multiple) bereavement, a relationship breakdown, children taken into care, and physical and sexual abuse. For some, seemingly smaller grievances, had accumulated over time to initiate the first-ever episode of depression.

In contrast to the depression-only participants, in participants with an LTC, life histories revealed two separate discourses relating to the onset of the initial depression diagnosis: coincidental and interconnected. As such, for some, the LTC was coincidental to the onset of the depression, with the depression and LTC diagnoses occurring at different times and in relation to different precipitating/surrounding events. This discourse was predominately discussed by those with T2D, and to a lesser extent the CHD cohort. These individuals had a clear narrative about the onset of their depression, that was related to other life pressures such as caring responsibilities, fraud allegations and work rather to their LTC:

> "I don't think so. I don't really think so. I had no qualms about my health. It was the other things. The inferiority complex, the pressure of work and the unhappiness with my married life. It was those things." **(P.39, depression and T2D, male)**.

For peer review only - http://bmjopen.bmj.com/site/about/guidelines.xhtml

For peer review only

"I honestly can't say that [depression] is the reason of that [CHD]. But yes, when you have I suppose chest pain you think about it, you sit down, you [pause]. But I don't think it took me into depression at that time or anxiety or something." **(P.45, depression and CHD, male)**.

The second discourse highlighted the interconnected nature of the initial depression and LTC diagnosis. For these participants, depression was a new experience, and was directly attributed to their LTC, or its implications (e.g. hospitalisations, loss of mobility, job loss). The type of LTC did not make a difference, only whether the LTC was associated with unpredictable, major, and irreversible upheaval.

"Depressed? It was when I came out of hospital after having my triple bypass because my life changed completely. I lost my job, my relationship broke up, the kids and all that and I just sort of had a sort of breakdown and that was it really." **(P.44, depression and CHD, male)**.

Relapsing and episodic

Across all participants, after the initial onset, the experience of depression had a varying time course. A few participants had new onset depression. However, for the majority of participants, their history of depression was chronic, with repeated episodes over time. This had led to a familiarity with when a new 'bout' was coming on, even if there was no discernible trigger.

Where there was a clear trigger for a new episode of depression, this was often a difficult life event, such as a child going to prison or a transition to retirement. However, for some people with chronic, pre-existing depression, the LTC had also triggered a new episode. This led to a new experience of depression for these participants, which was not centred around their earlier difficult life circumstances which had originally brought about their depression. Instead, the LTC raised fears surrounding declining health, death and frailty, which fed into the new depressive episode:

"The depression since than that was different. It was felt trapped then … I was full of tension, full of uncomfortable feeling with him. I just couldn't, it was like having a snake you know circling around you know... So that is thank God I don't have that. The depression that I feel in this and that is because

For peer review only - http://bmjopen.bmj.com/site/about/guidelines.xhtml

of my health problems and that bothers me, that bothers me a lot." **(P.22, depression and CHD, female)**.

Theme 2: Somatisation

Somatic symptoms of depression were frequently experienced by our participants, in those with and without an LTC. The experience was largely similar across our four cohorts. Differences did exist in sleep disturbance and crying, with LTCs (mainly arthritis but also T2D) contributing to the experience of these somatic symptoms.

Universal somatic symptoms

Somatic symptoms were discussed by all our participants, irrespective of LTC. Symptoms discussed included appetite changes (including over- and under-eating), crying, and loss of intimacy. By far the most common of all the somatic complaints was sleep disturbance. Some described difficulties falling asleep, restless and disturbed sleep, vivid dreams and nightmares, and difficulty getting out of bed in the morning. Psychomotor retardation was another common somatic complaint linked to fatigue, which was experienced across all groups, irrespective of LTC.

> "Really, really very slow, very sluggish. Very physically, very physical. In fact, almost as physical as it is mental…. Yeah like you're swimming. Like you're swimming against a sea of something coming at you. And you're trying to wade yourself through and knowing that if you, and no energy, zero physical energy." **(P.13, depression only, female)**

Somatic symptoms and interactions with LTCs

For those with an LTC, the illness also directly contributed to the experience of somatic symptoms of depression. Sleep was negatively affected by the LTC through worries about managing the condition (as with P.2 with T2D) or pain. Pain was particularly associated with participants in the depression and arthritis cohort.

> "I haven't been a very good sleeper lately; I don't sleep the full 8 hours that they say you should do. I do think about things and one of the things obviously I do think about is make sure my diabetes is correct." **(P.2, depression and T2D, male)**.

For peer review only - http://bmjopen.bmj.com/site/about/guidelines.xhtml

"I can't sleep at night because of pain… And even at night I get a lot of pain in my back and that so I am just putting my legs round 10 times this way and 10 times that way because I can't sleep." **(P.4, depression and arthritis, female)**.

While crying was frequently described across all our participants, in our arthritis participants we observed a unique contribution of the LTC. Arthritis (and in particular arthritic pain) compounded the emotional and somatic experience of depression, and could prompt tears of fear and frustration.

"I am chopping up the onions, I have to go and sit down because the pain in my back is such that I can't, I have to sit down. And like I have cried with it, not just from the onions but real tears, through frustration I suppose and pain." **(P.41, depression and arthritis, female)**.

Theme 3: Cognitive and emotional burden

In this theme the participants' lived experience of the cognitive and emotional symptoms of depression are described. These key diagnostic symptoms of depression were clearly experienced by all our participants. However, we observed compounding and indirect routes linking LTCs with the cognitive and emotional burden of depression; moreover, thoughts of death were particularly prominent within the depression and CHD group.

Universal cognitive and emotional symptoms

Participants, regardless of LTC, had an overwhelming sense of disinterest in the world around them; they described a deep-rooted sadness, hopelessness, worthlessness and low self-esteem. Some felt as though they were being punished.

"You get what you deserve in life. And I don't deserve nothing. You know, I wasn't a good father when I was young. I was selfish. And it's come back and bit me up the bum, bit me on the bum. ..." **(P.12, depression only, male).**

Compounding effects of LTC on cognitive and emotional depressive symptoms

For peer review only - http://bmjopen.bmj.com/site/about/guidelines.xhtml

For some, the combination of the LTC and the depression led to a compounding effect on the cognitive and emotional depressive symptoms. In this way, the LTC directly contributed to the participants' experience of depression. This was particularly observed in those with T2D and arthritis.

> "I'm sitting there and taking the blood and giving myself injections and some days I just think, you know, how come it's come to this? And I know how it's come to this because, you know, I'm not managing my life in any sensible way, and I know that." **(P.25, depression and T2D, male).**

For participants with severe arthritic pain, pain was seen as an all-consuming and debilitating experience, which led to misery.

> "I'm getting pain back in my right knee and I'm going to have to eventually have that one done. It's really, oh god, what does that feel like? When I'm not feeling … oh ... it feels like having been almost injected with negativity." **(P.28, depression and arthritis, female).**

For P.32 her depression and the functional limitations imposed by her arthritis were difficult to unravel, with both acting in synergy to lead to a sense of hopelessness:

> "I'm also physically unable to do as much as I would like to do in terms of just - housework and shopping and it's difficult to pull it apart which is leading the sort of feelings of hopelessness and you know is it worth it and I can't, I might as well just stay in bed." **(P.32, depression and arthritis, female).**

Indirect effects of LTC upon cognitive and emotional depressive symptoms through affecting enjoyment of activities

For some of our LTC participants, the depression was a greater burden than their LTC, so cognitive and emotional depressive symptoms were experienced regardless of their LTC. In this way, some of our LTC participants experienced feelings of anhedonia, worthlessness, guilt, sadness, etc., similarly to the depression only participants. However, for others, the LTC (notably T2D and arthritis) fed into the experience of cognitive and emotional symptoms by removing their enjoyment of previously pleasurable activities.

An example comes from P.2, whose diabetes directly contributed to his feelings of anhedonia. This participant was particularly concerned about managing his blood glucose levels during social situations.

For peer review only - http://bmjopen.bmj.com/site/about/guidelines.xhtml

> "You are part of it because you are invited, you all go out, you are all friends, you have known one another for years and everything is there, but you don't feel as if you are really taking part if you get what I am saying. So that's the part of the mood I have found since I have been diagnosed with diabetes and that's the thing that holds me back from properly being 100% committed to enjoying it." **(P.2, depression and T2D, male).**

Pain also featured heavily in the descriptions of altered lifestyles recounted by our arthritis participants, perpetuating feelings of anhedonia.

> "That's, and I do enjoy my food, I do enjoy cooking. I mean I haven't had a dinner party now for over a year. I have always enjoyed doing that. But that's, and it's like washing up, you know I can do so much, […] but you know like my washing up is in stages. I will do this first, then go and sit down, come back and do that lot, the next lot, then go and sit down. […]. That is where the arthritis and depression, they are like, they join forces." **(P.41, depression and arthritis, female).**

Indirect effects of LTC on cognitive and emotional symptoms by causing a loss of control and independence

Financial insecurity and unequal personal relationships were experienced by many of our participants, irrespective of LTC, and had contributed to feelings of being out of control. Deteriorating physical health (particularly for those with arthritis) and increasingly frailty, had also led to feelings of loss of control for some. This was linked to feelings of loss of independence, which in turn led to feelings of depression.

> "Some of my clothes are high up, I can never reach them unless somebody is there, that makes me depressed having to rely on somebody all the time." **(P.4, depression and arthritis, female).**

Thoughts of dying

Suicidal ideation, thoughts of death (but not suicide) and actual suicide attempts were disclosed in participants with and without LTCs. Although the presence of an LTC did not influence suicidal thoughts, having CHD was an important leveller for these participants. These participants lived with the unpredictable nature of their illness and the threat of sudden and irreversible loss of health:

For peer review only - http://bmjopen.bmj.com/site/about/guidelines.xhtml

"Even now I still think about it because when I just took a deep breath here, I felt a bit of a twinge there and it's when I got told about it, it was very disturbing because I was shown the blockage […] and although I've been reassured that it won't go up, block my heart or anything like that, but it makes you wonder because you never know what might happen." **(P.30, depression and CHD, male).**

For P.44 his brush with death due to having a heart attack has given him a greater acceptance of death:

"Well I just didn't give a shit about anything anymore, you know, apparently according to them I died twice, so I didn't give a shit no more about it, death doesn't scare me no more." **(P.44, depression and T2D, male).**

For others with CHD, they were also having to confront death, but they discussed their fear surrounding a prolonged or sudden death:

"I keep saying to my sister, 'I don't care if I die actually in my sleep that will be fine. But if I don't die and I can't move, like I said to you if I can't move and I can't get up and can't call that horrifies me.' That is enough to, well you know." **(P.22, depression and CHD, female).**

"So, since that day, perhaps I have, it wasn't so much when I had the heart bypass, but since the [aortic] aneurism and everything else, […] I worry that every time I go to bed, I don't know if I'm going to wake up. You know. So, I worry about that." **(P.30, depression and CHD, male).**

Theme 4: Depression symptoms thwart self-care

In this theme we draw on the ways in which the experience of depression impeded our participants' ability to take care of themselves; many described not washing or brushing their teeth, not wanting to cook proper meals, and retreating from their social network. This was apparent for all our participants but added an extra layer of complexity to participants with an LTC.

Depression leads to lack of motivation to self-care

For peer review only - http://bmjopen.bmj.com/site/about/guidelines.xhtml

Depression was associated with an apathy which led to a loss of interest in behaviours that might lead to improved self-care. This was true of behaviours that might improve the depression itself such as engaging in social activities and exercising, as well as those important for managing LTCs.

The impact of depression on attempts to control diabetes could lead to self-neglect, both in terms of medication adherence and adherence to medical appointments.

> "I suppose the most obvious impact is there are days when I just can't be bothered to take my medication, both the medication for the depression and the insulin for my diabetes and I know it's stupid […]." **(P.25, depression and T2D, male).**
>
> "there are times now when I wouldn't not turn up for an appointment, absolutely, I just […] But yes, there are times when I just think "what's the bloody point?" in dealing with daily stuff." **(P.25, depression and T2D, male).**

Exercise was seen as an important self-care behaviour for both mental and physical health among our participants. However, P.32 described how her lack of motivation to exercise was contributing to her physical decline, which in turn compounded her feelings of depression.

> "As for the physical side of things, again, I suspect they would like [me] to say, I'll lose weight, get more exercise and it's very difficult when you don't want to go out of the house, particularly I haven't the confidence to go into a gym or to a swimming pool, it's not really, you know. […] So, and the other fear is, of course, the older I get, the general, you know, aging and infirmities, you know, yeah, it worries me greatly. You know, and it doesn't help the depression to lift. **(P.32, depression and arthritis, female).**

Social withdrawal prevents self-care behaviours

Many participants described how their attempts to cope had been thwarted by the depression itself. This was particularly true for those whose depression had led to social withdrawal. The retreat from daily life saw participants becoming withdrawn from both the outside world and their family and friends**.** Social withdrawal

For peer review only - http://bmjopen.bmj.com/site/about/guidelines.xhtml

impacted on participants' management of their LTC. This was observed particularly in those with T2D and arthritis.

> "I did find it hard to leave the house sometimes, you know. You'd just sort of eat what was there rather than go shopping, you know." **(P.27, depression and T2D, female).**

> "Well they said because of the way, it's not helped, because I stopped, I wouldn't get out of the house, I was staying in my room, that can affect arthritis because then you are letting it seize up more." **(P.3, depression and arthritis, female).**

As seen with those experiencing a lack of motivation, social withdrawal also negatively impacted participants' willingness to engage with medical professionals (both for depression and/or the LTC).

> "I mean even the doctor, I have got to phone her tomorrow, they are going to arrange for the nurse to come out because I am overdue blood tests and blood pressure and that […]. Because she knows that I just don't want to leave the house again." **(P.3, depression and arthritis, female).**

Theme 5: Requires wilful effort to manage

The experience of depression was described as an active process, requiring an individual to find ways to cope. For some with an LTC, efforts to cope with their depression were thwarted by the functional limitation imposed by their illness. Controlling the LTC was also important to help manage the depression.

Universal coping strategies

While many of our participants struggled to cope, nearly all engaged in some strategies to navigate their current situation such as the use of alcohol and food, medical treatments, and drawing on friendships and family members for support. Often this included attempts to mask feelings as a means to find comfort; alcohol in particular was frequently described as a way to relax and forget.

> "But what I have done, and I have been guilty of, is purchasing alcohol and trying to blot it out with the alcohol." **(P.23, depression and arthritis, female).**

For peer review only

For peer review only - http://bmjopen.bmj.com/site/about/guidelines.xhtml

Many participants were engaged with healthcare professionals for depression management. For some anti-depressants had been beneficial, making their symptoms less severe. However, for others, side effects or fears surrounding dependency prevented their use.

> "Most of us are given medication, which makes you zombie, anyway. It doesn't help you. It just block your pains, you know. You are numb. You are not sorting out. And how long are you going to use those medication?" **(P.24, depression and T2D, female).**

LTCs impede efforts to cope with depression

Having an LTC prevented some participants from engaging with coping strategies to help manage their depression. For those with arthritis this included being unable to take part in activities that promote wellbeing, including hobbies.

> "But I think, yeah, so coping, well I am not walking as much as I want to, I have stopped going, I used to go to water workout, water aerobics every week and I have stopped doing that, partly because of the difficulty of getting into my bathers and afterwards drying myself." **(P.1, depression and arthritis, female).**

As P.32 described, the combination of her depression and arthritis makes coping with each insurmountable:

> "You know so the depression makes you lack motivation to, perhaps, I say modify some of the effects of the arthritis, but the arthritis also, in limiting you, makes the depression seem worse. Together it seems like a huge, insurmountable problem because if, on their own, they might be manageable, […]. So it is, yes, it impedes any kind of self-care or therapy, if you like, together."

Controlling the LTC improves depression

Interestingly across all LTC groups, those who successfully managed to engage with strategies to cope with their illness, saw benefits to their mental health through feeling an increased sense of control/self-efficacy. For example, failure to self-manage insulin dosages appropriately negatively impacted P.25's mood, while being in control of his T2D also helped him self-manage his depression.

For peer review only - http://bmjopen.bmj.com/site/about/guidelines.xhtml

"But I carry glucose around with me. … I think we were away somewhere and, but I was completely useless for about two hours and I suppose I, you know, I had a moment of clarity about the need to keep on top of it properly, because I sort of knew that if I didn't, not only would I feel crap, physically, the idea of feeling crap physically was depressing." **(P.25, depression and T2D, male).**

Interestingly, P.30 with CHD found the unpredictable nature of his cardiac symptoms very distressing, but the use of his own blood pressure monitor helped him regain a sense of control.

"this is my life saver, my BP [blood pressure] machine, I do it every day, […] I was told 140 over 80, right, yes, 140, 80, so once it's within this, I go to sleep a bit better. If I didn't know what my BP was, I'd be worried even more." **(P.30, depression and CHD, male).**

For some participants the physical limitations imposed by their LTC led to social isolation. This was particularly apparent for those with arthritis. If participants received support with things that their arthritis prevented (for example housework, food shopping), this was seen as positive for managing their depression, allowing them to live well within the limits of the LTC.

"So yeah, [lady friend's name] and [man friend's name] took me out yesterday to go to Aldi, it's the first time since February that I have gone out to the shops." **(P.3, depression and arthritis, female).**

Discussion

This paper explored the lived experience of depression in primary care patients with and without a comorbid physical LTC (CHD, T2D and arthritis). Using thematic analysis, we generated five overarching themes. 'Shaped by the course of time' revealed a shifting, non-linear experience of depression in which the experience was defined by earlier life experiences as well as more minor, cumulative events. For some, the LTC diagnosis had caused catastrophic upheaval to participants' lives; for these individuals the LTC was capable of triggering depression (both first-ever or a recurrent episode). For many, other life events were more prominent to determining the course of depression; for these participants the LTC was coincidental, running alongside the depression but not triggering new episodes. 'Somatisation' highlighted the physical experience of depression

For peer review only - http://bmjopen.bmj.com/site/about/guidelines.xhtml

in our participants; for many, these symptoms were as dominant as the cognitive and emotional symptoms. Somatic symptoms were universally experienced across all our groups, in particular feelings of tiredness and fatigue and changes to eating habits. Where LTCs did interact with the somatic symptoms, this was mainly driven by arthritic pain contributing to and compounding these symptoms. ‘Cognitive and emotional burden’ explored the impact of mental symptoms, such as feelings of worthlessness, guilt, punishment, anhedonia and sadness, on participants. LTCs compounded these cognitive and emotional experiences as well as indirectly contributing to a worsening of these symptoms by affecting participants’ enjoyment of previously pleasurable activities and causing a loss of control and independence. Thoughts of dying were frequently experienced, and prominent in patients with CHD where the unpredictability of future health was a psychological challenge. Theme 4, ‘Depression symptoms thwart self-care’ examined how depression itself acts as a barrier to engagement in self-care behaviours. Loss of motivation and social withdrawal and were key aspects of the depressive experience which thwarted attempts for LTC self-management (particularly those with T2D). Finally, ‘Requires wilful effort to manage’ revealed universal engagement in many coping strategies, particularly attempts to mask depressive feelings such as the use of alcohol and food. However, LTCS (particularly arthritis) did impede participants’ ability to cope with depression, while successful management of the LTC had a positive impact on mood.

Findings from quantitative studies continue to make attempts to understand the relationship between depression in LTCs, with the causal model thought to be bidirectional (Gold et al., 2020). Our findings supported the notion that the LTC was capable of triggering depression; however, this was restricted to those whose LTC has caused major life upheaval (e.g. loss of employment, relationship breakdown). It has been estimated that around 80% of initial depressive episodes occur after a stressful life event (Mazure, 1998); and that the severity of stressors required to precipitate recurrent depressive episodes decreases over time (Tennant, 2002). None of our participants described their depression acting as a precursor to their LTC. In fact, for many, the LTC was experienced as coincidental to the onset of depression; this is a view that has been supported by a previous qualitative meta-synthesis (DeJean et al., 2013).

For peer review only - http://bmjopen.bmj.com/site/about/guidelines.xhtml

The difference in the experience of depression in those with and without an LTC has not been studied previously using qualitative research. Quantitative research has identified differences in terms of symptom presentation, chronicity and timing of symptoms, and treatment responsivity (Poole et al., 2011). Our findings have added to this by revealing that while the symptoms of depression (somatic, cognitive, affective) were universally experienced by those with and without depression, the LTC contributed to the burden of these symptoms by either directly provoking them (e.g. arthritic pain leading to tears of frustration) or by indirectly leading to negative mood by undermining the enjoyment of partaking in previously pleasurable activities as well as reducing individuals' sense of independence. T2D and arthritis were key in this regard since both illnesses were associated with lifestyle changes which negatively impacted the participants' depression, increasing feelings of anhedonia, sadness, guilt and worthlessness. Previous research has explored the impact of arthritis on leisure activities, finding that functional and physical constraints imposed by the illness can affect self-esteem (Wikström et al., 2005). Our findings extend this to the experience of those with arthritis *and* depression, suggesting that these limitations worsen the symptoms of depression. Participants with diabetes also saw a compounding effect of their physical condition on their mental health. This was particularly true for those who described being distressed by T2D and its management. Indeed, diabetes distress is now recognised as a separate disorder to major depression (Kreider, 2017) which has been linked to feelings of burden, difficulties adhering to medication regimes and depression (Tanenbaum et al., 2016).

Loss of control and lack of independence featured heavily in the accounts of our participants with arthritis; whereby functional limitations imposed by the LTC indirectly impacted depression by removing individual's sense of control and autonomy. Loss was a key theme identified in the qualitative UPBEAT-UK study of patients with depression and CHD, where patients described not only loss of health but also interpersonal loss (Simmonds et al., 2013). Health deterioration featured heavily in the accounts of all our participants with a physical LTC, and while thoughts of dying were universally experienced across groups, our CHD group revealed a strong sense of an unpredictable and uncontrollable death, which was not apparent in those with T2D and arthritis. This realistic fear underpinned the experience of depression in these participants.

For peer review only - http://bmjopen.bmj.com/site/about/guidelines.xhtml

Our findings also observed differences in the experience of depression in participants' ability to self-care and to cope with their mental health. Social withdrawal and lack of motivation were commonly experienced in all our participants. We described how these features of depression thwart attempts to engage with self-care behaviours such as personal hygiene, meal preparation etcetera, as well as LTC (particularly T2D) specific illness-management behaviours such as adhering to medication and attending hospital appointments. While depression has been associated with poor medication adherence in previous quantitative studies of T2D and CHD patients (Carney et al., 1995; Gonzalez et al., 2007), there has been a lack of qualitative studies describing the hows and whys of this phenomenon; more research in this area is warranted.

Findings from our study revealed the adoption of common coping behaviours across all our participants such as using alcohol and food to mask symptoms and drawing on expert sources of support; this supports findings from earlier work (Simmonds et al., 2013). However, importantly we observed that the presence of an LTC interacted with participants' attempts to manage their depression. Those with an LTC, and in particular those with increasing disability from their arthritis, were prevented from engaging with coping mechanisms they had previously adopted to promote mental wellbeing. On the upside, positive self-management of the LTC improved feelings of control and self-efficacy and in so doing improved depressed mood. Depressed patients have previously been reported to benefit most from self-efficacy gains from LTC self-management training (Jerant et al., 2008); the importance of this construct for therapeutic purposes within the modern care setting of depression and LTC patients, requires future investigation.

Our findings have a number of important implications for care models such as the NHS' IAPT-LTC programme (NHS England, 2018). Our findings suggest that any care model of depression in LTC needs to take into account four overarching conclusions based on our data: (1) Not all participants with depression and LTC view their mental and physical health as interconnected; therefore there should be allowances in care plans for separate treatment (this is a view endorsed by Knowles et al., (2015)). (2) Key features of depression that affect LTC management and need to be considered when planning treatment and referring to psychological services are social withdrawal and lack of motivation to self-manage or access healthcare. (3) Key features of LTCs that

For peer review only - http://bmjopen.bmj.com/site/about/guidelines.xhtml

worsen depression that should be discussed during care planning are pain, the unpredictability of future health and progressive disability. (4) Positive self-management of LTC could improve feelings of control and self-efficacy and therefore mood, and should be encouraged.

A strength of our study was in the recruitment of participants from GP practices ensuring all our participants had received clinical diagnoses of their health conditions. To understand the differences in depression across different LTCs we excluded participants with more than one LTC of interest and with a serious or life-limiting diagnosis such as cancer, stroke or dementia. Due to the increased risk of physical illness with age, we recruited participants within an age bracket of 55 to 75 years; this enabled us to focus on the experiences of adults in mid to late life. The study was confined to North and North West London; however, we recruited from across contrasting areas to elicit a range of experiences. Despite our best efforts, unfortunately our arthritis sample was predominately female (90%); however this may partly reflect the greater incidence of osteoarthritis in women in the UK (Swain et al., 2020). Our depression and CHD group was small (n=5); therefore our findings may not reflect the diversity of views that a larger sample might have attained.

In conclusion, the results of our face-to-face interview study of depression in those with and without a physical LTC revealed that overall, key diagnostic symptoms of depression (anhedonia, sadness etc.) were experienced by all our participants regardless of LTC. However, the LTC did interact with depression by compounding somatic, cognitive, and emotional symptoms, increasing disability and reducing independence, and hindering attempts at coping with mental illness. Our findings have implications for the development and fine-tuning of mental health and LTC care programmes.

For peer review only - http://bmjopen.bmj.com/site/about/guidelines.xhtml

For peer review only

References

Braun, V., & Clarke, V. (2006). Using thematic analysis in psychology. *Qualitative Research in Psychology*, *3*(2), 77–101. https://doi.org/10.1191/1478088706qp063oa

Braun, V., & Clarke, V. (2021). Can I use TA? Should I use TA? Should I not use TA? Comparing reflexive thematic analysis and other pattern-based qualitative analytic approaches. *Counselling and Psychotherapy Research*, *21*(1), 37–47.

Carney, R. M., Freedland, K. E., Eisen, S. A., Rich, M. W., & Jaffe, A. S. (1995). Major depression and medication adherence in elderly patients with coronary artery disease. *Health Psychology*, *14*(1), 88–90. https://doi.org/10.1037/0278-6133.14.1.88

Coventry, P. A., Hays, R., Dickens, C., Bundy, C., Garrett, C., Cherrington, A., & Chew-Graham, C. (2011). Talking about depression: A qualitative study of barriers to managing depression in people with long term conditions in primary care. *BMC Family Practice*, *12*, 10. https://doi.org/10.1186/1471-2296-12-10

de Miranda Azevedo, R., Roest, A. M., Hoen, P. W., & de Jonge, P. (2014). Cognitive/affective and somatic/affective symptoms of depression in patients with heart disease and their association with cardiovascular prognosis: A meta-analysis. *Psychological Medicine*, *44*(13), 2689–2703. https://doi.org/10.1017/S0033291714000063

DeJean, D., Giacomini, M., Vanstone, M., & Brundisini, F. (2013). Patient Experiences of Depression and Anxiety with Chronic Disease. *Ontario Health Technology Assessment Series*, *13*(16), 1–33.

Dickens, C., McGowan, L., Percival, C., Tomenson, B., Cotter, L., Heagerty, A., & Creed, F. (2008). New onset depression following myocardial infarction predicts cardiac mortality. *Psychosomatic Medicine*, *70*(4), 450–455. https://doi.org/10.1097/PSY.0b013e31816a74de

Gask, L., Macdonald, W., & Bower, P. (2011). What is the relationship between diabetes and depression? A qualitative meta-synthesis of patient experience of co-morbidity. *Chronic Illness*, *7*(3), 239–252. https://doi.org/10.1177/1742395311403636

For peer review only - http://bmjopen.bmj.com/site/about/guidelines.xhtml

Gold, S. M., Köhler-Forsberg, O., Moss-Morris, R., Mehnert, A., Miranda, J. J., Bullinger, M., Steptoe, A., Whooley, M. A., & Otte, C. (2020). Comorbid depression in medical diseases. *Nature Reviews Disease Primers*, *6*(1), 1–22. https://doi.org/10.1038/s41572-020-0200-2

Gonzalez, J. S., Safren, S. A., Cagliero, E., Wexler, D. J., Delahanty, L., Wittenberg, E., Blais, M. A., Meigs, J. B., & Grant, R. W. (2007). Depression, Self-Care, and Medication Adherence in Type 2 Diabetes: Relationships across the full range of symptom severity. *Diabetes Care*, *30*(9), 2222–2227. https://doi.org/10.2337/dc07-0158

Hughes, R., & Huby, M. (2012). The construction and interpretation of vignettes in social research. *Social Work and Social Sciences Review*, *11*(1), 36–51.

Jerant, A., Kravitz, R., Moore-Hill, M., & Franks, P. (2008). Depressive Symptoms Moderated the Effect of Chronic Illness Self-Management Training on Self-Efficacy. *Medical Care*, *46*(5), 523–531.

Knowles, S. E., Chew-Graham, C., Adeyemi, I., Coupe, N., & Coventry, P. A. (2015). Managing depression in people with multimorbidity: A qualitative evaluation of an integrated collaborative care model. *BMC Family Practice*, *16*, 32. https://doi.org/10.1186/s12875-015-0246-5

Kreider, K. E. (2017). Diabetes Distress or Major Depressive Disorder? A Practical Approach to Diagnosing and Treating Psychological Comorbidities of Diabetes. *Diabetes Therapy*, *8*(1), 1–7. https://doi.org/10.1007/s13300-017-0231-1

Machado, M. O., Veronese, N., Sanches, M., Stubbs, B., Koyanagi, A., Thompson, T., Tzoulaki, I., Solmi, M., Vancampfort, D., Schuch, F. B., Maes, M., Fava, G. A., Ioannidis, J. P. A., & Carvalho, A. F. (2018). The association of depression and all-cause and cause-specific mortality: An umbrella review of systematic reviews and meta-analyses. *BMC Medicine*, *16*(1), 112. https://doi.org/10.1186/s12916-018-1101-z

Marcu, A., Black, G., Vedsted, P., Lyratzopoulos, G., & Whitaker, K. L. (2017). Educational differences in responses to breast cancer symptoms: A qualitative comparative study. *British Journal of Health Psychology*, *22*(1), 26–41. https://doi.org/10.1111/bjhp.12215

For peer review only - http://bmjopen.bmj.com/site/about/guidelines.xhtml

Mazure, C. M. (1998). Life stressors as risk factors in depression. *Clinical Psychology: Science and Practice*, *5*(3), 291–313.

NHS England. (2018). *The Improving Access to Psychological Therapies (IAPT) Pathway for People with Long-term Physical Health Conditions and Medically Unexplained Symptoms*. NHS England. https://www.england.nhs.uk/wp-content/uploads/2018/03/improving-access-to-psychological-therapies-long-term-conditions-pathway.pdf

Poole, L., Dickens, C., & Steptoe, A. (2011). The puzzle of depression and acute coronary syndrome: Reviewing the role of acute inflammation. *Journal of Psychosomatic Research*, *71*(2), 61–68. https://doi.org/10.1016/j.jpsychores.2010.12.009

Poole, L., & Jackowska, M. (2018). The association between depressive and sleep symptoms for predicting incident disease onset after 6-year follow-up: Findings from the English Longitudinal Study of Ageing. *Psychological Medicine*, 1–10. https://doi.org/10.1017/S0033291718001290

Poole, L., & Steptoe, A. (2020). The combined association of depressive symptoms and C-reactive protein for incident disease risk up to 12 years later. Findings from the English Longitudinal Study of Ageing (ELSA). *Brain, Behavior, and Immunity*, *88*, 908–912.

Raison, C. L., & Miller, A. H. (2011). Is Depression an Inflammatory Disorder? *Current Psychiatry Reports*, *13*(6), 467–475. https://doi.org/10.1007/s11920-011-0232-0

Ritchie, J., & Spencer, L. (2002). Qualitative data analysis for applied policy research. *The Qualitative Researcher's Companion*, 305–329.

Roest, A. M., & de Jonge, P. (2018). The heart of the matter: In search of causal effects of depression on somatic diseases. *BMC Medicine*, *16*(1), 147. https://doi.org/10.1186/s12916-018-1144-1

Simmonds, R. L., Tylee, A., Walters, P., & Rose, D. (2013). Patients' perceptions of depression and coronary heart disease: A qualitative UPBEAT-UK study. *BMC Family Practice*, *14*, 38. https://doi.org/10.1186/1471-2296-14-38

Smith, D. J., Nicholl, B. I., Cullen, B., Martin, D., Ul-Haq, Z., Evans, J., Gill, J. M. R., Roberts, B., Gallacher, J., Mackay, D., Hotopf, M., Deary, I., Craddock, N., & Pell, J. P. (2013). Prevalence and Characteristics of

For peer review only - http://bmjopen.bmj.com/site/about/guidelines.xhtml

Probable Major Depression and Bipolar Disorder within UK Biobank: Cross-Sectional Study of 172,751 Participants. *PLOS ONE*, *8*(11), e75362. https://doi.org/10.1371/journal.pone.0075362

Swain, S., Sarmanova, A., Mallen, C., Kuo, C. F., Coupland, C., Doherty, M., & Zhang, W. (2020). Trends in incidence and prevalence of osteoarthritis in the United Kingdom: Findings from the Clinical Practice Research Datalink (CPRD). *Osteoarthritis and Cartilage*, *28*(6), 792–801. https://doi.org/10.1016/j.joca.2020.03.004

Tanenbaum, M. L., Kane, N. S., Kenowitz, J., & Gonzalez, J. S. (2016). Diabetes distress from the patient's perspective: Qualitative themes and treatment regimen differences among adults with type 2 diabetes. *Journal of Diabetes and Its Complications*, *30*(6), 1060–1068. https://doi.org/10.1016/j.jdiacomp.2016.04.023

Tennant, C. (2002). Life Events, Stress and Depression: A Review of Recent Findings. *Australian & New Zealand Journal of Psychiatry*, *36*(2), 173–182. https://doi.org/10.1046/j.1440-1614.2002.01007.x

White, J., Zaninotto, P., Walters, K., Kivimäki, M., Demakakos, P., Biddulph, J., Kumari, M., De Oliveira, C., Gallacher, J., & Batty, G. D. (2016). Duration of depressive symptoms and mortality risk: The English Longitudinal Study of Ageing (ELSA). *The British Journal of Psychiatry: The Journal of Mental Science*, *208*(4), 337–342. https://doi.org/10.1192/bjp.bp.114.155333

Wikström, I., Jacobsson, L. T., & Arvidsson, B. (2005). How people with rheumatoid arthritis perceive leisure activities: A qualitative study. *Musculoskeletal Care*, *3*(2), 74–84. https://doi.org/10.1002/msc.28

Author Contributions:

LP was responsible for funding acquisition and conceived the study design. GB and RF advised on the study methods. LP and HR collected the data. LP and RF led the data analysis. GB and HR contributed to the data analysis. LP wrote the initial manuscript draft. RF, GB and HR edited the manuscript.

For peer review only - http://bmjopen.bmj.com/site/about/guidelines.xhtml

Table 1: Characteristics of the sample, stratified by group

	Whole sample (N= 41) *M±SD or N(%)*	Depression only (n = 12) *M±SD or N(%)*	Depression and CHD (n = 5) *M±SD or N(%)*	Depression and T2D (n = 10 *M±SD or N(%)*	Depression and arthritis (n = 14) *M±SD or N(%)*
Sex (female)	24(58.5)	6(50.0)	1(20.0)	4(40.0)	13(92.9)
Age (years)	63.5(5.5)	62.4(5.5)	66.0(4.0)	61.0(5.2)	65.1(5.9)
Married/cohabiting	17(41.5)	6(50.0)	2(40.0)	5(50.0)	4(28.6)
Ethnicity:					
White British	27(65.9)	10(83.3)	2(20.0)	6(60.0)	9(64.3)
Indian	3(7.3)			2(20.0)	1(7.1)
Pakistani	1(2.4)		1(20.0)		
Black Caribbean	1(2.4)		1(20.0)		
Other White background	7(17.1)	2(16.7)	1(20.0)	1(10.0)	3(21.4)
Other Asian background	2(4.9)			1(10.0)	1(7.0)
Employment status:					
Employed full-time	9(22.0)	3(25.0)	1(20.0)	3(30.0)	2(14.3)
Employed part-time	1(2.4)	1(8.3)			
Self-employed	7(17.1)	3(25.0)			4(28.6)
Retired	15(36.6)	4(33.3)	3(60.0)	3(30.0)	5(35.7)
Unemployed	4(9.8)	1(8.3)		3(30.0)	
Volunteer	2(4.9)		1(20.0)		1(7.1)
Disabled	3(7.3)			1(10.0)	2(14.3)
Highest educational qualification - degree or higher	15(36.6)	3(25.0)	3(60.0)	4(40.0)	5(35.7)
Antidepressant usage (yes)	35(85.4)	9(75.0)	4(80.0)	9(90.0)	13(92.9)

For peer review only

For peer review only - http://bmjopen.bmj.com/site/about/guidelines.xhtml

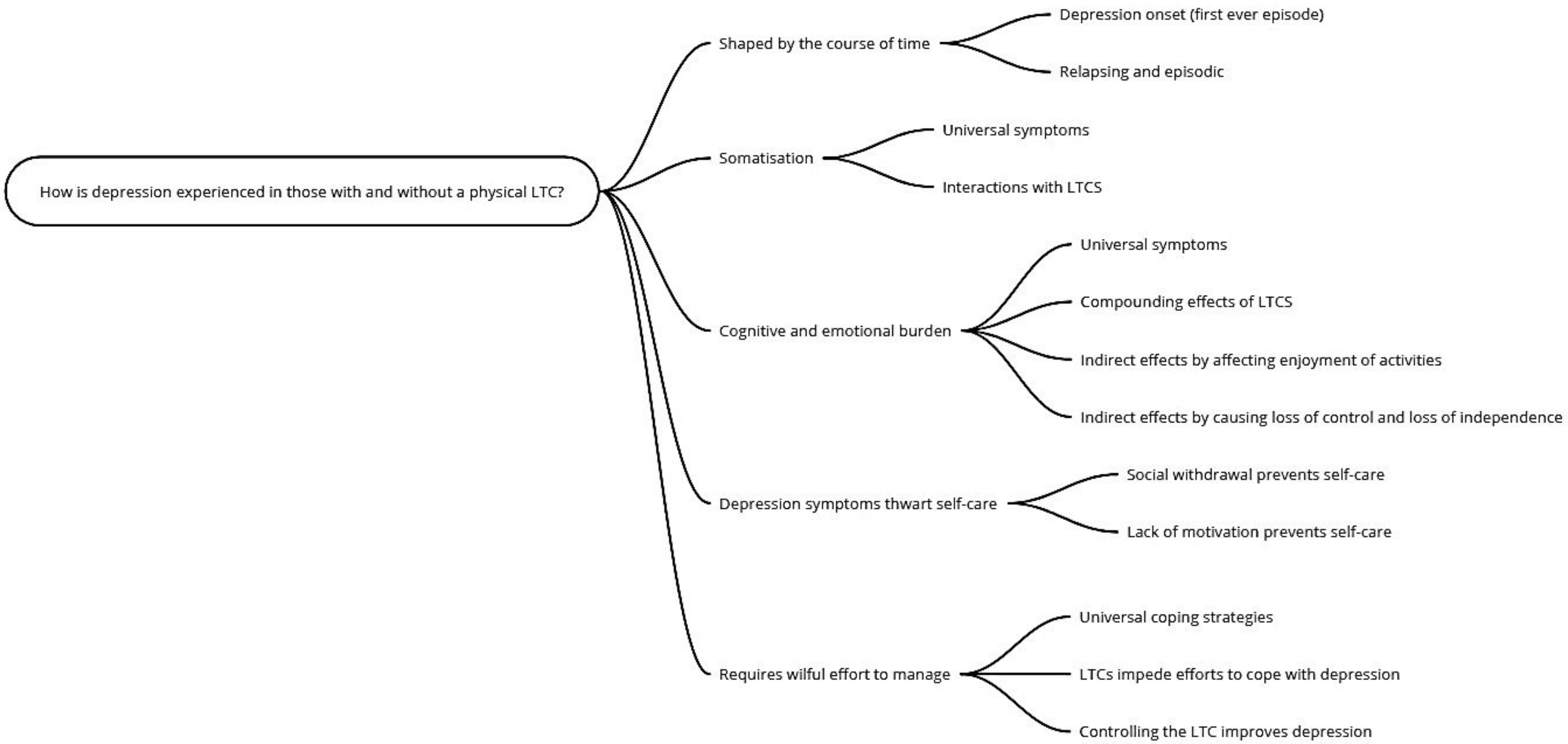

Figure 1. Schematic diagram of theme structure.

N.B. LTC: Long term condition.

For peer review only - http://bmjopen.bmj.com/site/about/guidelines.xhtml

Supplementary file: Appendix 1 - Vignettes

Scenario 1

Sarah has good days and bad, but on a bad day she feels so sad and miserable that she just can't stand it. It seems to completely take over her head like she's in a fog. She feels really disappointed in herself for feeling this way, despite all the good things she has in her life. Sometimes when she's by herself she cries and even has thoughts of killing herself just to try and be anywhere but here. She feels completely worthless.

Scenario 2

Most days Sam wakes up and feels so tired he wishes he could just stay in bed. In fact he feels so tired most of the time that he doesn't want to do anything. He sleeps longer than he used to and doesn't have the energy to do anything. Sometimes he doesn't even feel like eating. He's even lost weight without trying to. His partner tries to show him affection and wants a kiss and a cuddle, but he just feels numb and not interested at all.

Scenario 3

Jason finds it really hard to feel positive about the future at the moment, it's like he has nothing to look forward to anymore. He finds it difficult to concentrate and to make decisions these days too. He feels so frustrated and trapped by the way he feels. His friends tell him he should snap out of it, but he just can't. He wishes he didn't have to feel like this, it makes him feel like such a failure.

Scenario 4

Most days Clare feels like she is being punished for something she must have done in a past life. And then she feels guilty for thinking like this. She seems to worry about her health all the time and her aches and pains get on top of her. She doesn't enjoy things the way she used to, in fact she doesn't really get any pleasure out of anything anymore. She finds it hard to leave the house some days. Everything seems a bit pointless these days, if she's being completely honest.

Scenario 5

Susan has always enjoyed her food, but for the past couple of months she's noticed she's been putting on some extra weight. She finds she craves her favourite foods and is comfort eating to make herself feel a bit better. She's not sure she is feeling any better though, in fact she's sleeping really badly and often wakes up several times in the night. Her sleep is restless and she wakes up tired making each day feel like it drags.

For peer review only

For peer review only - http://bmjopen.bmj.com/site/about/guidelines.xhtml

LONDON'S GLOBAL UNIVERSITY

DEPARTMENT OF BEHAVIOURAL SCIENCE AND HEALTH

The Mood and Health Study

Interview Schedule – Depression

Introduction

Review of Participant Information Sheet

Consent form

Timing and confidentiality

My name is [insert name] and I am a researcher. I'm not a doctor or a psychologist and I don't have any clinical contact with your team. I'm interested in finding out from you what your experiences have been with your mood, and there are no right answers.

Vignettes

To start I'd like you to look at what some other people have experienced, and then we'll talk about them a bit. Firstly, I'd like you to start by sorting the cards into two piles, those which you think are relevant to you and those which aren't.

**Card sort**	Can you talk me through how you have sorted the cards?<br>Has anything like this ever happened to you?<br>Have you ever felt these sorts of things before?<br>How do you think this person's experience is different from yours?

Depression

**Precipitating events**	Take me back, can you tell me about when you first noticed changes to your mood?
Prompts	• Can you remember feeling differently?

For peer review only - http://bmjopen.bmj.com/site/about/guidelines.xhtml

	• How did it feel?<br>• Was there anything else you think may have contributed to your low mood?<br>• How do you think that experience relates to how you feel today?
**Diagnosis**	Can you talk me through the support you received during this time?
Prompts	• E.g. family, friends, GP?<br>• How did you find out you had depression? E.g. Who told you? When?<br>• How did you feel when you found out it was depression?
**Timeline**	How has your mood been since then?
Prompts	• How has you mood changed over time?
**Symptoms**	Can you tell me about a time when you felt your depression was at its worst?
Prompts	• How did it feel?<br>• How long did it last?<br>• Can you tell me how most days are for you at the moment?<br>• What's a good day for you?<br>• How long have you been feeling this way?<br>• Are there times when you don't feel this way? How long does this last?
**Management of depression**	Are you receiving any treatment for your depression at the moment?
Prompts	• Can you tell me about the care you have received for your depression from doctors?<br><br>e.g. who (GP, clinic nurse/doctor)<br>e.g. where (GP clinic, hospital clinic, community/third sector)<br>e.g. when (routine/booked appointments, length of appointments)<br>e.g. what (antidepressants, counselling)

For peer review only - http://bmjopen.bmj.com/site/about/guidelines.xhtml

	• Do you find it easy to talk to your doctor about your concerns?<br>• How did you find the treatment?<br>• How helpful has this treatment been? E.g. what was most helpful and least helpful?
**Self-care**	• Can you talk me through anything you do yourself that helps you cope better?
Prompts	• For example, exercise, eating habits, alcohol consumption, smoking, acceptance, distractions/hobbies, wallowing. Have any of these been helpful to you?<br>• How do these things make you feel?

Wrapping up

Is there anything else you would like to tell me?

How have you found this interview?

Do you have any questions?

Thank you for your time.

Give sources of further support handout.

Give baseline questionnaire.

Provide participant with £10 retail voucher.

For peer review only - http://bmjopen.bmj.com/site/about/guidelines.xhtml

The Mood and Health Study

Interview Schedule – Depression and Physical Illness

Introduction

Review of Participant Information Sheet

Consent form

Timing and confidentiality

My name is [insert name] and I am a researcher. I'm not a doctor or a psychologist and I don't have any clinical contact with your team. I'm interested in finding out from you what your experiences have been with your mood and health, and there are no right answers.

Vignettes

To start I'd like you to look at what some other people have experienced, and then we'll talk about them a bit. Firstly, I'd like you to start by sorting the cards into two piles, those which you think are relevant to you and those which aren't.

**Card sort**	Can you talk me through how you have sorted the cards?<br>Has anything like this ever happened to you?<br>Have you ever felt these sorts of things before?<br>How do you think this person's experience is different from yours?

Depression

**Precipitating events**	Take me back, can you tell me about when you first noticed changes to your mood?
Prompts	• Can you remember feeling differently?<br>• How did you feel?

For peer review only - http://bmjopen.bmj.com/site/about/guidelines.xhtml

	• Was there anything else you think may have contributed to your low mood?<br>• How do you think that experience relates to how you feel today?
**Diagnosis**	Can you talk me through the support you received during this time?
Prompts	• E.g. family, friends, GP?<br>• How did you find out you had depression? E.g. Who told you? When?<br>• How did you feel when you found out it was depression?
**Timeline**	How has your mood been since then?
Prompts	• How has you mood changed over time?
**Diagnosis of physical illness (if not covered above)**	Can you tell me how you found out about your [arthritis/heart disease/diabetes]?
Prompts	• How long ago was this?<br>• Who told you? Where were you?<br>• How did you feel when you found out?<br>• Did you find out about your [arthritis/heart disease/diabetes] before or after you found out you had depression? How did you find this time?
**Symptoms**	Can you tell me about a time when you felt your depression was at its worst?
Prompts	• How did it feel?<br>• How long did it last?<br>• Can you tell me how most days are for you at the moment?<br>• What's a good day for you?<br>• How long have you been feeling this way?<br>• Are there times when you don't feel this way? How long does this last?

For peer review only - http://bmjopen.bmj.com/site/about/guidelines.xhtml

**Interaction**	Can you tell me about how your [arthritis/heart disease/diabetes] affects your life now?
	• How does your [arthritis/heart disease/diabetes] make you feel?<br>• How do your depression and [arthritis/heart disease/diabetes] affect each other?<br>• Have you ever found it difficult to cope with [arthritis/heart disease/diabetes]?<br>e.g. coping with the symptoms, treatments?
**Management**	Are you receiving any treatment for your depression at the moment?
Prompts	• Can you tell me about the care you have received for your depression from doctors?<br>e.g. who (GP, clinic nurse/doctor)<br>e.g. where (GP clinic, hospital clinic, community/third sector)<br>e.g. when (routine/booked appointments, length of appointments)<br>e.g. what (antidepressants, counselling)<br>• How did you find the treatment?<br>• How helpful has this treatment been? E.g. what was most helpful and least helpful?<br>• How easy have you found it to get help for your depression?
	What treatments have you had for your [arthritis/heart disease/diabetes]?
Prompts	e.g. who (GP, clinic nurse/doctor)<br>e.g. where (GP clinic, hospital clinic)<br>e.g. when (more than one treatment?)<br>e.g. what (self-management courses, medication, surgery?)<br>• Do you find it easy to talk to your doctor about your concerns?<br>• How did you find the treatment?

For peer review only - http://bmjopen.bmj.com/site/about/guidelines.xhtml

	• How helpful has this treatment been? E.g. what was most helpful and least helpful?<br>• How do you feel treatment of your [arthritis/heart disease/diabetes] has affected your depression?<br>• **[Arthritis and angina only]** How have the treatments helped with your pain?
**Self-care**	• Can you talk me through anything you do yourself that helps you cope better?
Prompts	• For example, exercise, eating habits, alcohol consumption, smoking, acceptance, distractions/hobbies, wallowing. Have any of these been helpful to you?<br>• How do these things make you feel?

Wrapping up

Is there anything else you would like to tell me?

How have you found this interview?

Do you have any questions?

Thank you for your time.

Give sources of further support handout.

Give baseline questionnaire.

Provide participant with £10 retail voucher.

For peer review only

BMJ Open

The experience of depression in older adults with and without a physical long-term condition: findings from a qualitative interview study.

Journal:	*BMJ Open*
Manuscript ID	bmjopen-2021-056566.R1
Article Type:	Original research
Date Submitted by the Author:	13-Jan-2022
Complete List of Authors:	Poole, Lydia; UCL, Institute of Health Informatics<br>Frost, Rachael; University College London Research Department of Primary Care and Population Health, Research Department of Primary Care and Population Health<br>Rowlands, Hannah; King's College London<br>Black, Georgia; University College London, Applied Health Research; Dr
**Primary Subject Heading**:	Mental health
Secondary Subject Heading:	Qualitative research, Health policy
Keywords:	Depression & mood disorders < PSYCHIATRY, QUALITATIVE RESEARCH, Coronary heart disease < CARDIOLOGY, DIABETES & ENDOCRINOLOGY, RHEUMATOLOGY

SCHOLARONE™
Manuscripts

I, the Submitting Author has the right to grant and does grant on behalf of all authors of the Work (as defined in the below author licence), an exclusive licence and/or a non-exclusive licence for contributions from authors who are: i) UK Crown employees; ii) where BMJ has agreed a CC-BY licence shall apply, and/or iii) in accordance with the terms applicable for US Federal Government officers or employees acting as part of their official duties; on a worldwide, perpetual, irrevocable, royalty-free basis to BMJ Publishing Group Ltd ("BMJ") its licensees and where the relevant Journal is co-owned by BMJ to the co-owners of the Journal, to publish the Work in this journal and any other BMJ products and to exploit all rights, as set out in our licence.

The Submitting Author accepts and understands that any supply made under these terms is made by BMJ to the Submitting Author unless you are acting as an employee on behalf of your employer or a postgraduate student of an affiliated institution which is paying any applicable article publishing charge ("APC") for Open Access articles. Where the Submitting Author wishes to make the Work available on an Open Access basis (and intends to pay the relevant APC), the terms of reuse of such Open Access shall be governed by a Creative Commons licence – details of these licences and which Creative Commons licence will apply to this Work are set out in our licence referred to above.

Other than as permitted in any relevant BMJ Author's Self Archiving Policies, I confirm this Work has not been accepted for publication elsewhere, is not being considered for publication elsewhere and does not duplicate material already published. I confirm all authors consent to publication of this Work and authorise the granting of this licence.

For peer review only

The experience of depression in older adults with and without a physical long-term condition: findings from a qualitative interview study.

Lydia Poole[1], Rachael Frost[2], Hannah Rowlands[3], Georgia Black[4].

[1] Institute of Health Informatics, University College London, 222 Euston Road, London, NW1 2DA.

[2] Research Department of Primary Care and Population Health, Upper Third Floor, UCL Medical School (Royal Free Campus), Rowland Hill Street, London, NW3 2PF.

[3] Department of Psychological Medicine, Institute of Psychiatry, Psychology & Neuroscience, Kings College London, E3.18, De Crespigny Park, London, SE5 8AF.

[4] Department of Applied Health Research, University College London, 1-19 Torrington Place, London, WC1E 6BT.

Funding: LP and HR were funded by the UK Economic and Social Research Council (ES/N001478/1) for this project.

Declarations of interest: none.

Word count: 8119

Correspondence to:

Dr Lydia Poole

Institute of Health Informatics

222 Euston Road

University College London

London

NW1 2DA

T: +44 (0)20 3108 8817

E: lydia.poole@ucl.ac.uk

For peer review only - http://bmjopen.bmj.com/site/about/guidelines.xhtml

Abstract

Objective: To understand how the lived experience of depression differs among patients with a long-term condition (LTC) compared to those without an LTC, and how the experience differs across different types of LTC.

Design: Face-to-face, semi-structured, interviews.

Setting: Primary care; GP surgeries in and around North London.

Participants: 41 primary care patients with depression were recruited. Our sample comprised participants aged 55-75 years old with depression only (n= 12), depression and coronary heart disease (n=5), depression and type 2 diabetes (n=10) and depression and arthritis (n=14).

Results: Interviews were conducted, audio-recorded, transcribed, and analysed using thematic analysis. The results revealed that the cardinal diagnostic symptoms of depression (anhedonia, sadness) were experienced by all our participants regardless of LTC. However, the LTC did interact with depression by compounding somatic, cognitive and emotional symptoms, increasing disability and reducing independence, and hindering attempts at coping with mental illness. Our findings demonstrate common experiences across patients, as well as key differences based on LTC.

Conclusions: We suggest four key implications for future care practices of these patients: (1) Not all participants with depression and LTC view their mental and physical health as interconnected; there should be allowances in care plans for separate treatment pathways; (2) Key features of depression that affect LTC management are social withdrawal and lack of motivation to self-manage or access healthcare; (3) Key features of LTCs that worsen depression are pain, the unpredictability of future health and progressive disability; (4) Positive self-management of LTC could improve self-efficacy and therefore mood, and should be encouraged.

For peer review only - http://bmjopen.bmj.com/site/about/guidelines.xhtml

Key words: Depression; long-term conditions; co-morbidity; qualitative research; interview study; thematic analysis.

Strengths and limitations of the study

- Participant diagnoses derived from clinical records and confirmed by self-report.
- Participants were drawn from across multiple sites across North London, with diversity in demography achieved.
- Comparative thematic analysis allowing for comparisons within and between groups.
- Small sample of depression and coronary heart disease cohort.
- Majority female in the depression and arthritis cohort.

For peer review only

Introduction

Estimates from the UK Biobank study suggest the prevalence for a single episode of major depressive disorder is 6.4%, varying to 12.2% for moderate recurrent major depression and 7.2% for severe recurrent major depression,[1] among British adults. Depression has been associated with increased all-cause mortality;[2] however causality has not yet been determined.[3] One possible explanation is that individuals with depression are more than twice as likely to have a concomitant physical long-term condition (LTC), such as diabetes and coronary heart disease.

It has been hypothesised from quantitative investigations of epidemiological and clinical cohort studies that depressive symptoms which occur in cardiac patients are distinct from those experienced in psychiatric populations based on three key differences: composition of symptoms, timeline, and treatment responsiveness.[4] With regard to symptoms, the hallmarks of psychiatric depression are persistent sadness and loss of interest and pleasure in activities (anhedonia). These are often associated with cognitive changes such as a sense of worthlessness and guilt, among others. By contrast, there is growing recognition that depressive symptoms in people with physical illness are dominated by somatic symptoms, such as fatigue and loss of energy.[5,6] In addition, it has been suggested that the chronicity, and the timing of onset of depressive symptoms in relation to the LTC, have implications for morbidity and mortality.[7,8] Finally, it appears that different treatment strategies may be appropriate for those living with psychiatric depression compared to those who have comorbid depression,[9] with particular benefit coming from integrative collaborative care models of healthcare.[10] Qualitative data has revealed that depression in physical illness has been shown to have been normalised by health care practitioners leading to barriers in its management [11] and there may be resistance to antidepressant medication among some LTC patients.[12] Given these empirical observations surrounding the differences in depression symptoms, timeline and treatment, some authors have suggested that a separate subtype of depression may exist, driven by biological (inflammatory) changes [13] which in turn are implicated in LTCs.[14] However, despite a plausible hypothesis for the existence of a separate subtype of depression in those with an LTC, whether or not this translates to differences in patient experience

For peer review only - http://bmjopen.bmj.com/site/about/guidelines.xhtml

is unclear. Therefore, further qualitative research is warranted to examine whether the lived experience of depression is similar or different in patients with depression and/or a comorbid LTC.

Furthermore, experiences of depression across LTCs are likely to differ. A meta-synthesis of qualitative data has suggested that patients with depression and a comorbid LTC either see their mental and physical health as either separate and independent from each other, or interrelated with one having caused or worsened the other.[15] However, the similarities and/or differences in how depression is experienced in different LTCs is currently not well-understood. For example, depression in diabetes has been revealed to be heterogenous with aspects of diabetes-related distress and demoralisation being apparent,[16] while depression in CHD has been associated with loss, both in terms of interpersonal loss as well as loss of health/control.[12]

Therefore, the aim of this study was twofold. First, we aimed to examine how the lived experience of depression is similar or different between those with an LTC and those without, and second, we aimed to understand how experiences of depression are similar or different between types of LTC. In order to take into account the correlation between age and LTC onset, we focussed on the experiences of depression in adults in mid-late life.

Methods

Participant selection and recruitment

Thirty-seven participants were recruited using mailouts from 14 UK general practices in the Barnet and Enfield regions of North London; a further one participant was recruited using opportunistic sampling from GP surgeries in North West London; and a final three participants were recruited from the Camden and Islington IAPT (Improving Access to Psychological Therapy) service.

Our inclusion criteria led us to recruit participants who identified as male and female, aged 55-75 years old and English speaking. All participants were screened for current depression by participating GP practices via electronic and hand searches of medical records. Current depression was defined as a patient who had either received a clinical code of depression by their GP and/or had received a referral or prescription for

For peer review only - http://bmjopen.bmj.com/site/about/guidelines.xhtml

depression treatment in the preceding two months. All participants were medically stable outpatients living within the community. We excluded patients with a severe cognitive, mental or psychological impairment that would impede their ability to provide informed consent. Moreover, to compare patients living with depression, with and without an LTC, we recruited patients to one of four participant groups according to additional diagnoses identified in their medical notes: depression only, depression and CHD, depression and T2D, and depression and arthritis. To reduce cross-over between the groups, participants with more than one LTC of interest (i.e. diabetes and arthritis) in their medical notes were excluded. We also excluded those with another LTC such as cancer, stroke and chronic lung disease to allow us to focus our discussions more easily on our chosen LTCs. The LTCs were selected based on *a priori* knowledge to elicit a variety of illness experiences: coronary heart disease can have an acute, life-threatening presentation (e.g. acute coronary syndromes); T2D involves monitoring and self-management of blood sugar levels and can lead to acute episodes of illness (hypoglycaemia); arthritis is associated with pain and functional impairment.

Practice GPs reviewed the list of those to approach prior to mailout and removed people that met the exclusion criteria or they considered inappropriate to contact. The mail-out included full details of the study and interested patients were invited to contact the research team to discuss participation. Patients were screened by researchers over the telephone using the same inclusion and exclusion criteria before arranging a convenient time and place for the interview to take place. We used purposive sampling to capture a range of clinical and demographic characteristics within each group where possible; specifically, among those patients who expressed an interest in taking part we prioritised telephone screening to allow us to recruit a diverse sample by sex, age, ethnicity and location.

Interview data collection

We conducted 1:1 semi-structured face-to-face interviews, mainly at participants' homes or in a meeting room at UCL (or another convenient location if requested), lasting approximately one hour (range 42–97 minutes). Interviews were carried out between May to October 2019. Data collection continued within this time frame and ceased once all eligible patients at participating sites had been contacted. Interviews were conducted by

For peer review only - http://bmjopen.bmj.com/site/about/guidelines.xhtml

two female researchers, HR (MSc; research assistant) (n = 40) or LP (PhD; senior research fellow) (n = 1). The interviewers had received training in qualitative interviewing, and HR had prior experience of conducting qualitative interviews with vulnerable groups. Neither HR or LP had any prior connection with participants, other than the contact made during the recruitment process where the study objectives and procedures were explained. The interviews began by presenting participants with four short vignettes for participants to read and reflect on. The vignettes contained the experiences of fictional characters written in the third person (see Appendix 1) and were designed to encourage participants to reflect on their own symptoms of depression. This method is well-established for eliciting detailed information on sensitive topics in qualitative research.[17] The interview then covered a number of topics, to explore the experience of living with depression: diagnosis, precipitating events, symptoms, change in experience over time, management of depression and treatment preferences. In participants with a comorbid LTC we also explored the impact of depression on their physical illness and vice versa. The topic guides (see Appendix 2) were modified as interviews progressed, through team discussions. To circumvent issues surrounding stigma, initial questions steered clear of clinical labels, and instead invited participants to reflect on 'changes to their mood'. As each interview progressed the interviewer used the participants' own terminology. After each interview, demographic data and self-reported medical information were collected. The interviewer kept a reflective diary in order to document thoughts, feelings and other interesting contextual information that occurred to her. Participants were provided with relevant local and national mental health service information and a £10 retail voucher honorarium. Any concerns over participant safety (including suicidal ideation and self-harm) were discussed with the senior researcher (LP) before contacting participants' GP with their consent. A Standard Operating Procedure was in place for this. Interviews were audio-recorded, with brief field notes, transcribed verbatim by an external company and anonymised and verified for accuracy by the research team (HR and LP). Data for this study are archived in a public data repository.[18]

Patient and Public Involvement

For peer review only - http://bmjopen.bmj.com/site/about/guidelines.xhtml

Patients and public were not involved in the development of the research question, design or study implementation. However, the results of this study were disseminated via a large outreach and engagement event through social media and podcasting platforms.

Data analysis

We used reflexive thematic analysis [19,20] to analyse our data from a realist/essentialist perspective in which a (mostly) one-way relationship is taken to operate between meaning, experience and language. According to this approach, data saturation is not required, rather meaning is conceived as emerging from the interpretation of the data.[21] All transcripts contained the entire interview, including the discussion that took place regarding the material in the vignettes. The data arising from the discussion of vignettes varied across participants; some participants did not engage with the vignettes and the conversation quickly flowed to the semi-structured interview questions, whereas for others it prompted a divulgement of prior personal events. As such, this data was not separated, but rather the entire transcript was treated as a single data piece. Transcripts were read by HR (a health psychology researcher expertise in LTCs) and LP (a health psychology researcher with an interest in depression and multimorbidity); GB (a health services researcher with expertise in health improvement) and RF (a health services researcher with expertise in ageing and mental health) read a subsample. Data analysis was led by LP. Transcripts from each group were first read and analysed inductively, separately, to enable us to build an understanding of the idiographic qualities of the dataset;[19] inductive analysis is a bottom-up approach where the codes and themes are driven by the data and not by a theoretical framework. Each set of transcripts was read repeatedly, and codes were developed by LP in conjunction with HR. HR's reflective diary was consulted to ensure no additional meaning or interpretation could be gleaned from the data. Discrepancies were discussed until consensus was reached. Coding of transcripts was performed using NVivo 12 (QSR International Pty Ltd., 2018). A codebook was kept to organise ideas and for note keeping. For example, our coding tree included codes for the onset and timeline of depressive symptoms, stressful life events, cognitive symptoms of depression, somatic symptoms of depression, past decisions, social connections, emotional response to illness, physical illness impacting daily living, the symptoms of the physical

For peer review only - http://bmjopen.bmj.com/site/about/guidelines.xhtml

illness, approaching death, coping strategies and help-seeking and attitudes towards care. Codes were then grouped to construct themes; refined through team discussions and piloting. After conducting the initial analysis across the entire dataset, we tabulated the themes and subthemes using Excel (Microsoft for Windows 365) to allow us to systematically explore the similarities and differences in experiences across groups. This approach was informed by Framework Analysis [22] and has been combined with thematic analysis by previous researchers conducting comparative qualitative analysis.[23] The indexing and charting of the data enabled us to maintain rigour in the analytic process. Themes were then written out and refined with feedback from team. The study was approved by NHS London – Fulham Research Ethics Committee (reference: 18/LO/2150).

Results

Participants

Out of 137 patients who expressed an interest in the study, 45 were eligible and 41 completed interviews. Reasons for ineligibility included not meeting the clinical inclusion criteria (n = 37), non-responsive to return phone calls (n = 26), not interested (n = 3), unavailable for interview (n =1), sample size deemed sufficient for the cohort (n = 23), non-English speaker (n = 2). Participants were grouped into 4 cohorts based on their medical records and verified during screening telephone calls: depression only (n = 12), depression and CHD (n = 5) and depression and type 2 diabetes (T2D) (n = 10) and depression and arthritis (n = 14). In terms of CHD, patients in this group had received a diagnosis for myocardial infarction (n = 1), angina (n=2) and heart failure (n = 2). In terms of arthritis, patients in this group included those with rheumatoid arthritis (n = 2) and osteoarthritis (n = 12). Overall, participants were aged 63.5 years (standard deviation (SD) 5.5) and we sampled roughly equal numbers of men and women. However, the majority of depression and arthritis participants were female (93%). Approximately one-third of the sample were from a non-White ethnic group and 85.4% of all participants were taking antidepressant medication(s) at the time of the interview. The characteristics of the sample are presented in Table 1.

For peer review only - http://bmjopen.bmj.com/site/about/guidelines.xhtml

Thematic overview

We generated five overarching themes that encompass the experience of living with depression in those with and without an LTC. In each theme we have drawn out the relationship between the experience of depression in those with and without an LTC, and where relevant, compared this across the different LTC groups (CHD, T2D and arthritis). Across all the themes, we found that the experience of depression and LTCs acted to compound the effects of each other; this was particularly apparent for those whose illness had resulted in changes to daily living. Figure 1 presents a schematic diagram of our themes.

Theme 1: Shaped by the course of time

Across our sample, depression was not static; it was shaped by individuals' life histories and past events. The experience of depression also shifted over time so that as people's lives progressed, different triggers for new episodes became more apparent. In those with an LTC, hardship brought about by their LTC and a loss of health was described by some as a trigger for depression.

Depression onset (first-ever episode)

In participants with and without LTCs, the onset of the first-ever episode of depression occurred at different ages; many could identify roots for their depression in childhood or adolescence, while others had received a diagnosis more recently. Across the groups, most participants were able to identify a precipitating adverse life event(s) to their first-ever depressive episode such as financial insecurity, (multiple) bereavement, a relationship breakdown, children taken into care, and physical and sexual abuse. For some, seemingly smaller grievances, had accumulated over time to initiate the first-ever episode of depression.

In contrast to the depression-only participants, in participants with an LTC, life histories revealed two separate discourses relating to the onset of the initial depression diagnosis: coincidental and interconnected. As such, for some, the LTC was coincidental to the onset of the depression, with the depression and LTC diagnoses occurring at different times and in relation to different precipitating/surrounding events. This discourse was predominately discussed by those with T2D, and to a lesser extent the CHD cohort. These individuals had a

For peer review only - http://bmjopen.bmj.com/site/about/guidelines.xhtml

clear narrative about the onset of their depression, that was related to other life pressures such as caring responsibilities, fraud allegations and work rather than to their LTC:

> "I don't think so. I don't really think so. I had no qualms about my health. It was the other things. The inferiority complex, the pressure of work and the unhappiness with my married life. It was those things." **(P.39, depression and T2D, male)**.

> "I honestly can't say that [depression] is the reason of that [CHD]. But yes, when you have I suppose chest pain you think about it, you sit down, you [pause]. But I don't think it took me into depression at that time or anxiety or something." **(P.45, depression and CHD, male)**.

The second discourse highlighted the interconnected nature of the initial depression and LTC diagnosis. For these participants, depression was a new experience, and was directly attributed to their LTC, or its implications (e.g. hospitalisations, loss of mobility, job loss). The type of LTC did not make a difference, only whether the LTC was associated with unpredictable, major, and irreversible upheaval.

> "Depressed? It was when I came out of hospital after having my triple bypass because my life changed completely. I lost my job, my relationship broke up, the kids and all that and I just sort of had a sort of breakdown and that was it really." **(P.44, depression and CHD, male)**.

Relapsing and episodic

Across all participants, after the initial onset, the experience of depression had a varying time course. A few participants had new onset depression. However, for the majority of participants, their history of depression was chronic, with repeated episodes over time. This had led to a familiarity with when a new 'bout' was coming on, even if there was no discernible trigger.

Where there was a clear trigger for a new episode of depression, this was often a difficult life event, such as a child going to prison or a transition to retirement. However, for some people with chronic, pre-existing depression, the LTC had also triggered a new episode. This led to a new experience of depression for these participants, which was not centred around their earlier difficult life circumstances which had originally

For peer review only - http://bmjopen.bmj.com/site/about/guidelines.xhtml

brought about their depression. Instead, the LTC raised fears surrounding declining health, death and frailty, which fed into the new depressive episode:

> "The depression since than that was different. It was felt trapped then … I was full of tension, full of uncomfortable feeling with him. I just couldn't, it was like having a snake you know circling around you know... So that is thank God I don't have that. The depression that I feel in this and that is because of my health problems and that bothers me, that bothers me a lot." **(P.22, depression and CHD, female)**.

Theme 2: Somatisation

Somatic symptoms of depression were frequently experienced by our participants, in those with and without an LTC. The experience was largely similar across our four cohorts. Differences did exist in sleep disturbance and crying, with LTCs (mainly arthritis but also T2D) contributing to the experience of these somatic symptoms.

Universal somatic symptoms

Somatic symptoms were discussed by all our participants, irrespective of LTC. Symptoms discussed included appetite changes (including over- and under-eating), crying, and loss of intimacy. By far the most common of all the somatic complaints was sleep disturbance. Some described difficulties falling asleep, restless and disturbed sleep, vivid dreams and nightmares, and difficulty getting out of bed in the morning. Psychomotor retardation was another common somatic complaint linked to fatigue, which was experienced across all groups, irrespective of LTC.

> "Really, really very slow, very sluggish. Very physically, very physical. In fact, almost as physical as it is mental…. Yeah like you're swimming. Like you're swimming against a sea of something coming at you. And you're trying to wade yourself through and knowing that if you, and no energy, zero physical energy." **(P.13, depression only, female)**

Somatic symptoms and interactions with LTCs

For peer review only - http://bmjopen.bmj.com/site/about/guidelines.xhtml

For those with an LTC, the illness also directly contributed to the experience of somatic symptoms of depression. Sleep was negatively affected by the LTC through worries about managing the condition (as with P.2 with T2D) or pain. Pain was particularly associated with participants in the depression and arthritis cohort.

> "I haven't been a very good sleeper lately; I don't sleep the full 8 hours that they say you should do. I do think about things and one of the things obviously I do think about is make sure my diabetes is correct." **(P.2, depression and T2D, male)**.

> "I can't sleep at night because of pain… And even at night I get a lot of pain in my back and that so I am just putting my legs round 10 times this way and 10 times that way because I can't sleep." **(P.4, depression and arthritis, female)**.

While crying was frequently described across all our participants, in our arthritis participants we observed a unique contribution of the LTC. Arthritis (and in particular arthritic pain) compounded the emotional and somatic experience of depression, and could prompt tears of fear and frustration.

> "I am chopping up the onions, I have to go and sit down because the pain in my back is such that I can't, I have to sit down. And like I have cried with it, not just from the onions but real tears, through frustration I suppose and pain." **(P.41, depression and arthritis, female)**.

Theme 3: Cognitive and emotional burden

In this theme the participants' lived experience of the cognitive and emotional symptoms of depression are described. These key diagnostic symptoms of depression were clearly experienced by all our participants. However, we observed compounding and indirect routes linking LTCs with the cognitive and emotional burden of depression; moreover, thoughts of death were particularly prominent within the depression and CHD group.

Universal cognitive and emotional symptoms

For peer review only - http://bmjopen.bmj.com/site/about/guidelines.xhtml

Participants, regardless of LTC, had an overwhelming sense of disinterest in the world around them; they described a deep-rooted sadness, hopelessness, worthlessness and low self-esteem. Some felt as though they were being punished.

> "You get what you deserve in life. And I don't deserve nothing. You know, I wasn't a good father when I was young. I was selfish. And it's come back and bit me up the bum, bit me on the bum. ..." **(P.12, depression only, male).**

Compounding effects of LTC on cognitive and emotional depressive symptoms

For some, the combination of the LTC and the depression led to a compounding effect on the cognitive and emotional depressive symptoms. In this way, the LTC directly contributed to the participants' experience of depression. This was particularly observed in those with T2D and arthritis.

> "I'm sitting there and taking the blood and giving myself injections and some days I just think, you know, how come it's come to this? And I know how it's come to this because, you know, I'm not managing my life in any sensible way, and I know that." **(P.25, depression and T2D, male).**

For participants with severe arthritic pain, pain was seen as an all-consuming and debilitating experience, which led to misery.

> "I'm getting pain back in my right knee and I'm going to have to eventually have that one done. It's really, oh god, what does that feel like? When I'm not feeling … oh ... it feels like having been almost injected with negativity." **(P.28, depression and arthritis, female).**

For P.32 her depression and the functional limitations imposed by her arthritis were difficult to unravel, with both acting in synergy to lead to a sense of hopelessness:

> "I'm also physically unable to do as much as I would like to do in terms of just - housework and shopping and it's difficult to pull it apart which is leading the sort of feelings of hopelessness and you know is it worth it and I can't, I might as well just stay in bed." **(P.32, depression and arthritis, female).**

Indirect effects of LTC upon cognitive and emotional depressive symptoms through affecting enjoyment of activities

For peer review only - http://bmjopen.bmj.com/site/about/guidelines.xhtml

For some of our LTC participants, the depression was a greater burden than their LTC, so cognitive and emotional depressive symptoms were experienced regardless of their LTC. In this way, some of our LTC participants experienced feelings of anhedonia, worthlessness, guilt, sadness, etc., similarly to the depression only participants. However, for others, the LTC (notably T2D and arthritis) fed into the experience of cognitive and emotional symptoms by removing their enjoyment of previously pleasurable activities.

An example comes from P.2, whose diabetes directly contributed to his feelings of anhedonia. This participant was particularly concerned about managing his blood glucose levels during social situations.

> "You are part of it because you are invited, you all go out, you are all friends, you have known one another for years and everything is there, but you don't feel as if you are really taking part if you get what I am saying. So that's the part of the mood I have found since I have been diagnosed with diabetes and that's the thing that holds me back from properly being 100% committed to enjoying it." **(P.2, depression and T2D, male).**

Pain also featured heavily in the descriptions of altered lifestyles recounted by our arthritis participants, perpetuating feelings of anhedonia.

> "That's, and I do enjoy my food, I do enjoy cooking. I mean I haven't had a dinner party now for over a year. I have always enjoyed doing that. But that's, and it's like washing up, you know I can do so much, […] but you know like my washing up is in stages. I will do this first, then go and sit down, come back and do that lot, the next lot, then go and sit down. […]. That is where the arthritis and depression, they are like, they join forces." **(P.41, depression and arthritis, female).**

Indirect effects of LTC on cognitive and emotional symptoms by causing a loss of control and independence

Financial insecurity and unequal personal relationships were experienced by many of our participants, irrespective of LTC, and had contributed to feelings of being out of control. Deteriorating physical health (particularly for those with arthritis) and increasingly frailty, had also led to feelings of loss of control for some. This was linked to feelings of loss of independence, which in turn led to feelings of depression.

For peer review only - http://bmjopen.bmj.com/site/about/guidelines.xhtml

"Some of my clothes are high up, I can never reach them unless somebody is there, that makes me depressed having to rely on somebody all the time." **(P.4, depression and arthritis, female).**

Thoughts of dying

Suicidal ideation, thoughts of death (but not suicide) and actual suicide attempts were disclosed in participants with and without LTCs. Although the presence of an LTC did not influence suicidal thoughts, having CHD was an important leveller for these participants. These participants lived with the unpredictable nature of their illness and the threat of sudden and irreversible loss of health:

"Even now I still think about it because when I just took a deep breath here, I felt a bit of a twinge there and it's when I got told about it, it was very disturbing because I was shown the blockage […] and although I've been reassured that it won't go up, block my heart or anything like that, but it makes you wonder because you never know what might happen." **(P.30, depression and CHD, male).**

For P.44 his brush with death due to having a heart attack has given him a greater acceptance of death:

"Well I just didn't give a shit about anything anymore, you know, apparently according to them I died twice, so I didn't give a shit no more about it, death doesn't scare me no more." **(P.44, depression and T2D, male).**

For others with CHD, they were also having to confront death, but they discussed their fear surrounding a prolonged or sudden death:

"I keep saying to my sister, 'I don't care if I die actually in my sleep that will be fine. But if I don't die and I can't move, like I said to you if I can't move and I can't get up and can't call that horrifies me.' That is enough to, well you know." **(P.22, depression and CHD, female).**

"So, since that day, perhaps I have, it wasn't so much when I had the heart bypass, but since the [aortic] aneurism and everything else, […] I worry that every time I go to bed, I don't know if I'm going to wake up. You know. So, I worry about that." **(P.30, depression and CHD, male).**

Theme 4: Depression symptoms thwart self-care

For peer review only - http://bmjopen.bmj.com/site/about/guidelines.xhtml

In this theme we draw on the ways in which the experience of depression impeded our participants' ability to take care of themselves; many described not washing or brushing their teeth, not wanting to cook proper meals, and retreating from their social network. This was apparent for all our participants but added an extra layer of complexity to participants with an LTC.

Depression leads to lack of motivation to self-care

Depression was associated with an apathy which led to a loss of interest in behaviours that might lead to improved self-care. This was true of behaviours that might improve the depression itself such as engaging in social activities and exercising, as well as those important for managing LTCs.

The impact of depression on attempts to control diabetes could lead to self-neglect, both in terms of medication adherence and adherence to medical appointments.

> "I suppose the most obvious impact is there are days when I just can't be bothered to take my medication, both the medication for the depression and the insulin for my diabetes and I know it's stupid […]." **(P.25, depression and T2D, male).**
>
> "there are times now when I wouldn't not turn up for an appointment, absolutely, I just […] But yes, there are times when I just think "what's the bloody point?" in dealing with daily stuff." **(P.25, depression and T2D, male).**

Exercise was seen as an important self-care behaviour for both mental and physical health among our participants. However, P.32 described how her lack of motivation to exercise was contributing to her physical decline, which in turn compounded her feelings of depression.

> "As for the physical side of things, again, I suspect they would like [me] to say, I'll lose weight, get more exercise and it's very difficult when you don't want to go out of the house, particularly I haven't the confidence to go into a gym or to a swimming pool, it's not really, you know. […] So, and the other fear is, of course, the older I get, the general, you know, aging and infirmities, you know, yeah, it

For peer review only - http://bmjopen.bmj.com/site/about/guidelines.xhtml

worries me greatly. You know, and it doesn't help the depression to lift. **(P.32, depression and arthritis, female).**

Social withdrawal prevents self-care behaviours

Many participants described how their attempts to cope had been thwarted by the depression itself. This was particularly true for those whose depression had led to social withdrawal. The retreat from daily life saw participants becoming withdrawn from both the outside world and their family and friends**.** Social withdrawal impacted on participants' management of their LTC. This was observed particularly in those with T2D and arthritis.

> "I did find it hard to leave the house sometimes, you know. You'd just sort of eat what was there rather than go shopping, you know." **(P.27, depression and T2D, female).**

> "Well they said because of the way, it's not helped, because I stopped, I wouldn't get out of the house, I was staying in my room, that can affect arthritis because then you are letting it seize up more." **(P.3, depression and arthritis, female).**

As seen with those experiencing a lack of motivation, social withdrawal also negatively impacted participants' willingness to engage with medical professionals (both for depression and/or the LTC).

> "I mean even the doctor, I have got to phone her tomorrow, they are going to arrange for the nurse to come out because I am overdue blood tests and blood pressure and that […]. Because she knows that I just don't want to leave the house again." **(P.3, depression and arthritis, female).**

Theme 5: Requires wilful effort to manage

The experience of depression was described as an active process, requiring an individual to find ways to cope. For some with an LTC, efforts to cope with their depression were thwarted by the functional limitation imposed by their illness. Controlling the LTC was also important to help manage the depression.

Universal coping strategies

For peer review only - http://bmjopen.bmj.com/site/about/guidelines.xhtml

While many of our participants struggled to cope, nearly all engaged in some strategies to navigate their current situation such as the use of alcohol and food, medical treatments, and drawing on friendships and family members for support. Often this included attempts to mask feelings as a means to find comfort; alcohol in particular was frequently described as a way to relax and forget.

> "But what I have done, and I have been guilty of, is purchasing alcohol and trying to blot it out with the alcohol." **(P.23, depression and arthritis, female).**

Many participants were engaged with healthcare professionals for depression management. For some anti-depressants had been beneficial, making their symptoms less severe. However, for others, side effects or fears surrounding dependency prevented their use.

> "Most of us are given medication, which makes you zombie, anyway. It doesn't help you. It just block your pains, you know. You are numb. You are not sorting out. And how long are you going to use those medication?" **(P.24, depression and T2D, female).**

LTCs impede efforts to cope with depression

Having an LTC prevented some participants from engaging with coping strategies to help manage their depression. For those with arthritis this included being unable to take part in activities that promote wellbeing, including hobbies.

> "But I think, yeah, so coping, well I am not walking as much as I want to, I have stopped going, I used to go to water workout, water aerobics every week and I have stopped doing that, partly because of the difficulty of getting into my bathers and afterwards drying myself." **(P.1, depression and arthritis, female).**

As P.32 described, the combination of her depression and arthritis makes coping with each insurmountable:

> "You know so the depression makes you lack motivation to, perhaps, I say modify some of the effects of the arthritis, but the arthritis also, in limiting you, makes the depression seem worse. Together it seems like a huge, insurmountable problem because if, on their own, they might be manageable, […]. So it is, yes, it impedes any kind of self-care or therapy, if you like, together."

For peer review only - http://bmjopen.bmj.com/site/about/guidelines.xhtml

Controlling the LTC improves depression

Interestingly across all LTC groups, those who successfully managed to engage with strategies to cope with their illness, saw benefits to their mental health through feeling an increased sense of control/self-efficacy. For example, failure to self-manage insulin dosages appropriately negatively impacted P.25's mood, while being in control of his T2D also helped him self-manage his depression.

> "But I carry glucose around with me. … I think we were away somewhere and, but I was completely useless for about two hours and I suppose I, you know, I had a moment of clarity about the need to keep on top of it properly, because I sort of knew that if I didn't, not only would I feel crap, physically, the idea of feeling crap physically was depressing." **(P.25, depression and T2D, male).**

Interestingly, P.30 with CHD found the unpredictable nature of his cardiac symptoms very distressing, but the use of his own blood pressure monitor helped him regain a sense of control.

> "this is my life saver, my BP [blood pressure] machine, I do it every day, […] I was told 140 over 80, right, yes, 140, 80, so once it's within this, I go to sleep a bit better. If I didn't know what my BP was, I'd be worried even more." **(P.30, depression and CHD, male).**

For some participants the physical limitations imposed by their LTC led to social isolation. This was particularly apparent for those with arthritis. If participants received support with things that their arthritis prevented (for example housework, food shopping), this was seen as positive for managing their depression, allowing them to live well within the limits of the LTC.

> "So yeah, [lady friend's name] and [man friend's name] took me out yesterday to go to Aldi, it's the first time since February that I have gone out to the shops." **(P.3, depression and arthritis, female).**

Discussion

This paper explored the lived experience of depression in primary care patients with and without a comorbid physical LTC (CHD, T2D and arthritis). Using thematic analysis, we generated five overarching themes. 'Shaped by the course of time' revealed a shifting, non-linear experience of depression in which the experience was

For peer review only - http://bmjopen.bmj.com/site/about/guidelines.xhtml

defined by earlier life experiences as well as more minor, cumulative events. For some, the LTC diagnosis had caused catastrophic upheaval to participants' lives; for these individuals the LTC was capable of triggering depression (both first-ever or a recurrent episode). For many, other life events were more prominent to determining the course of depression; for these participants the LTC was coincidental, running alongside the depression but not triggering new episodes. 'Somatisation' highlighted the physical experience of depression in our participants; for many, these symptoms were as dominant as the cognitive and emotional symptoms. Somatic symptoms were universally experienced across all our groups, in particular feelings of tiredness and fatigue and changes to eating habits. Where LTCs did interact with the somatic symptoms, this was mainly driven by arthritic pain contributing to and compounding these symptoms. 'Cognitive and emotional burden' explored the impact of mental symptoms, such as feelings of worthlessness, guilt, punishment, anhedonia and sadness, on participants. LTCs compounded these cognitive and emotional experiences as well as indirectly contributing to a worsening of these symptoms by affecting participants' enjoyment of previously pleasurable activities and causing a loss of control and independence. Thoughts of dying were frequently experienced, and prominent in patients with CHD where the unpredictability of future health was a psychological challenge. Theme 4, 'Depression symptoms thwart self-care' examined how depression itself acts as a barrier to engagement in self-care behaviours. Loss of motivation and social withdrawal and were key aspects of the depressive experience which thwarted attempts for LTC self-management (particularly those with T2D). Finally, 'Requires wilful effort to manage' revealed universal engagement in many coping strategies, particularly attempts to mask depressive feelings such as the use of alcohol and food. However, LTCS (particularly arthritis) did impede participants' ability to cope with depression, while successful management of the LTC had a positive impact on mood.

Findings from quantitative studies continue to make attempts to understand the relationship between depression in LTCs, with the causal model thought to be bidirectional.[9] Our findings supported the notion that the LTC was capable of triggering depression; however, this was restricted to those whose LTC has caused major life upheaval (e.g. loss of employment, relationship breakdown). It has been estimated that around 80% of initial depressive episodes occur after a stressful life event;[24] and that the severity of stressors required

For peer review only - http://bmjopen.bmj.com/site/about/guidelines.xhtml

to precipitate recurrent depressive episodes decreases over time.[25] None of our participants described their depression acting as a precursor to their LTC. In fact, for many, the LTC was experienced as coincidental to the onset of depression; this is a view that has been supported by a previous qualitative meta-synthesis.[15]

The difference in the experience of depression in those with and without an LTC has not been studied previously using qualitative research. Quantitative research has identified differences in terms of symptom presentation, chronicity and timing of symptoms, and treatment responsivity.[4] Our findings have added to this by revealing that while the symptoms of depression (somatic, cognitive, affective) were universally experienced by those with and without depression, the LTC contributed to the burden of these symptoms by either directly provoking them (e.g. arthritic pain leading to tears of frustration) or by indirectly leading to negative mood by undermining the enjoyment of partaking in previously pleasurable activities as well as reducing individuals' sense of independence. T2D and arthritis were key in this regard since both illnesses were associated with lifestyle changes which negatively impacted the participants' depression, increasing feelings of anhedonia, sadness, guilt and worthlessness. Previous research has explored the impact of arthritis on leisure activities, finding that functional and physical constraints imposed by the illness can affect self-esteem.[26] Our findings extend this to the experience of those with arthritis *and* depression, suggesting that these limitations worsen the symptoms of depression. Participants with diabetes also saw a compounding effect of their physical condition on their mental health. This was particularly true for those who described being distressed by T2D and its management. Indeed, diabetes distress is now recognised as a separate disorder to major depression [27] which has been linked to feelings of burden, difficulties adhering to medication regimes and depression.[28]

Loss of control and lack of independence featured heavily in the accounts of our participants with arthritis; whereby functional limitations imposed by the LTC indirectly impacted depression by removing individual's sense of control and autonomy. Loss was a key theme identified in the qualitative UPBEAT-UK study of patients with depression and CHD, where patients described not only loss of health but also interpersonal loss.[12] Health deterioration featured heavily in the accounts of all our participants with a physical LTC, and

For peer review only - http://bmjopen.bmj.com/site/about/guidelines.xhtml

while thoughts of dying were universally experienced across groups, our CHD group revealed a strong sense of an unpredictable and uncontrollable death, which was not apparent in those with T2D and arthritis. This realistic fear underpinned the experience of depression in these participants.

Our findings also observed differences in the experience of depression in participants' ability to self-care and to cope with their mental health. Social withdrawal and lack of motivation were commonly experienced in all our participants. We described how these features of depression thwart attempts to engage with self-care behaviours such as personal hygiene, meal preparation etcetera, as well as LTC (particularly T2D) specific illness-management behaviours such as adhering to medication and attending hospital appointments. While depression has been associated with poor medication adherence in previous quantitative studies of T2D and CHD patients [29,30], there has been a lack of qualitative studies describing the hows and whys of this phenomenon; more research in this area is warranted.

Findings from our study revealed the adoption of common coping behaviours across all our participants such as using alcohol and food to mask symptoms and drawing on expert sources of support; this supports findings from earlier work.[12] However, importantly we observed that the presence of an LTC interacted with participants' attempts to manage their depression. Those with an LTC, and in particular those with increasing disability from their arthritis, were prevented from engaging with coping mechanisms they had previously adopted to promote mental wellbeing. On the upside, positive self-management of the LTC improved feelings of control and self-efficacy and in so doing improved depressed mood. Depressed patients have previously been reported to benefit most from self-efficacy gains from LTC self-management training;[31] the importance of this construct for therapeutic purposes within the modern care setting of depression and LTC patients, requires future investigation.

Our findings have a number of important implications for care models such as the NHS' IAPT-LTC programme.[32] Our findings suggest that any care model of depression in LTC needs to take into account four overarching conclusions based on our data: (1) Not all participants with depression and LTC view their mental and physical health as interconnected; therefore there should be allowances in care plans for separate

For peer review only - http://bmjopen.bmj.com/site/about/guidelines.xhtml

treatment (this is a view endorsed by Knowles and colleagues).[10] (2) Key features of depression that affect LTC management and need to be considered when planning treatment and referring to psychological services are social withdrawal and lack of motivation to self-manage or access healthcare. (3) Key features of LTCs that worsen depression that should be discussed during care planning are pain, the unpredictability of future health and progressive disability. (4) Positive self-management of LTC could improve feelings of control and self-efficacy and therefore mood, and should be encouraged.

A strength of our study was in the recruitment of participants from GP practices ensuring all our participants had received clinical diagnoses of their health conditions. To understand the differences in depression across different LTCs we excluded participants with more than one LTC of interest and with a serious or life-limiting diagnosis such as cancer, stroke or dementia. Due to the increased risk of physical illness with age, we recruited participants within an age bracket of 55 to 75 years; this enabled us to focus on the experiences of adults in mid to late life. The study was confined to North and North West London; however, we recruited from across contrasting areas to elicit a range of experiences. Despite our best efforts, unfortunately our arthritis sample was predominately female (90%); however this may partly reflect the greater incidence of osteoarthritis in women in the UK.[33] Our depression and CHD group was small (n=5); therefore our findings may not reflect the diversity of views that a larger sample might have attained. Also, as part of our reflexive practice, we noted that as researchers our experience and identities as female academic researchers working in London, of White (HR, RF, GB) and mixed (LP) ethnic background, is likely to have shaped our understanding, prioritisation, and interpretation of the data. The trustworthiness of our analysis, however, has been enhanced through researcher triangulation, drawing on the multiple expertise of the research team. Moreover, we used multiple levels to the analysis process from use of the reflective diary, through to the generation of themes, and the comparative exercise through the charting of codes. Participant checking was not undertaken which could have added a useful check to our interpretive work.

In conclusion, the results of our face-to-face interview study of depression in those with and without a physical LTC revealed that overall, key diagnostic symptoms of depression (anhedonia, sadness etc.) were experienced

For peer review only - http://bmjopen.bmj.com/site/about/guidelines.xhtml

by all our participants regardless of LTC. However, the LTC did interact with depression by compounding somatic, cognitive, and emotional symptoms, increasing disability and reducing independence, and hindering attempts at coping with mental illness. Our findings have implications for the development and fine-tuning of mental health and LTC care programmes.

Contributorship statement:

LP was responsible for funding acquisition and conceived the study design. GB and RF advised on the study methods. LP and HR collected the data. LP and RF led the data analysis. GB and HR contributed to the data analysis. LP wrote the initial manuscript draft. RF, GB and HR edited the manuscript.

Competing interests:

The authors have no competing interests to declare.

Funding:

LP and HR were funded by the UK Economic and Social Research Council (ES/N001478/1) for this project.

Data sharing statement:

The data for the study are archived in the UK Data Service.[18]

For peer review only

References

1 Smith DJ, Nicholl BI, Cullen B, *et al.* Prevalence and Characteristics of Probable Major Depression and Bipolar Disorder within UK Biobank: Cross-Sectional Study of 172,751 Participants. *PLOS ONE* 2013;**8**:e75362. doi:10.1371/journal.pone.0075362

2 Machado MO, Veronese N, Sanches M, *et al.* The association of depression and all-cause and cause-specific mortality: an umbrella review of systematic reviews and meta-analyses. *BMC Med* 2018;**16**:112. doi:10.1186/s12916-018-1101-z

3 Roest AM, de Jonge P. The heart of the matter: in search of causal effects of depression on somatic diseases. *BMC Med* 2018;**16**:147. doi:10.1186/s12916-018-1144-1

4 Poole L, Dickens C, Steptoe A. The puzzle of depression and acute coronary syndrome: reviewing the role of acute inflammation. *J Psychosom Res* 2011;**71**:61–8. doi:10.1016/j.jpsychores.2010.12.009

5 de Miranda Azevedo R, Roest AM, Hoen PW, *et al.* Cognitive/affective and somatic/affective symptoms of depression in patients with heart disease and their association with cardiovascular prognosis: a meta-analysis. *Psychol Med* 2014;**44**:2689–703. doi:10.1017/S0033291714000063

6 Poole L, Jackowska M. The association between depressive and sleep symptoms for predicting incident disease onset after 6-year follow-up: findings from the English Longitudinal Study of Ageing. *Psychol Med* 2018;:1–10. doi:10.1017/S0033291718001290

7 Dickens C, McGowan L, Percival C, *et al.* New onset depression following myocardial infarction predicts cardiac mortality. *Psychosom Med* 2008;**70**:450–5. doi:10.1097/PSY.0b013e31816a74de

8 White J, Zaninotto P, Walters K, *et al.* Duration of depressive symptoms and mortality risk: the English Longitudinal Study of Ageing (ELSA). *Br J Psychiatry J Ment Sci* 2016;**208**:337–42. doi:10.1192/bjp.bp.114.155333

9 Gold SM, Köhler-Forsberg O, Moss-Morris R, *et al.* Comorbid depression in medical diseases. *Nat Rev Dis Primer* 2020;**6**:1–22. doi:10.1038/s41572-020-0200-2

10 Knowles SE, Chew-Graham C, Adeyemi I, *et al.* Managing depression in people with multimorbidity: a qualitative evaluation of an integrated collaborative care model. *BMC Fam Pract* 2015;**16**:32. doi:10.1186/s12875-015-0246-5

11 Coventry PA, Hays R, Dickens C, *et al.* Talking about depression: a qualitative study of barriers to managing depression in people with long term conditions in primary care. *BMC Fam Pract* 2011;**12**:10. doi:10.1186/1471-2296-12-10

12 Simmonds RL, Tylee A, Walters P, *et al.* Patients' perceptions of depression and coronary heart disease: a qualitative UPBEAT-UK study. *BMC Fam Pract* 2013;**14**:38. doi:10.1186/1471-2296-14-38

13 Raison CL, Miller AH. Is Depression an Inflammatory Disorder? *Curr Psychiatry Rep* 2011;**13**:467–75. doi:10.1007/s11920-011-0232-0

14 Poole L, Steptoe A. The combined association of depressive symptoms and C-reactive protein for incident disease risk up to 12 years later. Findings from the English Longitudinal Study of Ageing (ELSA). *Brain Behav Immun* 2020;**88**:908–12.

For peer review only

For peer review only - http://bmjopen.bmj.com/site/about/guidelines.xhtml

15 DeJean D, Giacomini M, Vanstone M, *et al.* Patient Experiences of Depression and Anxiety with Chronic Disease. *Ont Health Technol Assess Ser* 2013;**13**:1–33.

16 Gask L, Macdonald W, Bower P. What is the relationship between diabetes and depression? A qualitative meta-synthesis of patient experience of co-morbidity. *Chronic Illn* 2011;**7**:239–52. doi:10.1177/1742395311403636

17 Hughes R, Huby M. The construction and interpretation of vignettes in social research. *Soc Work Soc Sci Rev* 2012;**11**:36–51.

18 [dataset] Poole, L., Rowlands, H. The Mood and Health Study, 2018-2019. [data collection]. *UK Data Service* 2021. doi:10.5255/UKDA-SN-854780. https://beta.ukdataservice.ac.uk/datacatalogue/studies/study?id=854780

19 Braun V, Clarke V. Using thematic analysis in psychology. *Qual Res Psychol* 2006;**3**:77–101. doi:10.1191/1478088706qp063oa

20 Braun V, Clarke V. Can I use TA? Should I use TA? Should I not use TA? Comparing reflexive thematic analysis and other pattern-based qualitative analytic approaches. *Couns Psychother Res* 2021;**21**:37–47.

21 Braun V, Clarke V. To saturate or not to saturate? Questioning data saturation as a useful concept for thematic analysis and sample-size rationales. *Qual Res Sport Exerc Health* 2021;**13**:201–16. doi:10.1080/2159676X.2019.1704846

22 Ritchie J, Spencer L. Qualitative data analysis for applied policy research. *Qual Res Companion* 2002;:305–29.

23 Marcu A, Black G, Vedsted P, *et al.* Educational differences in responses to breast cancer symptoms: A qualitative comparative study. *Br J Health Psychol* 2017;**22**:26–41. doi:https://doi.org/10.1111/bjhp.12215

24 Mazure CM. Life stressors as risk factors in depression. *Clin Psychol Sci Pract* 1998;**5**:291–313.

25 Tennant C. Life Events, Stress and Depression: A Review of Recent Findings. *Aust N Z J Psychiatry* 2002;**36**:173–82. doi:10.1046/j.1440-1614.2002.01007.x

26 Wikström I, Jacobsson LT, Arvidsson B. How people with rheumatoid arthritis perceive leisure activities: a qualitative study. *Musculoskeletal Care* 2005;**3**:74–84. doi:10.1002/msc.28

27 Kreider KE. Diabetes Distress or Major Depressive Disorder? A Practical Approach to Diagnosing and Treating Psychological Comorbidities of Diabetes. *Diabetes Ther* 2017;**8**:1–7. doi:10.1007/s13300-017-0231-1

28 Tanenbaum ML, Kane NS, Kenowitz J, *et al.* Diabetes distress from the patient's perspective: Qualitative themes and treatment regimen differences among adults with type 2 diabetes. *J Diabetes Complications* 2016;**30**:1060–8. doi:10.1016/j.jdiacomp.2016.04.023

29 Carney RM, Freedland KE, Eisen SA, *et al.* Major depression and medication adherence in elderly patients with coronary artery disease. *Health Psychol* 1995;**14**:88–90. doi:10.1037/0278-6133.14.1.88

30 Gonzalez JS, Safren SA, Cagliero E, *et al.* Depression, Self-Care, and Medication Adherence in Type 2 Diabetes: Relationships across the full range of symptom severity. *Diabetes Care* 2007;**30**:2222–7. doi:10.2337/dc07-0158

For peer review only - http://bmjopen.bmj.com/site/about/guidelines.xhtml

31 Jerant A, Kravitz R, Moore-Hill M, *et al.* Depressive Symptoms Moderated the Effect of Chronic Illness Self-Management Training on Self-Efficacy. *Med Care* 2008;**46**:523–31.

32 NHS England. The Improving Access to Psychological Therapies (IAPT) Pathway for People with Long-term Physical Health Conditions and Medically Unexplained Symptoms. 2018.https://www.england.nhs.uk/wp-content/uploads/2018/03/improving-access-to-psychological-therapies-long-term-conditions-pathway.pdf

33 Swain S, Sarmanova A, Mallen C, *et al.* Trends in incidence and prevalence of osteoarthritis in the United Kingdom: findings from the Clinical Practice Research Datalink (CPRD). *Osteoarthritis Cartilage* 2020;**28**:792–801. doi:10.1016/j.joca.2020.03.004

For peer review only

Table 1: Characteristics of the sample, stratified by group

	Whole sample (N= 41) *M±SD or N(%)*	Depression only (n = 12) *M±SD or N(%)*	Depression and CHD (n = 5) *M±SD or N(%)*	Depression and T2D (n = 10 *M±SD or N(%)*	Depression and arthritis (n = 14) *M±SD or N(%)*
Sex (female)	24(58.5)	6(50.0)	1(20.0)	4(40.0)	13(92.9)
Age (years)	63.5(5.5)	62.4(5.5)	66.0(4.0)	61.0(5.2)	65.1(5.9)
Married/cohabiting	17(41.5)	6(50.0)	2(40.0)	5(50.0)	4(28.6)
Ethnicity:					
White British	27(65.9)	10(83.3)	2(20.0)	6(60.0)	9(64.3)
Indian	3(7.3)			2(20.0)	1(7.1)
Pakistani	1(2.4)		1(20.0)		
Black Caribbean	1(2.4)		1(20.0)		
Other White background	7(17.1)	2(16.7)	1(20.0)	1(10.0)	3(21.4)
Other Asian background	2(4.9)			1(10.0)	1(7.0)
Employment status:					
Employed full-time	9(22.0)	3(25.0)	1(20.0)	3(30.0)	2(14.3)
Employed part-time	1(2.4)	1(8.3)			
Self-employed	7(17.1)	3(25.0)			4(28.6)
Retired	15(36.6)	4(33.3)	3(60.0)	3(30.0)	5(35.7)
Unemployed	4(9.8)	1(8.3)		3(30.0)	
Volunteer	2(4.9)		1(20.0)		1(7.1)
Disabled	3(7.3)			1(10.0)	2(14.3)
Highest educational qualification - degree or higher	15(36.6)	3(25.0)	3(60.0)	4(40.0)	5(35.7)
Antidepressant usage (yes)	35(85.4)	9(75.0)	4(80.0)	9(90.0)	13(92.9)

For peer review only - http://bmjopen.bmj.com/site/about/guidelines.xhtml

For peer review only

Figure 1 Caption *(figure itself attached as separate .jpg file)*

Figure 1. Schematic diagram of theme structure.

N.B. LTC: Long term condition.

For peer review only

For peer review only - http://bmjopen.bmj.com/site/about/guidelines.xhtml

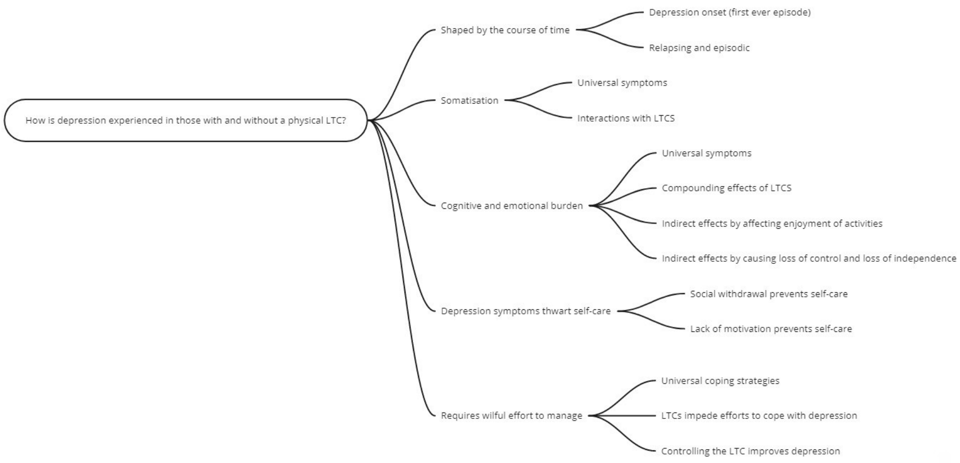

Figure 1. Schematic diagram of theme structure.
N.B. LTC: Long term condition.

372x187mm (96 x 96 DPI)

Supplementary file: Appendix 1 - Vignettes

Scenario 1

Sarah has good days and bad, but on a bad day she feels so sad and miserable that she just can't stand it. It seems to completely take over her head like she's in a fog. She feels really disappointed in herself for feeling this way, despite all the good things she has in her life. Sometimes when she's by herself she cries and even has thoughts of killing herself just to try and be anywhere but here. She feels completely worthless.

Scenario 2

Most days Sam wakes up and feels so tired he wishes he could just stay in bed. In fact he feels so tired most of the time that he doesn't want to do anything. He sleeps longer than he used to and doesn't have the energy to do anything. Sometimes he doesn't even feel like eating. He's even lost weight without trying to. His partner tries to show him affection and wants a kiss and a cuddle, but he just feels numb and not interested at all.

Scenario 3

Jason finds it really hard to feel positive about the future at the moment, it's like he has nothing to look forward to anymore. He finds it difficult to concentrate and to make decisions these days too. He feels so frustrated and trapped by the way he feels. His friends tell him he should snap out of it, but he just can't. He wishes he didn't have to feel like this, it makes him feel like such a failure.

Scenario 4

Most days Clare feels like she is being punished for something she must have done in a past life. And then she feels guilty for thinking like this. She seems to worry about her health all the time and her aches and pains get on top of her. She doesn't enjoy things the way she used to, in fact she doesn't really get any pleasure out of anything anymore. She finds it hard to leave the house some days. Everything seems a bit pointless these days, if she's being completely honest.

Scenario 5

Susan has always enjoyed her food, but for the past couple of months she's noticed she's been putting on some extra weight. She finds she craves her favourite foods and is comfort eating to make herself feel a bit better. She's not sure she is feeling any better though, in fact she's sleeping really badly and often wakes up several times in the night. Her sleep is restless and she wakes up tired making each day feel like it drags.

For peer review only - http://bmjopen.bmj.com/site/about/guidelines.xhtml

LONDON'S GLOBAL UNIVERSITY

DEPARTMENT OF BEHAVIOURAL SCIENCE AND HEALTH

The Mood and Health Study

Interview Schedule – Depression

Introduction

Review of Participant Information Sheet

Consent form

Timing and confidentiality

My name is [insert name] and I am a researcher. I'm not a doctor or a psychologist and I don't have any clinical contact with your team. I'm interested in finding out from you what your experiences have been with your mood, and there are no right answers.

Vignettes

To start I'd like you to look at what some other people have experienced, and then we'll talk about them a bit. Firstly, I'd like you to start by sorting the cards into two piles, those which you think are relevant to you and those which aren't.

**Card sort**	Can you talk me through how you have sorted the cards?<br>Has anything like this ever happened to you?<br>Have you ever felt these sorts of things before?<br>How do you think this person's experience is different from yours?

Depression

**Precipitating events**	Take me back, can you tell me about when you first noticed changes to your mood?

For peer review only - http://bmjopen.bmj.com/site/about/guidelines.xhtml

Prompts	• Can you remember feeling differently?<br>• How did it feel?<br>• Was there anything else you think may have contributed to your low mood?<br>• How do you think that experience relates to how you feel today?
**Diagnosis**	Can you talk me through the support you received during this time?
Prompts	• E.g. family, friends, GP?<br>• How did you find out you had depression? E.g. Who told you? When?<br>• How did you feel when you found out it was depression?
**Timeline**	How has your mood been since then?
Prompts	• How has you mood changed over time?
**Symptoms**	Can you tell me about a time when you felt your depression was at its worst?
Prompts	• How did it feel?<br>• How long did it last?<br>• Can you tell me how most days are for you at the moment?<br>• What's a good day for you?<br>• How long have you been feeling this way?<br>• Are there times when you don't feel this way? How long does this last?
**Management of depression**	Are you receiving any treatment for your depression at the moment?
Prompts	• Can you tell me about the care you have received for your depression from doctors?<br><br>e.g. who (GP, clinic nurse/doctor)<br>e.g. where (GP clinic, hospital clinic, community/third sector)<br>e.g. when (routine/booked appointments, length of appointments)

For peer review only - http://bmjopen.bmj.com/site/about/guidelines.xhtml

	e.g. what (antidepressants, counselling)<br>• Do you find it easy to talk to your doctor about your concerns?<br>• How did you find the treatment?<br>• How helpful has this treatment been? E.g. what was most helpful and least helpful?
**Self-care**	• Can you talk me through anything you do yourself that helps you cope better?
Prompts	• For example, exercise, eating habits, alcohol consumption, smoking, acceptance, distractions/hobbies, wallowing. Have any of these been helpful to you?<br>• How do these things make you feel?

Wrapping up

Is there anything else you would like to tell me?

How have you found this interview?

Do you have any questions?

Thank you for your time.

Give sources of further support handout.

Give baseline questionnaire.

Provide participant with £10 retail voucher.

For peer review only - http://bmjopen.bmj.com/site/about/guidelines.xhtml

LONDON'S GLOBAL UNIVERSITY

DEPARTMENT OF BEHAVIOURAL SCIENCE AND HEALTH

The Mood and Health Study

Interview Schedule – Depression and Physical Illness

Introduction

Review of Participant Information Sheet

Consent form

Timing and confidentiality

My name is [insert name] and I am a researcher. I'm not a doctor or a psychologist and I don't have any clinical contact with your team. I'm interested in finding out from you what your experiences have been with your mood and health, and there are no right answers.

Vignettes

To start I'd like you to look at what some other people have experienced, and then we'll talk about them a bit. Firstly, I'd like you to start by sorting the cards into two piles, those which you think are relevant to you and those which aren't.

**Card sort**	Can you talk me through how you have sorted the cards?<br>Has anything like this ever happened to you?<br>Have you ever felt these sorts of things before?<br>How do you think this person's experience is different from yours?

Depression

**Precipitating events**	Take me back, can you tell me about when you first noticed changes to your mood?

For peer review only - http://bmjopen.bmj.com/site/about/guidelines.xhtml

Prompts	• Can you remember feeling differently?<br>• How did you feel?<br>• Was there anything else you think may have contributed to your low mood?<br>• How do you think that experience relates to how you feel today?
**Diagnosis**	Can you talk me through the support you received during this time?
Prompts	• E.g. family, friends, GP?<br>• How did you find out you had depression? E.g. Who told you? When?<br>• How did you feel when you found out it was depression?
**Timeline**	How has your mood been since then?
Prompts	• How has you mood changed over time?
**Diagnosis of physical illness (if not covered above)**	Can you tell me how you found out about your [arthritis/heart disease/diabetes]?
Prompts	• How long ago was this?<br>• Who told you? Where were you?<br>• How did you feel when you found out?<br>• Did you find out about your [arthritis/heart disease/diabetes] before or after you found out you had depression? How did you find this time?
**Symptoms**	Can you tell me about a time when you felt your depression was at its worst?
Prompts	• How did it feel?<br>• How long did it last?<br>• Can you tell me how most days are for you at the moment?<br>• What's a good day for you?

For peer review only

	• How long have you been feeling this way?<br>• Are there times when you don't feel this way? How long does this last?
**Interaction**	Can you tell me about how your [arthritis/heart disease/diabetes] affects your life now?
	• How does your [arthritis/heart disease/diabetes] make you feel?<br>• How do your depression and [arthritis/heart disease/diabetes] affect each other?<br>• Have you ever found it difficult to cope with [arthritis/heart disease/diabetes]?<br>e.g. coping with the symptoms, treatments?
**Management**	Are you receiving any treatment for your depression at the moment?
Prompts	• Can you tell me about the care you have received for your depression from doctors?<br>e.g. who (GP, clinic nurse/doctor)<br>e.g. where (GP clinic, hospital clinic, community/third sector)<br>e.g. when (routine/booked appointments, length of appointments)<br>e.g. what (antidepressants, counselling)<br>• How did you find the treatment?<br>• How helpful has this treatment been? E.g. what was most helpful and least helpful?<br>• How easy have you found it to get help for your depression?
	What treatments have you had for your [arthritis/heart disease/diabetes]?
Prompts	e.g. who (GP, clinic nurse/doctor)<br>e.g. where (GP clinic, hospital clinic)<br>e.g. when (more than one treatment?)<br>e.g. what (self-management courses, medication, surgery?)

For peer review only - http://bmjopen.bmj.com/site/about/guidelines.xhtml

	• Do you find it easy to talk to your doctor about your concerns?<br>• How did you find the treatment?<br>• How helpful has this treatment been? E.g. what was most helpful and least helpful?<br>• How do you feel treatment of your [arthritis/heart disease/diabetes] has affected your depression?<br>• **[Arthritis and angina only]** How have the treatments helped with your pain?
**Self-care**	• Can you talk me through anything you do yourself that helps you cope better?
Prompts	• For example, exercise, eating habits, alcohol consumption, smoking, acceptance, distractions/hobbies, wallowing. Have any of these been helpful to you?<br>• How do these things make you feel?

Wrapping up

Is there anything else you would like to tell me?

How have you found this interview?

Do you have any questions?

Thank you for your time.

Give sources of further support handout.

Give baseline questionnaire.

Provide participant with £10 retail voucher.

For peer review only

For peer review only - http://bmjopen.bmj.com/site/about/guidelines.xhtml

COREQ (COnsolidated criteria for REporting Qualitative research) Checklist

A checklist of items that should be included in reports of qualitative research. You must report the page number in your manuscript where you consider each of the items listed in this checklist. If you have not included this information, either revise your manuscript accordingly before submitting or note N/A.

Topic	Item No.	Guide Questions/Description	Reported on Page No.
**Domain 1: Research team and reflexivity**			
Personal characteristics			
Interviewer/facilitator	1	Which author/s conducted the interview or focus group?	7
Credentials	2	What were the researcher's credentials? E.g. PhD, MD	7
Occupation	3	What was their occupation at the time of the study?	7
Gender	4	Was the researcher male or female?	7
Experience and training	5	What experience or training did the researcher have?	7
Relationship with participants			
Relationship established	6	Was a relationship established prior to study commencement?	7
Participant knowledge of the interviewer	7	What did the participants know about the researcher? e.g. personal goals, reasons for doing the research	7
Interviewer characteristics	8	What characteristics were reported about the inter viewer/facilitator? e.g. Bias, assumptions, reasons and interests in the research topic	7
**Domain 2: Study design**			
Theoretical framework			
Methodological orientation and Theory	9	What methodological orientation was stated to underpin the study? e.g. grounded theory, discourse analysis, ethnography, phenomenology, content analysis	8
Participant selection			
Sampling	10	How were participants selected? e.g. purposive, convenience, consecutive, snowball	6
Method of approach	11	How were participants approached? e.g. face-to-face, telephone, mail, email	6
Sample size	12	How many participants were in the study?	9
Non-participation	13	How many people refused to participate or dropped out? Reasons?	9
Setting			
Setting of data collection	14	Where was the data collected? e.g. home, clinic, workplace	6
Presence of non-participants	15	Was anyone else present besides the participants and researchers?	6
Description of sample	16	What are the important characteristics of the sample? e.g. demographic data, date	9
Data collection			
Interview guide	17	Were questions, prompts, guides provided by the authors? Was it pilot tested?	7
Repeat interviews	18	Were repeat inter views carried out? If yes, how many?	N/A
Audio/visual recording	19	Did the research use audio or visual recording to collect the data?	7
Field notes	20	Were field notes made during and/or after the inter view or focus group?	7
Duration	21	What was the duration of the inter views or focus group?	6
Data saturation	22	Was data saturation discussed?	6&8
Transcripts returned	23	Were transcripts returned to participants for comment and/or	24

For peer review only

For peer review only - http://bmjopen.bmj.com/site/about/guidelines.xhtml

Topic	Item No.	Guide Questions/Description	Reported on Page No.
		correction?	
**Domain 3: analysis and findings**			
Data analysis			
Number of data coders	24	How many data coders coded the data?	8
Description of the coding tree	25	Did authors provide a description of the coding tree?	8
Derivation of themes	26	Were themes identified in advance or derived from the data?	8
Software	27	What software, if applicable, was used to manage the data?	8
Participant checking	28	Did participants provide feedback on the findings?	24
Reporting			
Quotations presented	29	Were participant quotations presented to illustrate the themes/findings? Was each quotation identified? e.g. participant number	10-20
Data and findings consistent	30	Was there consistency between the data presented and the findings?	10-20
Clarity of major themes	31	Were major themes clearly presented in the findings?	10-20
Clarity of minor themes	32	Is there a description of diverse cases or discussion of minor themes?	10-20

Developed from: Tong A, Sainsbury P, Craig J. Consolidated criteria for reporting qualitative research (COREQ): a 32-item checklist for interviews and focus groups. *International Journal for Quality in Health Care*. 2007. Volume 19, Number 6: pp. 349 – 357

Once you have completed this checklist, please save a copy and upload it as part of your submission. DO NOT include this checklist as part of the main manuscript document. It must be uploaded as a separate file.

For peer review only

The experience of depression in older adults with and without a physical long-term condition: findings from a qualitative interview study.

Journal:	*BMJ Open*
Manuscript ID	bmjopen-2021-056566.R2
Article Type:	Original research
Date Submitted by the Author:	02-Feb-2022
Complete List of Authors:	Poole, Lydia; UCL, Institute of Health Informatics<br>Frost, Rachael; University College London Research Department of Primary Care and Population Health, Research Department of Primary Care and Population Health<br>Rowlands, Hannah; King's College London<br>Black, Georgia; University College London, Applied Health Research; Dr
**Primary Subject Heading**:	Mental health
Secondary Subject Heading:	Qualitative research, Health policy
Keywords:	Depression & mood disorders < PSYCHIATRY, QUALITATIVE RESEARCH, Coronary heart disease < CARDIOLOGY, DIABETES & ENDOCRINOLOGY, RHEUMATOLOGY

SCHOLARONE™ Manuscripts

I, the Submitting Author has the right to grant and does grant on behalf of all authors of the Work (as defined in the below author licence), an exclusive licence and/or a non-exclusive licence for contributions from authors who are: i) UK Crown employees; ii) where BMJ has agreed a CC-BY licence shall apply, and/or iii) in accordance with the terms applicable for US Federal Government officers or employees acting as part of their official duties; on a worldwide, perpetual, irrevocable, royalty-free basis to BMJ Publishing Group Ltd ("BMJ") its licensees and where the relevant Journal is co-owned by BMJ to the co-owners of the Journal, to publish the Work in this journal and any other BMJ products and to exploit all rights, as set out in our licence.

The Submitting Author accepts and understands that any supply made under these terms is made by BMJ to the Submitting Author unless you are acting as an employee on behalf of your employer or a postgraduate student of an affiliated institution which is paying any applicable article publishing charge ("APC") for Open Access articles. Where the Submitting Author wishes to make the Work available on an Open Access basis (and intends to pay the relevant APC), the terms of reuse of such Open Access shall be governed by a Creative Commons licence – details of these licences and which Creative Commons licence will apply to this Work are set out in our licence referred to above.

Other than as permitted in any relevant BMJ Author's Self Archiving Policies, I confirm this Work has not been accepted for publication elsewhere, is not being considered for publication elsewhere and does not duplicate material already published. I confirm all authors consent to publication of this Work and authorise the granting of this licence.

For peer review only

The experience of depression in older adults with and without a physical long-term condition: findings from a qualitative interview study.

Lydia Poole[1], Rachael Frost[2], Hannah Rowlands[3], Georgia Black[4].

[1] Institute of Health Informatics, University College London, 222 Euston Road, London, NW1 2DA.

[2] Research Department of Primary Care and Population Health, Upper Third Floor, UCL Medical School (Royal Free Campus), Rowland Hill Street, London, NW3 2PF.

[3] Department of Psychological Medicine, Institute of Psychiatry, Psychology & Neuroscience, Kings College London, E3.18, De Crespigny Park, London, SE5 8AF.

[4] Department of Applied Health Research, University College London, 1-19 Torrington Place, London, WC1E 6BT.

Word count: 8119

Correspondence to:
Dr Lydia Poole
Institute of Health Informatics
222 Euston Road
University College London
London
NW1 2DA

T: +44 (0)20 3108 8817
E: lydia.poole@ucl.ac.uk

For peer review only - http://bmjopen.bmj.com/site/about/guidelines.xhtml

Abstract

Objective: To understand how the lived experience of depression differs among patients with a long-term condition (LTC) compared to those without an LTC, and how the experience differs across different types of LTC.

Design: Face-to-face, semi-structured, interviews.

Setting: Primary care; GP surgeries in and around North London.

Participants: 41 primary care patients with depression were recruited. Our sample comprised participants aged 55-75 years old with depression only (n= 12), depression and coronary heart disease (n=5), depression and type 2 diabetes (n=10) and depression and arthritis (n=14).

Results: Interviews were conducted, audio-recorded, transcribed, and analysed using thematic analysis. The results revealed that the cardinal diagnostic symptoms of depression (anhedonia, sadness) were experienced by all our participants regardless of LTC. However, the LTC did interact with depression by compounding somatic, cognitive and emotional symptoms, increasing disability and reducing independence, and hindering attempts at coping with mental illness. Our findings demonstrate common experiences across patients, as well as key differences based on LTC.

Conclusions: We suggest four key implications for future care practices of these patients: (1) Not all participants with depression and LTC view their mental and physical health as interconnected; there should be allowances in care plans for separate treatment pathways; (2) Key features of depression that affect LTC management are social withdrawal and lack of motivation to self-manage or access healthcare; (3) Key features of LTCs that worsen depression are pain, the unpredictability of future health and progressive disability; (4) Positive self-management of LTC could improve self-efficacy and therefore mood, and should be encouraged.

For peer review only - http://bmjopen.bmj.com/site/about/guidelines.xhtml

Key words: Depression; long-term conditions; co-morbidity; qualitative research; interview study; thematic analysis.

Strengths and limitations of the study

- Participant diagnoses derived from clinical records and confirmed by self-report.
- Participants were drawn from across multiple sites across North London, with diversity in demography achieved.
- Comparative thematic analysis allowing for comparisons within and between groups.
- Small sample of depression and coronary heart disease cohort.
- Majority female in the depression and arthritis cohort.

For peer review only - http://bmjopen.bmj.com/site/about/guidelines.xhtml

Introduction

Estimates from the UK Biobank study suggest the prevalence for a single episode of major depressive disorder is 6.4%, varying to 12.2% for moderate recurrent major depression and 7.2% for severe recurrent major depression,[1] among British adults. Depression has been associated with increased all-cause mortality;[2] however causality has not yet been determined.[3] One possible explanation is that individuals with depression are more than twice as likely to have a concomitant physical long-term condition (LTC), such as diabetes and coronary heart disease.

It has been hypothesised from quantitative investigations of epidemiological and clinical cohort studies that depressive symptoms which occur in cardiac patients are distinct from those experienced in psychiatric populations based on three key differences: composition of symptoms, timeline, and treatment responsiveness.[4] With regard to symptoms, the hallmarks of psychiatric depression are persistent sadness and loss of interest and pleasure in activities (anhedonia). These are often associated with cognitive changes such as a sense of worthlessness and guilt, among others. By contrast, there is growing recognition that depressive symptoms in people with physical illness are dominated by somatic symptoms, such as fatigue and loss of energy.[5,6] In addition, it has been suggested that the chronicity, and the timing of onset of depressive symptoms in relation to the LTC, have implications for morbidity and mortality.[7,8] Finally, it appears that different treatment strategies may be appropriate for those living with psychiatric depression compared to those who have comorbid depression,[9] with particular benefit coming from integrative collaborative care models of healthcare.[10] Qualitative data has revealed that depression in physical illness has been shown to have been normalised by health care practitioners leading to barriers in its management [11] and there may be resistance to antidepressant medication among some LTC patients.[12] Given these empirical observations surrounding the differences in depression symptoms, timeline and treatment, some authors have suggested that a separate subtype of depression may exist, driven by biological (inflammatory) changes [13] which in turn are implicated in LTCs.[14] However, despite a plausible hypothesis for the existence of a separate subtype of depression in those with an LTC, whether or not this translates to differences in patient experience

For peer review only - http://bmjopen.bmj.com/site/about/guidelines.xhtml

is unclear. Therefore, further qualitative research is warranted to examine whether the lived experience of depression is similar or different in patients with depression and/or a comorbid LTC.

Furthermore, experiences of depression across LTCs are likely to differ. A meta-synthesis of qualitative data has suggested that patients with depression and a comorbid LTC either see their mental and physical health as either separate and independent from each other, or interrelated with one having caused or worsened the other.[15] However, the similarities and/or differences in how depression is experienced in different LTCs is currently not well-understood. For example, depression in diabetes has been revealed to be heterogenous with aspects of diabetes-related distress and demoralisation being apparent,[16] while depression in CHD has been associated with loss, both in terms of interpersonal loss as well as loss of health/control.[12]

Therefore, the aim of this study was twofold. First, we aimed to examine how the lived experience of depression is similar or different between those with an LTC and those without, and second, we aimed to understand how experiences of depression are similar or different between types of LTC. In order to take into account the correlation between age and LTC onset, we focussed on the experiences of depression in adults in mid-late life.

Methods

Participant selection and recruitment

Thirty-seven participants were recruited using mailouts from 14 UK general practices in the Barnet and Enfield regions of North London; a further one participant was recruited using opportunistic sampling from GP surgeries in North West London; and a final three participants were recruited from the Camden and Islington IAPT (Improving Access to Psychological Therapy) service.

Our inclusion criteria led us to recruit participants who identified as male and female, aged 55-75 years old and English speaking. All participants were screened for current depression by participating GP practices via electronic and hand searches of medical records. Current depression was defined as a patient who had either received a clinical code of depression by their GP and/or had received a referral or prescription for

For peer review only

depression treatment in the preceding two months. All participants were medically stable outpatients living within the community. We excluded patients with a severe cognitive, mental or psychological impairment that would impede their ability to provide informed consent. Moreover, to compare patients living with depression, with and without an LTC, we recruited patients to one of four participant groups according to additional diagnoses identified in their medical notes: depression only, depression and CHD, depression and T2D, and depression and arthritis. To reduce cross-over between the groups, participants with more than one LTC of interest (i.e. diabetes and arthritis) in their medical notes were excluded. We also excluded those with another LTC such as cancer, stroke and chronic lung disease to allow us to focus our discussions more easily on our chosen LTCs. The LTCs were selected based on *a priori* knowledge to elicit a variety of illness experiences: coronary heart disease can have an acute, life-threatening presentation (e.g. acute coronary syndromes); T2D involves monitoring and self-management of blood sugar levels and can lead to acute episodes of illness (hypoglycaemia); arthritis is associated with pain and functional impairment.

Practice GPs reviewed the list of those to approach prior to mailout and removed people that met the exclusion criteria or they considered inappropriate to contact. The mail-out included full details of the study and interested patients were invited to contact the research team to discuss participation. Patients were screened by researchers over the telephone using the same inclusion and exclusion criteria before arranging a convenient time and place for the interview to take place. We used purposive sampling to capture a range of clinical and demographic characteristics within each group where possible; specifically, among those patients who expressed an interest in taking part we prioritised telephone screening to allow us to recruit a diverse sample by sex, age, ethnicity and location.

Interview data collection

We conducted 1:1 semi-structured face-to-face interviews, mainly at participants' homes or in a meeting room at UCL (or another convenient location if requested), lasting approximately one hour (range 42–97 minutes). Interviews were carried out between May to October 2019. Data collection continued within this time frame and ceased once all eligible patients at participating sites had been contacted. Interviews were conducted by

For peer review only - http://bmjopen.bmj.com/site/about/guidelines.xhtml

two female researchers, HR (MSc; research assistant) (n = 40) or LP (PhD; senior research fellow) (n = 1). The interviewers had received training in qualitative interviewing, and HR had prior experience of conducting qualitative interviews with vulnerable groups. Neither HR or LP had any prior connection with participants, other than the contact made during the recruitment process where the study objectives and procedures were explained. The interviews began by presenting participants with four short vignettes for participants to read and reflect on. The vignettes contained the experiences of fictional characters written in the third person (see Appendix 1) and were designed to encourage participants to reflect on their own symptoms of depression. This method is well-established for eliciting detailed information on sensitive topics in qualitative research.[17] The interview then covered a number of topics, to explore the experience of living with depression: diagnosis, precipitating events, symptoms, change in experience over time, management of depression and treatment preferences. In participants with a comorbid LTC we also explored the impact of depression on their physical illness and vice versa. The topic guides (see Appendix 2) were modified as interviews progressed, through team discussions. To circumvent issues surrounding stigma, initial questions steered clear of clinical labels, and instead invited participants to reflect on 'changes to their mood'. As each interview progressed the interviewer used the participants' own terminology. After each interview, demographic data and self-reported medical information were collected. The primary interviewer (HR) kept a reflective diary in order to document thoughts, feelings and other interesting contextual information that occurred to her. Participants were provided with relevant local and national mental health service information and a £10 retail voucher honorarium. Any concerns over participant safety (including suicidal ideation and self-harm) were discussed with the senior researcher (LP) before contacting participants' GP with their consent. A Standard Operating Procedure was in place for this. Interviews were audio-recorded, with brief field notes, transcribed verbatim by an external company and anonymised and verified for accuracy by the research team (HR and LP). Data for this study are archived in a public data repository.[18]

Patient and Public Involvement

For peer review only - http://bmjopen.bmj.com/site/about/guidelines.xhtml

Patients and public were not involved in the development of the research question, design or study implementation. However, the results of this study were disseminated via a large outreach and engagement event through social media and podcasting platforms.

Data analysis

We used reflexive thematic analysis [19,20] to analyse our data from a realist/essentialist perspective in which a (mostly) one-way relationship is taken to operate between meaning, experience and language. According to this approach, data saturation is not required, rather meaning is conceived as developing from the interpretation of the data.[21] All transcripts contained the entire interview, including the discussion that took place regarding the material in the vignettes. The data arising from the discussion of vignettes varied across participants; some participants did not engage with the vignettes and the conversation quickly flowed to the semi-structured interview questions, whereas for others it prompted a divulgement of prior personal events. As such, these data were not separated, but rather the entire transcript was treated as a single data piece. Transcripts were read by HR (a health psychology researcher expertise in LTCs) and LP (a health psychology researcher with an interest in depression and multimorbidity); GB (a health services researcher with expertise in health improvement) and RF (a health services researcher with expertise in ageing and mental health) read a subsample. Data analysis was led by LP. Transcripts from each group were first read and analysed inductively, separately, to enable us to build an understanding of the idiographic qualities of the dataset;[19] inductive analysis is a bottom-up approach where the codes and themes are driven by the data and not by a theoretical framework. Each set of transcripts was read repeatedly, and codes were developed by LP in conjunction with HR. HR's reflective diary was consulted to ensure no additional meaning or interpretation could be gleaned from the data. Discrepancies were discussed until consensus was reached. Coding of transcripts was performed using NVivo 12 (QSR International Pty Ltd., 2018). A codebook was kept to organise ideas and for note keeping. For example, our coding tree included codes for the onset and timeline of depressive symptoms, stressful life events, cognitive symptoms of depression, somatic symptoms of depression, past decisions, social connections, emotional response to illness, physical illness impacting daily living, the symptoms of the physical

For peer review only - http://bmjopen.bmj.com/site/about/guidelines.xhtml

illness, approaching death, coping strategies and help-seeking and attitudes towards care. Codes were then grouped to construct themes; refined through team discussions and piloting. After conducting the initial analysis across the entire dataset, we tabulated the themes and subthemes using Excel (Microsoft for Windows 365) to allow us to systematically explore the similarities and differences in experiences across groups. This approach was informed by Framework Analysis [22] and has been combined with thematic analysis by previous researchers conducting comparative qualitative analysis.[23] The indexing and charting of the data enabled us to maintain rigour in the analytic process. Themes were then written out and refined with feedback from team. The study was approved by NHS London – Fulham Research Ethics Committee (reference: 18/LO/2150).

Results

Participants

Out of 137 patients who expressed an interest in the study, 45 were eligible and 41 completed interviews. Reasons for ineligibility included not meeting the clinical inclusion criteria (n = 37), non-responsive to return phone calls (n = 26), not interested (n = 3), unavailable for interview (n =1), sample size deemed sufficient for the cohort (n = 23), non-English speaker (n = 2). Participants were grouped into 4 cohorts based on their medical records and verified during screening telephone calls: depression only (n = 12), depression and CHD (n = 5) and depression and type 2 diabetes (T2D) (n = 10) and depression and arthritis (n = 14). In terms of CHD, patients in this group had received a diagnosis for myocardial infarction (n = 1), angina (n=2) and heart failure (n = 2). In terms of arthritis, patients in this group included those with rheumatoid arthritis (n = 2) and osteoarthritis (n = 12). Overall, participants were aged 63.5 years (standard deviation (SD) 5.5) and we sampled roughly equal numbers of men and women. However, the majority of depression and arthritis participants were female (93%). Approximately one-third of the sample were from a non-White ethnic group and 85.4% of all participants were taking antidepressant medication(s) at the time of the interview. The characteristics of the sample are presented in Table 1.

Thematic overview

For peer review only - http://bmjopen.bmj.com/site/about/guidelines.xhtml

For peer review only

We generated five overarching themes that encompass the experience of living with depression in those with and without an LTC. In each theme we have drawn out the relationship between the experience of depression in those with and without an LTC, and where relevant, compared this across the different LTC groups (CHD, T2D and arthritis). Across all the themes, we found that the experience of depression and LTCs acted to compound the effects of each other; this was particularly apparent for those whose illness had resulted in changes to daily living. Figure 1 presents a schematic diagram of our themes.

Theme 1: Shaped by the course of time

Across our sample, depression was not static; it was shaped by individuals' life histories and past events. The experience of depression also shifted over time so that as people's lives progressed, different triggers for new episodes became more apparent. In those with an LTC, hardship brought about by their LTC and a loss of health was described by some as a trigger for depression.

Depression onset (first-ever episode)

In participants with and without LTCs, the onset of the first-ever episode of depression occurred at different ages; many could identify roots for their depression in childhood or adolescence, while others had received a diagnosis more recently. Across the groups, most participants were able to identify a precipitating adverse life event(s) to their first-ever depressive episode such as financial insecurity, (multiple) bereavement, a relationship breakdown, children taken into care, and physical and sexual abuse. For some, seemingly smaller grievances, had accumulated over time to initiate the first-ever episode of depression.

In contrast to the depression-only participants, in participants with an LTC, life histories revealed two separate discourses relating to the onset of the initial depression diagnosis: coincidental and interconnected. As such, for some, the LTC was coincidental to the onset of the depression, with the depression and LTC diagnoses occurring at different times and in relation to different precipitating/surrounding events. This discourse was predominately discussed by those with T2D, and to a lesser extent the CHD cohort. These individuals had a clear narrative about the onset of their depression, that was related to other life pressures such as caring responsibilities, fraud allegations and work rather than to their LTC:

For peer review only - http://bmjopen.bmj.com/site/about/guidelines.xhtml

"I don't think so. I don't really think so. I had no qualms about my health. It was the other things. The inferiority complex, the pressure of work and the unhappiness with my married life. It was those things." **(P.39, depression and T2D, male)**.

"I honestly can't say that [depression] is the reason of that [CHD]. But yes, when you have I suppose chest pain you think about it, you sit down, you [pause]. But I don't think it took me into depression at that time or anxiety or something." **(P.45, depression and CHD, male)**.

The second discourse highlighted the interconnected nature of the initial depression and LTC diagnosis. For these participants, depression was a new experience, and was directly attributed to their LTC, or its implications (e.g. hospitalisations, loss of mobility, job loss). The type of LTC did not make a difference, only whether the LTC was associated with unpredictable, major, and irreversible upheaval.

"Depressed? It was when I came out of hospital after having my triple bypass because my life changed completely. I lost my job, my relationship broke up, the kids and all that and I just sort of had a sort of breakdown and that was it really." **(P.44, depression and CHD, male)**.

Relapsing and episodic

Across all participants, after the initial onset, the experience of depression had a varying time course. A few participants had new onset depression. However, for the majority of participants, their history of depression was chronic, with repeated episodes over time. This had led to a familiarity with when a new 'bout' was coming on, even if there was no discernible trigger.

Where there was a clear trigger for a new episode of depression, this was often a difficult life event, such as a child going to prison or a transition to retirement. However, for some people with chronic, pre-existing depression, the LTC had also triggered a new episode. This led to a new experience of depression for these participants, which was not centred around their earlier difficult life circumstances which had originally brought about their depression. Instead, the LTC raised fears surrounding declining health, death and frailty, which fed into the new depressive episode:

For peer review only - http://bmjopen.bmj.com/site/about/guidelines.xhtml

"The depression since than that was different. It was felt trapped then … I was full of tension, full of uncomfortable feeling with him. I just couldn't, it was like having a snake you know circling around you know... So that is thank God I don't have that. The depression that I feel in this and that is because of my health problems and that bothers me, that bothers me a lot." **(P.22, depression and CHD, female)**.

Theme 2: Somatisation

Somatic symptoms of depression were frequently experienced by our participants, in those with and without an LTC. The experience was largely similar across our four cohorts. Differences did exist in sleep disturbance and crying, with LTCs (mainly arthritis but also T2D) contributing to the experience of these somatic symptoms.

Universal somatic symptoms

Somatic symptoms were discussed by all our participants, irrespective of LTC. Symptoms discussed included appetite changes (including over- and under-eating), crying, and loss of intimacy. By far the most common of all the somatic complaints was sleep disturbance. Some described difficulties falling asleep, restless and disturbed sleep, vivid dreams and nightmares, and difficulty getting out of bed in the morning. Psychomotor retardation was another common somatic complaint linked to fatigue, which was experienced across all groups, irrespective of LTC.

"Really, really very slow, very sluggish. Very physically, very physical. In fact, almost as physical as it is mental…. Yeah like you're swimming. Like you're swimming against a sea of something coming at you. And you're trying to wade yourself through and knowing that if you, and no energy, zero physical energy." **(P.13, depression only, female)**

Somatic symptoms and interactions with LTCs

For those with an LTC, the illness also directly contributed to the experience of somatic symptoms of depression. Sleep was negatively affected by the LTC through worries about managing the condition (as with P.2 with T2D) or pain. Pain was particularly associated with participants in the depression and arthritis cohort.

For peer review only - http://bmjopen.bmj.com/site/about/guidelines.xhtml

"I haven't been a very good sleeper lately; I don't sleep the full 8 hours that they say you should do. I do think about things and one of the things obviously I do think about is make sure my diabetes is correct." **(P.2, depression and T2D, male)**.

"I can't sleep at night because of pain… And even at night I get a lot of pain in my back and that so I am just putting my legs round 10 times this way and 10 times that way because I can't sleep." **(P.4, depression and arthritis, female)**.

While crying was frequently described across all our participants, in our arthritis participants we observed a unique contribution of the LTC. Arthritis (and in particular arthritic pain) compounded the emotional and somatic experience of depression, and could prompt tears of fear and frustration.

"I am chopping up the onions, I have to go and sit down because the pain in my back is such that I can't, I have to sit down. And like I have cried with it, not just from the onions but real tears, through frustration I suppose and pain." **(P.41, depression and arthritis, female)**.

Theme 3: Cognitive and emotional burden

In this theme the participants' lived experience of the cognitive and emotional symptoms of depression are described. These key diagnostic symptoms of depression were clearly experienced by all our participants. However, we observed compounding and indirect routes linking LTCs with the cognitive and emotional burden of depression; moreover, thoughts of death were particularly prominent within the depression and CHD group.

Universal cognitive and emotional symptoms

Participants, regardless of LTC, had an overwhelming sense of disinterest in the world around them; they described a deep-rooted sadness, hopelessness, worthlessness and low self-esteem. Some felt as though they were being punished.

For peer review only

"You get what you deserve in life. And I don't deserve nothing. You know, I wasn't a good father when I was young. I was selfish. And it's come back and bit me up the bum, bit me on the bum. ..." **(P.12, depression only, male).**

Compounding effects of LTC on cognitive and emotional depressive symptoms

For some, the combination of the LTC and the depression led to a compounding effect on the cognitive and emotional depressive symptoms. In this way, the LTC directly contributed to the participants' experience of depression. This was particularly observed in those with T2D and arthritis.

"I'm sitting there and taking the blood and giving myself injections and some days I just think, you know, how come it's come to this? And I know how it's come to this because, you know, I'm not managing my life in any sensible way, and I know that." **(P.25, depression and T2D, male).**

For participants with severe arthritic pain, pain was seen as an all-consuming and debilitating experience, which led to misery.

"I'm getting pain back in my right knee and I'm going to have to eventually have that one done. It's really, oh god, what does that feel like? When I'm not feeling … oh ... it feels like having been almost injected with negativity." **(P.28, depression and arthritis, female).**

For P.32 her depression and the functional limitations imposed by her arthritis were difficult to unravel, with both acting in synergy to lead to a sense of hopelessness:

"I'm also physically unable to do as much as I would like to do in terms of just - housework and shopping and it's difficult to pull it apart which is leading the sort of feelings of hopelessness and you know is it worth it and I can't, I might as well just stay in bed." **(P.32, depression and arthritis, female).**

Indirect effects of LTC upon cognitive and emotional depressive symptoms through affecting enjoyment of activities

For some of our LTC participants, the depression was a greater burden than their LTC, so cognitive and emotional depressive symptoms were experienced regardless of their LTC. In this way, some of our LTC participants experienced feelings of anhedonia, worthlessness, guilt, sadness, etc., similarly to the depression

For peer review only - http://bmjopen.bmj.com/site/about/guidelines.xhtml

only participants. However, for others, the LTC (notably T2D and arthritis) fed into the experience of cognitive and emotional symptoms by removing their enjoyment of previously pleasurable activities.

An example comes from P.2, whose diabetes directly contributed to his feelings of anhedonia. This participant was particularly concerned about managing his blood glucose levels during social situations.

> "You are part of it because you are invited, you all go out, you are all friends, you have known one another for years and everything is there, but you don't feel as if you are really taking part if you get what I am saying. So that's the part of the mood I have found since I have been diagnosed with diabetes and that's the thing that holds me back from properly being 100% committed to enjoying it." **(P.2, depression and T2D, male).**

Pain also featured heavily in the descriptions of altered lifestyles recounted by our arthritis participants, perpetuating feelings of anhedonia.

> "That's, and I do enjoy my food, I do enjoy cooking. I mean I haven't had a dinner party now for over a year. I have always enjoyed doing that. But that's, and it's like washing up, you know I can do so much, […] but you know like my washing up is in stages. I will do this first, then go and sit down, come back and do that lot, the next lot, then go and sit down. […]. That is where the arthritis and depression, they are like, they join forces." **(P.41, depression and arthritis, female).**

Indirect effects of LTC on cognitive and emotional symptoms by causing a loss of control and independence

Financial insecurity and unequal personal relationships were experienced by many of our participants, irrespective of LTC, and had contributed to feelings of being out of control. Deteriorating physical health (particularly for those with arthritis) and increasingly frailty, had also led to feelings of loss of control for some. This was linked to feelings of loss of independence, which in turn led to feelings of depression.

> "Some of my clothes are high up, I can never reach them unless somebody is there, that makes me depressed having to rely on somebody all the time." **(P.4, depression and arthritis, female).**

Thoughts of dying

For peer review only - http://bmjopen.bmj.com/site/about/guidelines.xhtml

Suicidal ideation, thoughts of death (but not suicide) and actual suicide attempts were disclosed in participants with and without LTCs. Although the presence of an LTC did not influence suicidal thoughts, having CHD was an important leveller for these participants. These participants lived with the unpredictable nature of their illness and the threat of sudden and irreversible loss of health:

> "Even now I still think about it because when I just took a deep breath here, I felt a bit of a twinge there and it's when I got told about it, it was very disturbing because I was shown the blockage […] and although I've been reassured that it won't go up, block my heart or anything like that, but it makes you wonder because you never know what might happen." **(P.30, depression and CHD, male).**

For P.44 his brush with death due to having a heart attack has given him a greater acceptance of death:

> "Well I just didn't give a shit about anything anymore, you know, apparently according to them I died twice, so I didn't give a shit no more about it, death doesn't scare me no more." **(P.44, depression and T2D, male).**

For others with CHD, they were also having to confront death, but they discussed their fear surrounding a prolonged or sudden death:

> "I keep saying to my sister, 'I don't care if I die actually in my sleep that will be fine. But if I don't die and I can't move, like I said to you if I can't move and I can't get up and can't call that horrifies me.' That is enough to, well you know." **(P.22, depression and CHD, female).**

> "So, since that day, perhaps I have, it wasn't so much when I had the heart bypass, but since the [aortic] aneurism and everything else, […] I worry that every time I go to bed, I don't know if I'm going to wake up. You know. So, I worry about that." **(P.30, depression and CHD, male).**

Theme 4: Depression symptoms thwart self-care

In this theme we draw on the ways in which the experience of depression impeded our participants' ability to take care of themselves; many described not washing or brushing their teeth, not wanting to cook proper meals, and retreating from their social network. This was apparent for all our participants but added an extra layer of complexity to participants with an LTC.

For peer review only - http://bmjopen.bmj.com/site/about/guidelines.xhtml

Depression leads to lack of motivation to self-care

Depression was associated with an apathy which led to a loss of interest in behaviours that might lead to improved self-care. This was true of behaviours that might improve the depression itself such as engaging in social activities and exercising, as well as those important for managing LTCs.

The impact of depression on attempts to control diabetes could lead to self-neglect, both in terms of medication adherence and adherence to medical appointments.

> "I suppose the most obvious impact is there are days when I just can't be bothered to take my medication, both the medication for the depression and the insulin for my diabetes and I know it's stupid […]." **(P.25, depression and T2D, male).**

> "there are times now when I wouldn't not turn up for an appointment, absolutely, I just […] But yes, there are times when I just think "what's the bloody point?" in dealing with daily stuff." **(P.25, depression and T2D, male).**

Exercise was seen as an important self-care behaviour for both mental and physical health among our participants. However, P.32 described how her lack of motivation to exercise was contributing to her physical decline, which in turn compounded her feelings of depression.

> "As for the physical side of things, again, I suspect they would like [me] to say, I'll lose weight, get more exercise and it's very difficult when you don't want to go out of the house, particularly I haven't the confidence to go into a gym or to a swimming pool, it's not really, you know. […] So, and the other fear is, of course, the older I get, the general, you know, aging and infirmities, you know, yeah, it worries me greatly. You know, and it doesn't help the depression to lift. **(P.32, depression and arthritis, female).**

Social withdrawal prevents self-care behaviours

Many participants described how their attempts to cope had been thwarted by the depression itself. This was particularly true for those whose depression had led to social withdrawal. The retreat from daily life saw

For peer review only

participants becoming withdrawn from both the outside world and their family and friends**.** Social withdrawal impacted on participants' management of their LTC. This was observed particularly in those with T2D and arthritis.

> "I did find it hard to leave the house sometimes, you know. You'd just sort of eat what was there rather than go shopping, you know." **(P.27, depression and T2D, female).**

> "Well they said because of the way, it's not helped, because I stopped, I wouldn't get out of the house, I was staying in my room, that can affect arthritis because then you are letting it seize up more." **(P.3, depression and arthritis, female).**

As seen with those experiencing a lack of motivation, social withdrawal also negatively impacted participants' willingness to engage with medical professionals (both for depression and/or the LTC).

> "I mean even the doctor, I have got to phone her tomorrow, they are going to arrange for the nurse to come out because I am overdue blood tests and blood pressure and that […]. Because she knows that I just don't want to leave the house again." **(P.3, depression and arthritis, female).**

Theme 5: Requires wilful effort to manage

The experience of depression was described as an active process, requiring an individual to find ways to cope. For some with an LTC, efforts to cope with their depression were thwarted by the functional limitation imposed by their illness. Controlling the LTC was also important to help manage the depression.

Universal coping strategies

While many of our participants struggled to cope, nearly all engaged in some strategies to navigate their current situation such as the use of alcohol and food, medical treatments, and drawing on friendships and family members for support. Often this included attempts to mask feelings as a means to find comfort; alcohol in particular was frequently described as a way to relax and forget.

> "But what I have done, and I have been guilty of, is purchasing alcohol and trying to blot it out with the alcohol." **(P.23, depression and arthritis, female).**

For peer review only - http://bmjopen.bmj.com/site/about/guidelines.xhtml

Many participants were engaged with healthcare professionals for depression management. For some anti-depressants had been beneficial, making their symptoms less severe. However, for others, side effects or fears surrounding dependency prevented their use.

> "Most of us are given medication, which makes you zombie, anyway. It doesn't help you. It just block your pains, you know. You are numb. You are not sorting out. And how long are you going to use those medication?" **(P.24, depression and T2D, female).**

LTCs impede efforts to cope with depression

Having an LTC prevented some participants from engaging with coping strategies to help manage their depression. For those with arthritis this included being unable to take part in activities that promote wellbeing, including hobbies.

> "But I think, yeah, so coping, well I am not walking as much as I want to, I have stopped going, I used to go to water workout, water aerobics every week and I have stopped doing that, partly because of the difficulty of getting into my bathers and afterwards drying myself." **(P.1, depression and arthritis, female).**

As P.32 described, the combination of her depression and arthritis makes coping with each insurmountable:

> "You know so the depression makes you lack motivation to, perhaps, I say modify some of the effects of the arthritis, but the arthritis also, in limiting you, makes the depression seem worse. Together it seems like a huge, insurmountable problem because if, on their own, they might be manageable, […]. So it is, yes, it impedes any kind of self-care or therapy, if you like, together."

Controlling the LTC improves depression

Interestingly across all LTC groups, those who successfully managed to engage with strategies to cope with their illness, saw benefits to their mental health through feeling an increased sense of control/self-efficacy. For example, failure to self-manage insulin dosages appropriately negatively impacted P.25's mood, while being in control of his T2D also helped him self-manage his depression.

For peer review only - http://bmjopen.bmj.com/site/about/guidelines.xhtml

> "But I carry glucose around with me. … I think we were away somewhere and, but I was completely useless for about two hours and I suppose I, you know, I had a moment of clarity about the need to keep on top of it properly, because I sort of knew that if I didn't, not only would I feel crap, physically, the idea of feeling crap physically was depressing." **(P.25, depression and T2D, male).**

Interestingly, P.30 with CHD found the unpredictable nature of his cardiac symptoms very distressing, but the use of his own blood pressure monitor helped him regain a sense of control.

> "this is my life saver, my BP [blood pressure] machine, I do it every day, […] I was told 140 over 80, right, yes, 140, 80, so once it's within this, I go to sleep a bit better. If I didn't know what my BP was, I'd be worried even more." **(P.30, depression and CHD, male).**

For some participants the physical limitations imposed by their LTC led to social isolation. This was particularly apparent for those with arthritis. If participants received support with things that their arthritis prevented (for example housework, food shopping), this was seen as positive for managing their depression, allowing them to live well within the limits of the LTC.

> "So yeah, [lady friend's name] and [man friend's name] took me out yesterday to go to Aldi, it's the first time since February that I have gone out to the shops." **(P.3, depression and arthritis, female).**

Discussion

This paper explored the lived experience of depression in primary care patients with and without a comorbid physical LTC (CHD, T2D and arthritis). Using thematic analysis, we generated five overarching themes. 'Shaped by the course of time' revealed a shifting, non-linear experience of depression in which the experience was defined by earlier life experiences as well as more minor, cumulative events. For some, the LTC diagnosis had caused catastrophic upheaval to participants' lives; for these individuals the LTC was capable of triggering depression (both first-ever or a recurrent episode). For many, other life events were more prominent to determining the course of depression; for these participants the LTC was coincidental, running alongside the depression but not triggering new episodes. 'Somatisation' highlighted the physical experience of depression

For peer review only - http://bmjopen.bmj.com/site/about/guidelines.xhtml

in our participants; for many, these symptoms were as dominant as the cognitive and emotional symptoms. Somatic symptoms were universally experienced across all our groups, in particular feelings of tiredness and fatigue and changes to eating habits. Where LTCs did interact with the somatic symptoms, this was mainly driven by arthritic pain contributing to and compounding these symptoms. 'Cognitive and emotional burden' explored the impact of mental symptoms, such as feelings of worthlessness, guilt, punishment, anhedonia and sadness, on participants. LTCs compounded these cognitive and emotional experiences as well as indirectly contributing to a worsening of these symptoms by affecting participants' enjoyment of previously pleasurable activities and causing a loss of control and independence. Thoughts of dying were frequently experienced, and prominent in patients with CHD where the unpredictability of future health was a psychological challenge. Theme 4, 'Depression symptoms thwart self-care' examined how depression itself acts as a barrier to engagement in self-care behaviours. Loss of motivation and social withdrawal and were key aspects of the depressive experience which thwarted attempts for LTC self-management (particularly those with T2D). Finally, 'Requires wilful effort to manage' revealed universal engagement in many coping strategies, particularly attempts to mask depressive feelings such as the use of alcohol and food. However, LTCS (particularly arthritis) did impede participants' ability to cope with depression, while successful management of the LTC had a positive impact on mood.

Findings from quantitative studies continue to make attempts to understand the relationship between depression in LTCs, with the causal model thought to be bidirectional.[9] Our findings supported the notion that the LTC was capable of triggering depression; however, this was restricted to those whose LTC has caused major life upheaval (e.g. loss of employment, relationship breakdown). It has been estimated that around 80% of initial depressive episodes occur after a stressful life event;[24] and that the severity of stressors required to precipitate recurrent depressive episodes decreases over time.[25] None of our participants described their depression acting as a precursor to their LTC. In fact, for many, the LTC was experienced as coincidental to the onset of depression; this is a view that has been supported by a previous qualitative meta-synthesis.[15]

For peer review only - http://bmjopen.bmj.com/site/about/guidelines.xhtml

The difference in the experience of depression in those with and without an LTC has not been studied previously using qualitative research. Quantitative research has identified differences in terms of symptom presentation, chronicity and timing of symptoms, and treatment responsivity.[4] Our findings have added to this by revealing that while the symptoms of depression (somatic, cognitive, affective) were universally experienced by those with and without depression, the LTC contributed to the burden of these symptoms by either directly provoking them (e.g. arthritic pain leading to tears of frustration) or by indirectly leading to negative mood by undermining the enjoyment of partaking in previously pleasurable activities as well as reducing individuals' sense of independence. T2D and arthritis were key in this regard since both illnesses were associated with lifestyle changes which negatively impacted the participants' depression, increasing feelings of anhedonia, sadness, guilt and worthlessness. Previous research has explored the impact of arthritis on leisure activities, finding that functional and physical constraints imposed by the illness can affect self-esteem.[26] Our findings extend this to the experience of those with arthritis *and* depression, suggesting that these limitations worsen the symptoms of depression. Participants with diabetes also saw a compounding effect of their physical condition on their mental health. This was particularly true for those who described being distressed by T2D and its management. Indeed, diabetes distress is now recognised as a separate disorder to major depression [27] which has been linked to feelings of burden, difficulties adhering to medication regimes and depression.[28]

Loss of control and lack of independence featured heavily in the accounts of our participants with arthritis; whereby functional limitations imposed by the LTC indirectly impacted depression by removing individual's sense of control and autonomy. Loss was a key theme identified in the qualitative UPBEAT-UK study of patients with depression and CHD, where patients described not only loss of health but also interpersonal loss.[12] Health deterioration featured heavily in the accounts of all our participants with a physical LTC, and while thoughts of dying were universally experienced across groups, our CHD group revealed a strong sense of an unpredictable and uncontrollable death, which was not apparent in those with T2D and arthritis. This realistic fear underpinned the experience of depression in these participants.

For peer review only - http://bmjopen.bmj.com/site/about/guidelines.xhtml

Our findings also observed differences in the experience of depression in participants' ability to self-care and to cope with their mental health. Social withdrawal and lack of motivation were commonly experienced in all our participants. We described how these features of depression thwart attempts to engage with self-care behaviours such as personal hygiene, meal preparation etcetera, as well as LTC (particularly T2D) specific illness-management behaviours such as adhering to medication and attending hospital appointments. While depression has been associated with poor medication adherence in previous quantitative studies of T2D and CHD patients [29,30], there has been a lack of qualitative studies describing the hows and whys of this phenomenon; more research in this area is warranted.

Findings from our study revealed the adoption of common coping behaviours across all our participants such as using alcohol and food to mask symptoms and drawing on expert sources of support; this supports findings from earlier work.[12] However, importantly we observed that the presence of an LTC interacted with participants' attempts to manage their depression. Those with an LTC, and in particular those with increasing disability from their arthritis, were prevented from engaging with coping mechanisms they had previously adopted to promote mental wellbeing. On the upside, positive self-management of the LTC improved feelings of control and self-efficacy and in so doing improved depressed mood. Depressed patients have previously been reported to benefit most from self-efficacy gains from LTC self-management training;[31] the importance of this construct for therapeutic purposes within the modern care setting of depression and LTC patients, requires future investigation.

Our findings have a number of important implications for care models such as the NHS' IAPT-LTC programme.[32] Our findings suggest that any care model of depression in LTC needs to take into account four overarching conclusions based on our data: (1) Not all participants with depression and LTC view their mental and physical health as interconnected; therefore there should be allowances in care plans for separate treatment (this is a view endorsed by Knowles and colleagues).[10] (2) Key features of depression that affect LTC management and need to be considered when planning treatment and referring to psychological services are social withdrawal and lack of motivation to self-manage or access healthcare. (3) Key features of LTCs that

For peer review only - http://bmjopen.bmj.com/site/about/guidelines.xhtml

worsen depression that should be discussed during care planning are pain, the unpredictability of future health and progressive disability. (4) Positive self-management of LTC could improve feelings of control and self-efficacy and therefore mood, and should be encouraged.

A strength of our study was in the recruitment of participants from GP practices ensuring all our participants had received clinical diagnoses of their health conditions. To understand the differences in depression across different LTCs we excluded participants with more than one LTC of interest and with a serious or life-limiting diagnosis such as cancer, stroke or dementia. Due to the increased risk of physical illness with age, we recruited participants within an age bracket of 55 to 75 years; this enabled us to focus on the experiences of adults in mid to late life. The study was confined to North and North West London; however, we recruited from across contrasting areas to elicit a range of experiences. Despite our best efforts, unfortunately our arthritis sample was predominately female (90%); however this may partly reflect the greater incidence of osteoarthritis in women in the UK.[33] Our depression and CHD group was small (n=5); therefore our findings may not reflect the diversity of views that a larger sample might have attained. Also, as part of our reflexive practice, we noted that as researchers our experience and identities as female academic researchers working in London, of White (HR, RF, GB) and mixed (LP) ethnic background, is likely to have shaped our understanding, prioritisation, and interpretation of the data. The trustworthiness of our analysis, however, has been enhanced through researcher triangulation, drawing on the multiple expertise of the research team. Moreover, we used multiple levels to the analysis process from use of the reflective diary, through to the generation of themes, and the comparative exercise through the charting of codes. Participant checking was not undertaken which could have added a useful check to our interpretive work.

In conclusion, the results of our face-to-face interview study of depression in those with and without a physical LTC revealed that overall, key diagnostic symptoms of depression (anhedonia, sadness etc.) were experienced by all our participants regardless of LTC. However, the LTC did interact with depression by compounding somatic, cognitive, and emotional symptoms, increasing disability and reducing independence, and hindering

For peer review only

attempts at coping with mental illness. Our findings have implications for the development and fine-tuning of mental health and LTC care programmes.

Contributorship statement:

LP was responsible for funding acquisition and conceived the study design. GB and RF advised on the study methods. LP and HR collected the data. LP and RF led the data analysis. GB and HR contributed to the data analysis. LP wrote the initial manuscript draft. RF, GB and HR edited the manuscript.

Competing interests:

The authors have no competing interests to declare.

Funding:

LP and HR were funded by the UK Economic and Social Research Council (ES/N001478/1) for this project.

Data sharing statement:

The data for the study are archived in the UK Data Service.[18]

For peer review only - http://bmjopen.bmj.com/site/about/guidelines.xhtml

References

1 Smith DJ, Nicholl BI, Cullen B, *et al.* Prevalence and Characteristics of Probable Major Depression and Bipolar Disorder within UK Biobank: Cross-Sectional Study of 172,751 Participants. *PLOS ONE* 2013;**8**:e75362. doi:10.1371/journal.pone.0075362

2 Machado MO, Veronese N, Sanches M, *et al.* The association of depression and all-cause and cause-specific mortality: an umbrella review of systematic reviews and meta-analyses. *BMC Med* 2018;**16**:112. doi:10.1186/s12916-018-1101-z

3 Roest AM, de Jonge P. The heart of the matter: in search of causal effects of depression on somatic diseases. *BMC Med* 2018;**16**:147. doi:10.1186/s12916-018-1144-1

4 Poole L, Dickens C, Steptoe A. The puzzle of depression and acute coronary syndrome: reviewing the role of acute inflammation. *J Psychosom Res* 2011;**71**:61–8. doi:10.1016/j.jpsychores.2010.12.009

5 de Miranda Azevedo R, Roest AM, Hoen PW, *et al.* Cognitive/affective and somatic/affective symptoms of depression in patients with heart disease and their association with cardiovascular prognosis: a meta-analysis. *Psychol Med* 2014;**44**:2689–703. doi:10.1017/S0033291714000063

6 Poole L, Jackowska M. The association between depressive and sleep symptoms for predicting incident disease onset after 6-year follow-up: findings from the English Longitudinal Study of Ageing. *Psychol Med* 2018;:1–10. doi:10.1017/S0033291718001290

7 Dickens C, McGowan L, Percival C, *et al.* New onset depression following myocardial infarction predicts cardiac mortality. *Psychosom Med* 2008;**70**:450–5. doi:10.1097/PSY.0b013e31816a74de

8 White J, Zaninotto P, Walters K, *et al.* Duration of depressive symptoms and mortality risk: the English Longitudinal Study of Ageing (ELSA). *Br J Psychiatry J Ment Sci* 2016;**208**:337–42. doi:10.1192/bjp.bp.114.155333

9 Gold SM, Köhler-Forsberg O, Moss-Morris R, *et al.* Comorbid depression in medical diseases. *Nat Rev Dis Primer* 2020;**6**:1–22. doi:10.1038/s41572-020-0200-2

10 Knowles SE, Chew-Graham C, Adeyemi I, *et al.* Managing depression in people with multimorbidity: a qualitative evaluation of an integrated collaborative care model. *BMC Fam Pract* 2015;**16**:32. doi:10.1186/s12875-015-0246-5

11 Coventry PA, Hays R, Dickens C, *et al.* Talking about depression: a qualitative study of barriers to managing depression in people with long term conditions in primary care. *BMC Fam Pract* 2011;**12**:10. doi:10.1186/1471-2296-12-10

12 Simmonds RL, Tylee A, Walters P, *et al.* Patients' perceptions of depression and coronary heart disease: a qualitative UPBEAT-UK study. *BMC Fam Pract* 2013;**14**:38. doi:10.1186/1471-2296-14-38

13 Raison CL, Miller AH. Is Depression an Inflammatory Disorder? *Curr Psychiatry Rep* 2011;**13**:467–75. doi:10.1007/s11920-011-0232-0

14 Poole L, Steptoe A. The combined association of depressive symptoms and C-reactive protein for incident disease risk up to 12 years later. Findings from the English Longitudinal Study of Ageing (ELSA). *Brain Behav Immun* 2020;**88**:908–12.

For peer review only - http://bmjopen.bmj.com/site/about/guidelines.xhtml

15 DeJean D, Giacomini M, Vanstone M, *et al.* Patient Experiences of Depression and Anxiety with Chronic Disease. *Ont Health Technol Assess Ser* 2013;**13**:1–33.

16 Gask L, Macdonald W, Bower P. What is the relationship between diabetes and depression? A qualitative meta-synthesis of patient experience of co-morbidity. *Chronic Illn* 2011;**7**:239–52. doi:10.1177/1742395311403636

17 Hughes R, Huby M. The construction and interpretation of vignettes in social research. *Soc Work Soc Sci Rev* 2012;**11**:36–51.

18 [dataset] Poole, L., Rowlands, H. The Mood and Health Study, 2018-2019. [data collection]. *UK Data Service* 2021. doi:10.5255/UKDA-SN-854780. https://beta.ukdataservice.ac.uk/datacatalogue/studies/study?id=854780

19 Braun V, Clarke V. Using thematic analysis in psychology. *Qual Res Psychol* 2006;**3**:77–101. doi:10.1191/1478088706qp063oa

20 Braun V, Clarke V. Can I use TA? Should I use TA? Should I not use TA? Comparing reflexive thematic analysis and other pattern-based qualitative analytic approaches. *Couns Psychother Res* 2021;**21**:37–47.

21 Braun V, Clarke V. To saturate or not to saturate? Questioning data saturation as a useful concept for thematic analysis and sample-size rationales. *Qual Res Sport Exerc Health* 2021;**13**:201–16. doi:10.1080/2159676X.2019.1704846

22 Ritchie J, Spencer L. Qualitative data analysis for applied policy research. *Qual Res Companion* 2002;:305–29.

23 Marcu A, Black G, Vedsted P, *et al.* Educational differences in responses to breast cancer symptoms: A qualitative comparative study. *Br J Health Psychol* 2017;**22**:26–41. doi:https://doi.org/10.1111/bjhp.12215

24 Mazure CM. Life stressors as risk factors in depression. *Clin Psychol Sci Pract* 1998;**5**:291–313.

25 Tennant C. Life Events, Stress and Depression: A Review of Recent Findings. *Aust N Z J Psychiatry* 2002;**36**:173–82. doi:10.1046/j.1440-1614.2002.01007.x

26 Wikström I, Jacobsson LT, Arvidsson B. How people with rheumatoid arthritis perceive leisure activities: a qualitative study. *Musculoskeletal Care* 2005;**3**:74–84. doi:10.1002/msc.28

27 Kreider KE. Diabetes Distress or Major Depressive Disorder? A Practical Approach to Diagnosing and Treating Psychological Comorbidities of Diabetes. *Diabetes Ther* 2017;**8**:1–7. doi:10.1007/s13300-017-0231-1

28 Tanenbaum ML, Kane NS, Kenowitz J, *et al.* Diabetes distress from the patient's perspective: Qualitative themes and treatment regimen differences among adults with type 2 diabetes. *J Diabetes Complications* 2016;**30**:1060–8. doi:10.1016/j.jdiacomp.2016.04.023

29 Carney RM, Freedland KE, Eisen SA, *et al.* Major depression and medication adherence in elderly patients with coronary artery disease. *Health Psychol* 1995;**14**:88–90. doi:10.1037/0278-6133.14.1.88

30 Gonzalez JS, Safren SA, Cagliero E, *et al.* Depression, Self-Care, and Medication Adherence in Type 2 Diabetes: Relationships across the full range of symptom severity. *Diabetes Care* 2007;**30**:2222–7. doi:10.2337/dc07-0158

For peer review only

For peer review only - http://bmjopen.bmj.com/site/about/guidelines.xhtml

31 Jerant A, Kravitz R, Moore-Hill M, *et al.* Depressive Symptoms Moderated the Effect of Chronic Illness Self-Management Training on Self-Efficacy. *Med Care* 2008;**46**:523–31.

32 NHS England. The Improving Access to Psychological Therapies (IAPT) Pathway for People with Long-term Physical Health Conditions and Medically Unexplained Symptoms. 2018.https://www.england.nhs.uk/wp-content/uploads/2018/03/improving-access-to-psychological-therapies-long-term-conditions-pathway.pdf

33 Swain S, Sarmanova A, Mallen C, *et al.* Trends in incidence and prevalence of osteoarthritis in the United Kingdom: findings from the Clinical Practice Research Datalink (CPRD). *Osteoarthritis Cartilage* 2020;**28**:792–801. doi:10.1016/j.joca.2020.03.004

For peer review only

For peer review only - http://bmjopen.bmj.com/site/about/guidelines.xhtml

Table 1: Characteristics of the sample, stratified by group

	Whole sample (N= 41) *M±SD or N(%)*	Depression only (n = 12) *M±SD or N(%)*	Depression and CHD (n = 5) *M±SD or N(%)*	Depression and T2D (n = 10 *M±SD or N(%)*	Depression and arthritis (n = 14) *M±SD or N(%)*
Sex (female)	24(58.5)	6(50.0)	1(20.0)	4(40.0)	13(92.9)
Age (years)	63.5(5.5)	62.4(5.5)	66.0(4.0)	61.0(5.2)	65.1(5.9)
Married/cohabiting	17(41.5)	6(50.0)	2(40.0)	5(50.0)	4(28.6)
Ethnicity:					
White British	27(65.9)	10(83.3)	2(20.0)	6(60.0)	9(64.3)
Indian	3(7.3)			2(20.0)	1(7.1)
Pakistani	1(2.4)		1(20.0)		
Black Caribbean	1(2.4)		1(20.0)		
Other White background	7(17.1)	2(16.7)	1(20.0)	1(10.0)	3(21.4)
Other Asian background	2(4.9)			1(10.0)	1(7.0)
Employment status:					
Employed full-time	9(22.0)	3(25.0)	1(20.0)	3(30.0)	2(14.3)
Employed part-time	1(2.4)	1(8.3)			
Self-employed	7(17.1)	3(25.0)			4(28.6)
Retired	15(36.6)	4(33.3)	3(60.0)	3(30.0)	5(35.7)
Unemployed	4(9.8)	1(8.3)		3(30.0)	
Volunteer	2(4.9)		1(20.0)		1(7.1)
Disabled	3(7.3)			1(10.0)	2(14.3)
Highest educational qualification - degree or higher	15(36.6)	3(25.0)	3(60.0)	4(40.0)	5(35.7)
Antidepressant usage (yes)	35(85.4)	9(75.0)	4(80.0)	9(90.0)	13(92.9)

For peer review only - http://bmjopen.bmj.com/site/about/guidelines.xhtml

Figure 1 Caption *(figure itself attached as separate .jpg file)*

Figure 1. Schematic diagram of theme structure.

N.B. LTC: Long term condition.

For peer review only

For peer review only - http://bmjopen.bmj.com/site/about/guidelines.xhtml

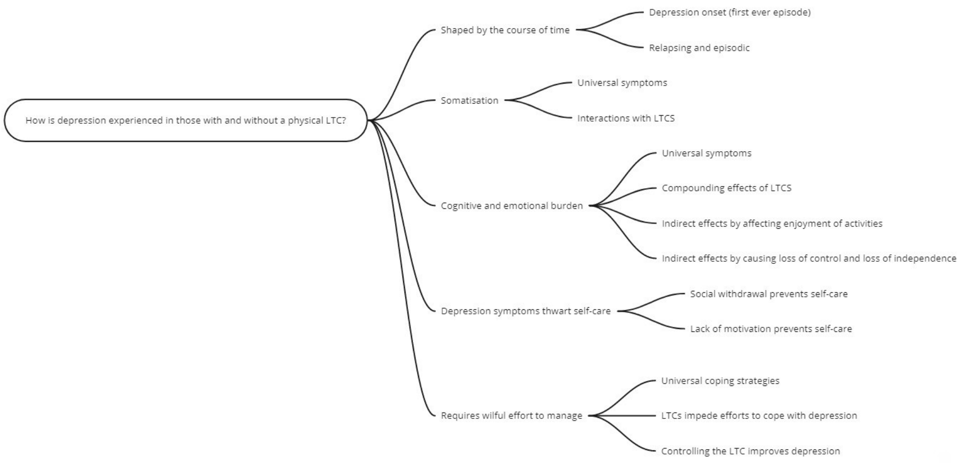

Figure 1. Schematic diagram of theme structure.
N.B. LTC: Long term condition.

372x187mm (96 x 96 DPI)

Supplementary file: Appendix 1 - Vignettes

Scenario 1

Sarah has good days and bad, but on a bad day she feels so sad and miserable that she just can't stand it. It seems to completely take over her head like she's in a fog. She feels really disappointed in herself for feeling this way, despite all the good things she has in her life. Sometimes when she's by herself she cries and even has thoughts of killing herself just to try and be anywhere but here. She feels completely worthless.

Scenario 2

Most days Sam wakes up and feels so tired he wishes he could just stay in bed. In fact he feels so tired most of the time that he doesn't want to do anything. He sleeps longer than he used to and doesn't have the energy to do anything. Sometimes he doesn't even feel like eating. He's even lost weight without trying to. His partner tries to show him affection and wants a kiss and a cuddle, but he just feels numb and not interested at all.

Scenario 3

Jason finds it really hard to feel positive about the future at the moment, it's like he has nothing to look forward to anymore. He finds it difficult to concentrate and to make decisions these days too. He feels so frustrated and trapped by the way he feels. His friends tell him he should snap out of it, but he just can't. He wishes he didn't have to feel like this, it makes him feel like such a failure.

Scenario 4

Most days Clare feels like she is being punished for something she must have done in a past life. And then she feels guilty for thinking like this. She seems to worry about her health all the time and her aches and pains get on top of her. She doesn't enjoy things the way she used to, in fact she doesn't really get any pleasure out of anything anymore. She finds it hard to leave the house some days. Everything seems a bit pointless these days, if she's being completely honest.

Scenario 5

Susan has always enjoyed her food, but for the past couple of months she's noticed she's been putting on some extra weight. She finds she craves her favourite foods and is comfort eating to make herself feel a bit better. She's not sure she is feeling any better though, in fact she's sleeping really badly and often wakes up several times in the night. Her sleep is restless and she wakes up tired making each day feel like it drags.

For peer review only - http://bmjopen.bmj.com/site/about/guidelines.xhtml

The Mood and Health Study

Interview Schedule – Depression

Introduction

Review of Participant Information Sheet

Consent form

Timing and confidentiality

My name is [insert name] and I am a researcher. I'm not a doctor or a psychologist and I don't have any clinical contact with your team. I'm interested in finding out from you what your experiences have been with your mood, and there are no right answers.

Vignettes

To start I'd like you to look at what some other people have experienced, and then we'll talk about them a bit. Firstly, I'd like you to start by sorting the cards into two piles, those which you think are relevant to you and those which aren't.

**Card sort**	Can you talk me through how you have sorted the cards?<br>Has anything like this ever happened to you?<br>Have you ever felt these sorts of things before?<br>How do you think this person's experience is different from yours?

Depression

**Precipitating events**	Take me back, can you tell me about when you first noticed changes to your mood?

For peer review only - http://bmjopen.bmj.com/site/about/guidelines.xhtml

Prompts	• Can you remember feeling differently?<br>• How did it feel?<br>• Was there anything else you think may have contributed to your low mood?<br>• How do you think that experience relates to how you feel today?
**Diagnosis**	Can you talk me through the support you received during this time?
Prompts	• E.g. family, friends, GP?<br>• How did you find out you had depression? E.g. Who told you? When?<br>• How did you feel when you found out it was depression?
**Timeline**	How has your mood been since then?
Prompts	• How has you mood changed over time?
**Symptoms**	Can you tell me about a time when you felt your depression was at its worst?
Prompts	• How did it feel?<br>• How long did it last?<br>• Can you tell me how most days are for you at the moment?<br>• What's a good day for you?<br>• How long have you been feeling this way?<br>• Are there times when you don't feel this way? How long does this last?
**Management of depression**	Are you receiving any treatment for your depression at the moment?
Prompts	• Can you tell me about the care you have received for your depression from doctors?<br><br>e.g. who (GP, clinic nurse/doctor)<br>e.g. where (GP clinic, hospital clinic, community/third sector)<br>e.g. when (routine/booked appointments, length of appointments)

For peer review only - http://bmjopen.bmj.com/site/about/guidelines.xhtml

	e.g. what (antidepressants, counselling)<br>• Do you find it easy to talk to your doctor about your concerns?<br>• How did you find the treatment?<br>• How helpful has this treatment been? E.g. what was most helpful and least helpful?
**Self-care**	• Can you talk me through anything you do yourself that helps you cope better?
Prompts	• For example, exercise, eating habits, alcohol consumption, smoking, acceptance, distractions/hobbies, wallowing. Have any of these been helpful to you?<br>• How do these things make you feel?

Wrapping up

Is there anything else you would like to tell me?

How have you found this interview?

Do you have any questions?

Thank you for your time.

Give sources of further support handout.

Give baseline questionnaire.

Provide participant with £10 retail voucher.

For peer review only - http://bmjopen.bmj.com/site/about/guidelines.xhtml

The Mood and Health Study

Interview Schedule – Depression and Physical Illness

Introduction

Review of Participant Information Sheet

Consent form

Timing and confidentiality

My name is [insert name] and I am a researcher. I'm not a doctor or a psychologist and I don't have any clinical contact with your team. I'm interested in finding out from you what your experiences have been with your mood and health, and there are no right answers.

Vignettes

To start I'd like you to look at what some other people have experienced, and then we'll talk about them a bit. Firstly, I'd like you to start by sorting the cards into two piles, those which you think are relevant to you and those which aren't.

**Card sort**	Can you talk me through how you have sorted the cards?<br>Has anything like this ever happened to you?<br>Have you ever felt these sorts of things before?<br>How do you think this person's experience is different from yours?

Depression

**Precipitating events**	Take me back, can you tell me about when you first noticed changes to your mood?

For peer review only - http://bmjopen.bmj.com/site/about/guidelines.xhtml

Prompts	• Can you remember feeling differently?<br>• How did you feel?<br>• Was there anything else you think may have contributed to your low mood?<br>• How do you think that experience relates to how you feel today?
**Diagnosis**	Can you talk me through the support you received during this time?
Prompts	• E.g. family, friends, GP?<br>• How did you find out you had depression? E.g. Who told you? When?<br>• How did you feel when you found out it was depression?
**Timeline**	How has your mood been since then?
Prompts	• How has you mood changed over time?
**Diagnosis of physical illness (if not covered above)**	Can you tell me how you found out about your [arthritis/heart disease/diabetes]?
Prompts	• How long ago was this?<br>• Who told you? Where were you?<br>• How did you feel when you found out?<br>• Did you find out about your [arthritis/heart disease/diabetes] before or after you found out you had depression? How did you find this time?
**Symptoms**	Can you tell me about a time when you felt your depression was at its worst?
Prompts	• How did it feel?<br>• How long did it last?<br>• Can you tell me how most days are for you at the moment?<br>• What's a good day for you?

For peer review only - http://bmjopen.bmj.com/site/about/guidelines.xhtml

	• How long have you been feeling this way?<br>• Are there times when you don't feel this way? How long does this last?
**Interaction**	Can you tell me about how your [arthritis/heart disease/diabetes] affects your life now?
	• How does your [arthritis/heart disease/diabetes] make you feel?<br>• How do your depression and [arthritis/heart disease/diabetes] affect each other?<br>• Have you ever found it difficult to cope with [arthritis/heart disease/diabetes]?<br>e.g. coping with the symptoms, treatments?
**Management**	Are you receiving any treatment for your depression at the moment?
Prompts	• Can you tell me about the care you have received for your depression from doctors?<br><br>e.g. who (GP, clinic nurse/doctor)<br>e.g. where (GP clinic, hospital clinic, community/third sector)<br>e.g. when (routine/booked appointments, length of appointments)<br>e.g. what (antidepressants, counselling)<br><br>• How did you find the treatment?<br>• How helpful has this treatment been? E.g. what was most helpful and least helpful?<br>• How easy have you found it to get help for your depression?
	What treatments have you had for your [arthritis/heart disease/diabetes]?
Prompts	e.g. who (GP, clinic nurse/doctor)<br>e.g. where (GP clinic, hospital clinic)<br>e.g. when (more than one treatment?)<br>e.g. what (self-management courses, medication, surgery?)

For peer review only - http://bmjopen.bmj.com/site/about/guidelines.xhtml

	• Do you find it easy to talk to your doctor about your concerns?<br>• How did you find the treatment?<br>• How helpful has this treatment been? E.g. what was most helpful and least helpful?<br>• How do you feel treatment of your [arthritis/heart disease/diabetes] has affected your depression?<br>• **[Arthritis and angina only]** How have the treatments helped with your pain?
**Self-care**	• Can you talk me through anything you do yourself that helps you cope better?
Prompts	• For example, exercise, eating habits, alcohol consumption, smoking, acceptance, distractions/hobbies, wallowing. Have any of these been helpful to you?<br>• How do these things make you feel?

Wrapping up

Is there anything else you would like to tell me?

How have you found this interview?

Do you have any questions?

Thank you for your time.

Give sources of further support handout.

Give baseline questionnaire.

Provide participant with £10 retail voucher.

For peer review only - http://bmjopen.bmj.com/site/about/guidelines.xhtml

COREQ (COnsolidated criteria for REporting Qualitative research) Checklist

A checklist of items that should be included in reports of qualitative research. You must report the page number in your manuscript where you consider each of the items listed in this checklist. If you have not included this information, either revise your manuscript accordingly before submitting or note N/A.

Topic	Item No.	Guide Questions/Description	Reported on Page No.
**Domain 1: Research team and reflexivity**			
Personal characteristics			
Interviewer/facilitator	1	Which author/s conducted the interview or focus group?	7
Credentials	2	What were the researcher's credentials? E.g. PhD, MD	7
Occupation	3	What was their occupation at the time of the study?	7
Gender	4	Was the researcher male or female?	7
Experience and training	5	What experience or training did the researcher have?	7
Relationship with participants			
Relationship established	6	Was a relationship established prior to study commencement?	7
Participant knowledge of the interviewer	7	What did the participants know about the researcher? e.g. personal goals, reasons for doing the research	7
Interviewer characteristics	8	What characteristics were reported about the inter viewer/facilitator? e.g. Bias, assumptions, reasons and interests in the research topic	7
**Domain 2: Study design**			
Theoretical framework			
Methodological orientation and Theory	9	What methodological orientation was stated to underpin the study? e.g. grounded theory, discourse analysis, ethnography, phenomenology, content analysis	8
Participant selection			
Sampling	10	How were participants selected? e.g. purposive, convenience, consecutive, snowball	6
Method of approach	11	How were participants approached? e.g. face-to-face, telephone, mail, email	6
Sample size	12	How many participants were in the study?	9
Non-participation	13	How many people refused to participate or dropped out? Reasons?	9
Setting			
Setting of data collection	14	Where was the data collected? e.g. home, clinic, workplace	6
Presence of non-participants	15	Was anyone else present besides the participants and researchers?	6
Description of sample	16	What are the important characteristics of the sample? e.g. demographic data, date	9
Data collection			
Interview guide	17	Were questions, prompts, guides provided by the authors? Was it pilot tested?	7
Repeat interviews	18	Were repeat inter views carried out? If yes, how many?	N/A
Audio/visual recording	19	Did the research use audio or visual recording to collect the data?	7
Field notes	20	Were field notes made during and/or after the inter view or focus group?	7
Duration	21	What was the duration of the inter views or focus group?	6
Data saturation	22	Was data saturation discussed?	6&8
Transcripts returned	23	Were transcripts returned to participants for comment and/or	24

For peer review only - http://bmjopen.bmj.com/site/about/guidelines.xhtml

Topic	Item No.	Guide Questions/Description	Reported on Page No.
		correction?	
**Domain 3: analysis and findings**			
Data analysis			
Number of data coders	24	How many data coders coded the data?	8
Description of the coding tree	25	Did authors provide a description of the coding tree?	8
Derivation of themes	26	Were themes identified in advance or derived from the data?	8
Software	27	What software, if applicable, was used to manage the data?	8
Participant checking	28	Did participants provide feedback on the findings?	24
Reporting			
Quotations presented	29	Were participant quotations presented to illustrate the themes/findings? Was each quotation identified? e.g. participant number	10-20
Data and findings consistent	30	Was there consistency between the data presented and the findings?	10-20
Clarity of major themes	31	Were major themes clearly presented in the findings?	10-20
Clarity of minor themes	32	Is there a description of diverse cases or discussion of minor themes?	10-20

Developed from: Tong A, Sainsbury P, Craig J. Consolidated criteria for reporting qualitative research (COREQ): a 32-item checklist for interviews and focus groups. *International Journal for Quality in Health Care*. 2007. Volume 19, Number 6: pp. 349 – 357

Once you have completed this checklist, please save a copy and upload it as part of your submission. DO NOT include this checklist as part of the main manuscript document. It must be uploaded as a separate file.

For peer review only
